# The Climatic Niche Diversity of Malagasy Primates: A Phylogenetic Perspective

**DOI:** 10.1371/journal.pone.0011073

**Published:** 2010-06-11

**Authors:** Jason M. Kamilar, Kathleen M. Muldoon

**Affiliations:** 1 Department of Anthropology, Yale University, New Haven, Connecticut, United States of America; 2 Yale Molecular Anthropology Laboratory, Yale University, New Haven, Connecticut, United States of America; 3 Department of Anatomy, Dartmouth Medical School, Hanover, New Hampshire, United States of America; 4 Department of Anthropology, Dartmouth College, Hanover, New Hampshire, United States of America; Texas A&M University, United States of America

## Abstract

**Background:**

Numerous researchers have posited that there should be a strong negative relationship between the evolutionary distance among species and their ecological similarity. Alternative evidence suggests that members of adaptive radiations should display no relationship between divergence time and ecological similarity because rapid evolution results in near-simultaneous speciation early in the clade's history. In this paper, we performed the first investigation of ecological diversity in a phylogenetic context using a mammalian adaptive radiation, the Malagasy primates.

**Methodology/Principal Findings:**

We collected data for 43 extant species including: 1) 1064 species by locality samples, 2) GIS climate data for each sampling locality, and 3) the phylogenetic relationships of the species. We calculated the niche space of each species by summarizing the climatic variation at localities of known occurrence. Climate data from all species occurrences at all sites were entered into a principal components analysis. We calculated the mean value of the first two PCA axes, representing rainfall and temperature diversity, for each species. We calculated the *K* statistic using the Physig program for Matlab to examine how well the climatic niche space of species was correlated with phylogeny.

**Conclusions/Significance:**

We found that there was little relationship between the phylogenetic distance of Malagasy primates and their rainfall and temperature niche space, i.e., closely related species tend to occupy different climatic niches. Furthermore, several species from different genera converged on a similar climatic niche. These results have important implications for the evolution of ecological diversity, and the long-term survival of these endangered species.

## Introduction

With the development of advanced quantitative tools, researchers are now well positioned to examine biological variation in an evolutionary context. This is especially true for behavioral and ecological characteristics, with many ecologists now using an explicitly evolutionary approach to examine ecological diversity [1], [2]. It has become widely accepted that closely related species occupy similar ecological niches, and that these niches diverge as the evolutionary time between species increases [3], [4], [5], [6]. Several recent studies, for example, of Costa Rican plants [7] and Neotropical frogs [8], have supported this pattern. However, different evolutionary scenarios may yield contrasting results [9]. For instance, several authors have argued that there should be little relationship between niche similarity and phylogenetic distance in situations where the rate of evolutionary divergence is higher early in a clade's history than later on, such as occurs in adaptive radiations [1], [9], [10], [11]. This hypothesis is supported by several studies of *Anolis* lizards in the Caribbean, where closely related species occupy divergent niches [12], [13]. The ubiquity of this pattern of phylogenetic signal is currently unknown.

Phylogenetic signal has most recently been defined as the degree to which any trait is correlated with a phylogeny [1], [14]. Strong phylogenetic signal is defined as a significant correlation between the degree of relatedness among species and their biological similarity, with trait similarity decreasing as phylogenetic distance increases. This pattern is potentially due to constant-rate genetic drift. [1], [9]. Alternatively, weak phylogenetic signal may result from divergent selection [1], [9].

In this study, we test the hypothesis that there is phylogenetic signal in the climatic niche of Malagasy primates. We first quantify the climatic niche space of Malagasy primates based on known localities of species occurrence and GIS climate data using an ecological niche modeling approach. This approach provides a quantitative summary of species' climatic niches that is amenable to statistical analysis and summarizes their known climatic tolerances. Phylogenetic analyses have demonstrated that lemurs evolved by adaptive radiation following a single colonization event to Madagascar [15], [16]. Today, lemurs inhabit nearly every possible climatic and habitat niche in Madagascar, from the highly seasonal, arid spiny deserts of the south, to the aseasonal, humid evergreen rainforests of the east [17]. If closely related species occupy distinct climatic niches, then we expect to find weak phylogenetic signal in the climatic niche space of Malagasy primates as a whole.

Understanding ecological diversity in a phylogenetic context is critical because the assumption that there is a close relationship between ecological and evolutionary similarity is at the foundation of a wide range of biodiversity research, including ecological niche modeling of species ranges in the future [1], [2], [18]. Systematically testing this potential relationship will help us understand the possible future diversity of species. This is especially important for Malagasy primates, as they are a highly diverse and threatened group of vertebrates [19], [20].

## Results

The PCA analysis produced nine components, with the first two accounting for over 70% of the total variance in the original dataset. Four variables related to rainfall patterns loaded heavily on the first principal component: 1) mean annual rainfall (−), 2) precipitation seasonality (+), 3) precipitation in the driest month (−), and 4) precipitation in the warmest quarter (−). Two variables related to temperature variation loaded heavily on the second principal component: 1) temperature seasonality (−) and 2) temperature annual range (−) (Table 1).

**Table 1:** Results of the first two principal components summarizing the climatic niche space of Malagasy primates.

	Factor Loadings	
Variable	Factor 1	Factor 2
Log Annual Mean Temp.	0.706	0.426
Log Isothermality	0.287	0.618
Log Temp. Seasonality	−0.194	−0.951
Log Temp. Annual Range	0.542	−0.743
Log Annual Precip.	−0.745	0.547
Log Precipitation Driest Month	−0.868	−0.077
Log Precipitation Seasonality (CV)	0.761	0.156
Log Precipitation Warmest Quarter	−0.829	0.279
Log Altitude	−0.590	−0.327
Eigenvalue	3.836	2.533
Proportion of variance explained	0.426	0.281

**Figure 1:**
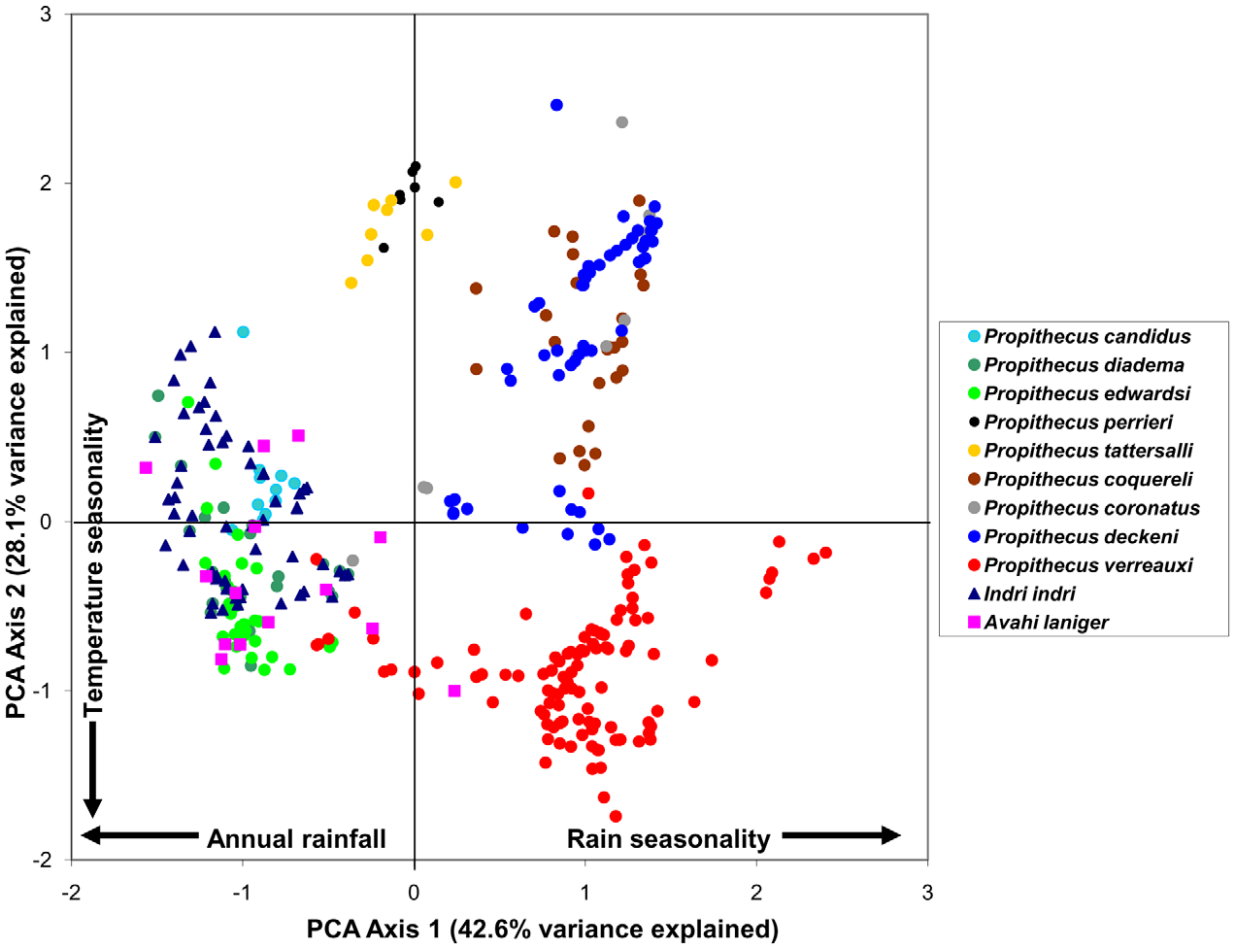
Plot of PCA scores representing the multivariate climatic niche space of Indriidae species based on their known localities. The plot illustrates the same multivariate space as figures 2–5.

By examining the first two components in a bivariate plot (Figs. 1–5), several ecological patterns are revealed. Each quadrant of the graph captures sites that exhibit a unique combination of annual rainfall, rain seasonality and temperature seasonality. The lower right quadrant of the plot represents localities with relatively high levels of seasonality in both mean annual rainfall and temperature, such as localities of the spiny thicket of southern Madagascar (e.g. Beza Mahafaly Special Reserve). Alternatively, sites in the lower left quadrant exhibit high temperature seasonality, but lower levels of rain seasonality (corresponding with high annual rainfall). Many of these sites are found in the humid evergreen forests of eastern Madagascar, such as Ranomafana National Park. Furthermore, plots of the PCA scores revealed high levels of climatic niche space diversity within several genera, especially those containing numerous species (Figs. 1–5). For example, *Propithecus*, *Eulemur*, and *Microcebus* occupy nearly all possible environmental niche spaces as defined by the variables and taxa included in this study. In contrast, members of less diverse genera such as *Varecia and Indri* occupy relatively narrow niches.

**Figure 2 pone-0011073-g002:**
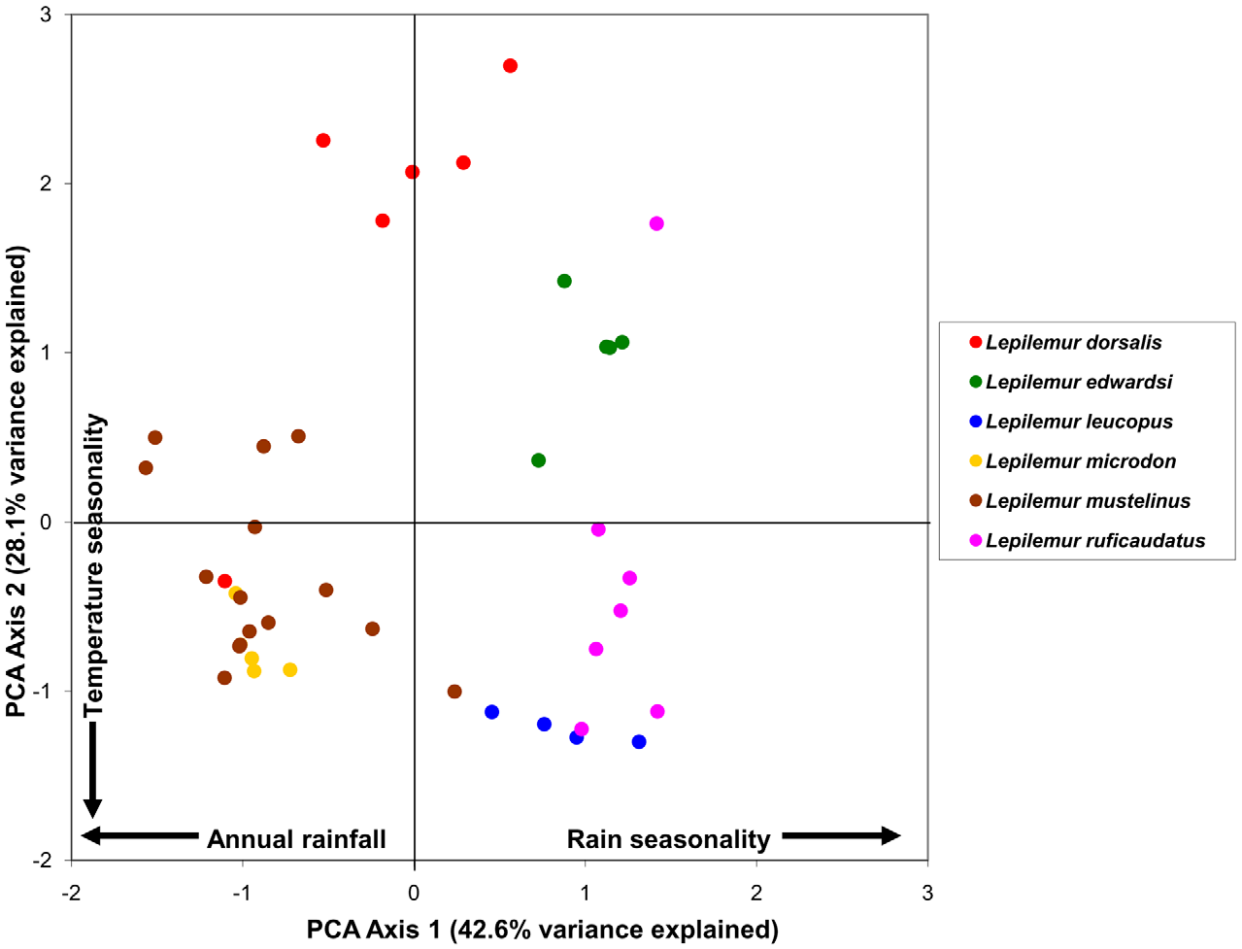
Plot of PCA scores representing the multivariate climatic niche space of Lepilemuridae species based on their known localities. The plot illustrates the same multivariate space as figures 1, 3–5.

**Figure 3 pone-0011073-g003:**
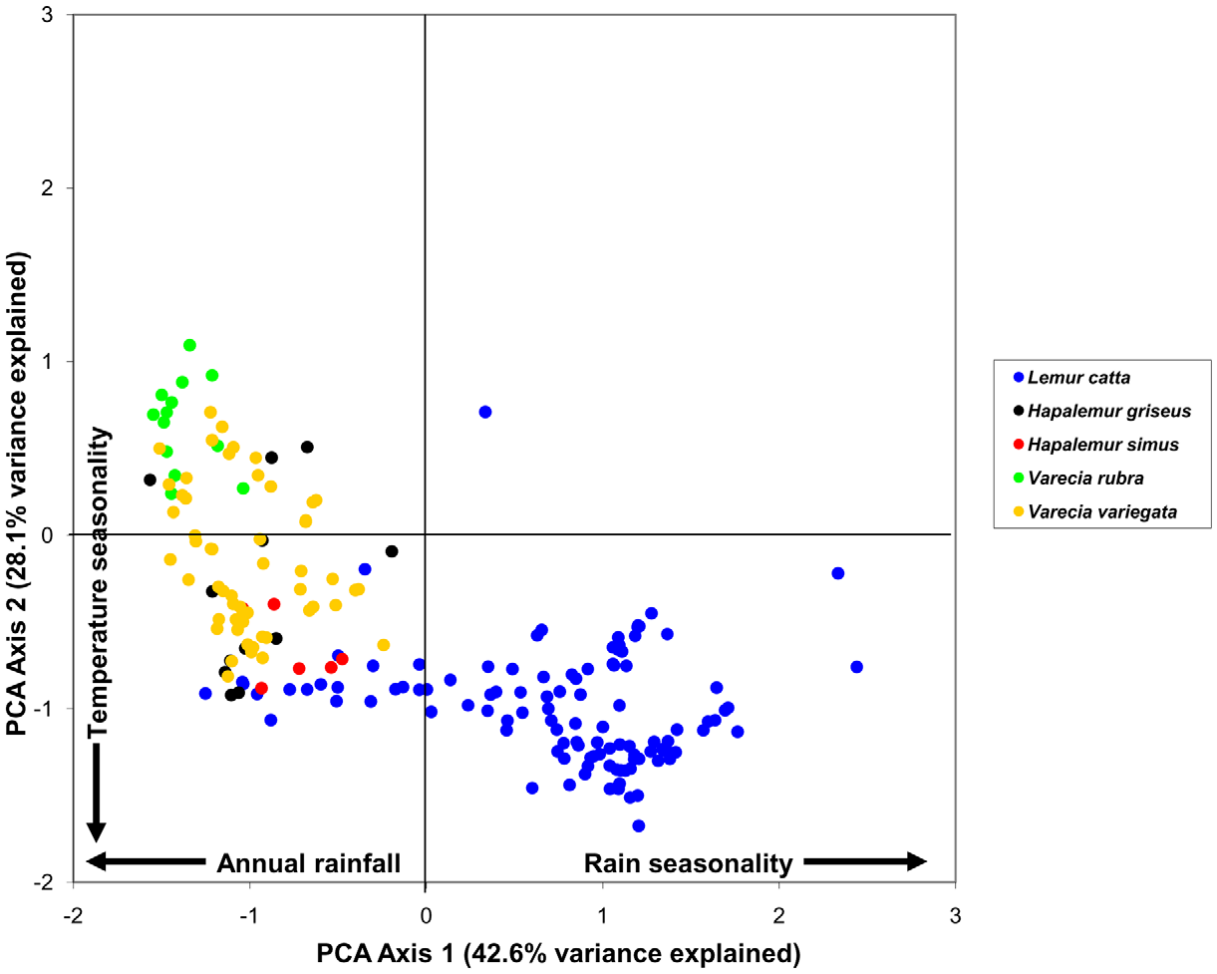
Plot of PCA scores representing the multivariate climatic niche space of Lemuridae (excluding *Eulemur*) species based on their known localities. The plot illustrates the same multivariate space as figures 1–2, 4–5.

**Figure 4 pone-0011073-g004:**
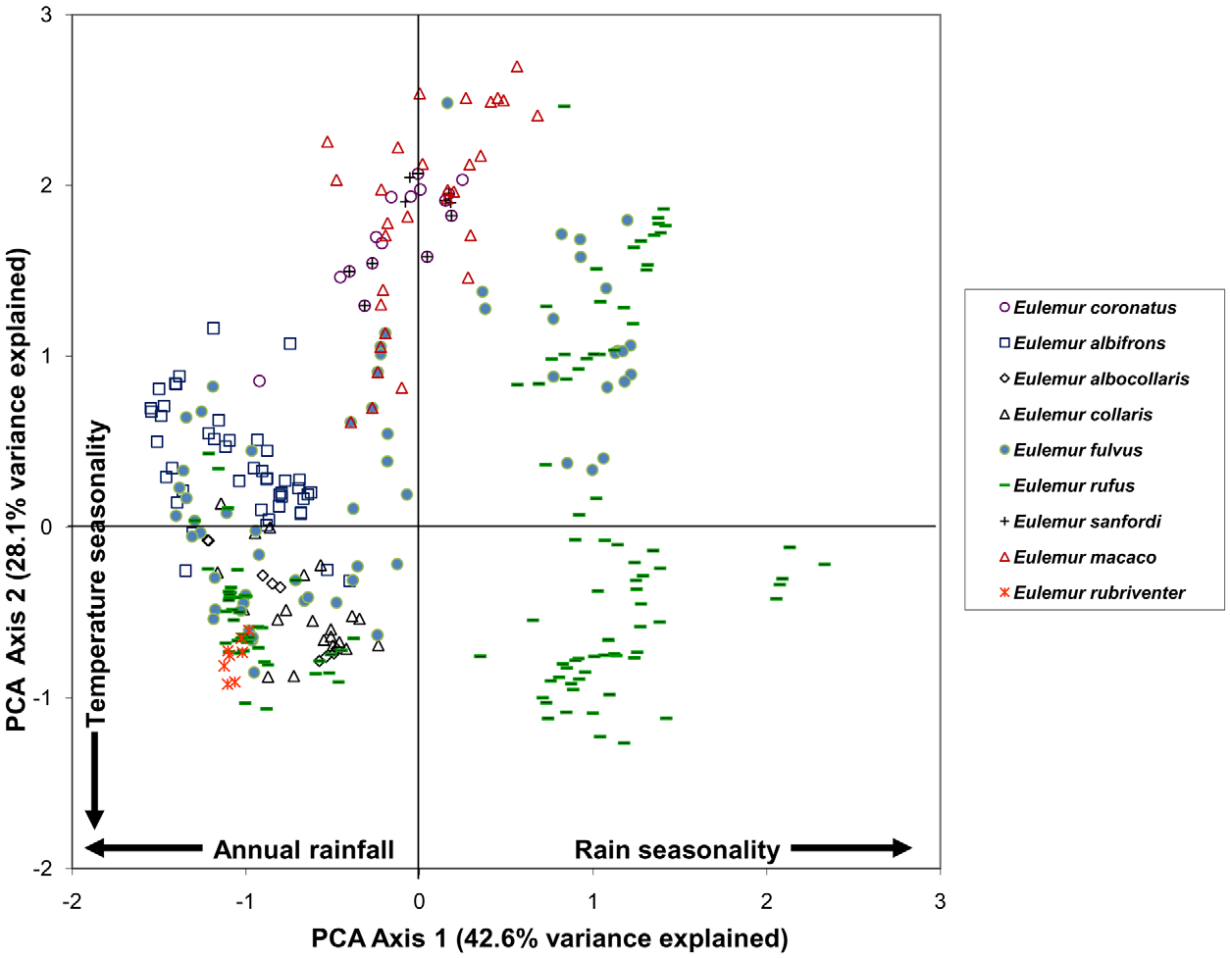
Plot of PCA scores representing the multivariate climatic niche space of *Eulemur* species based on their known localities. The plot illustrates the same multivariate space as figures 1–3, 5.

**Figure 5:**
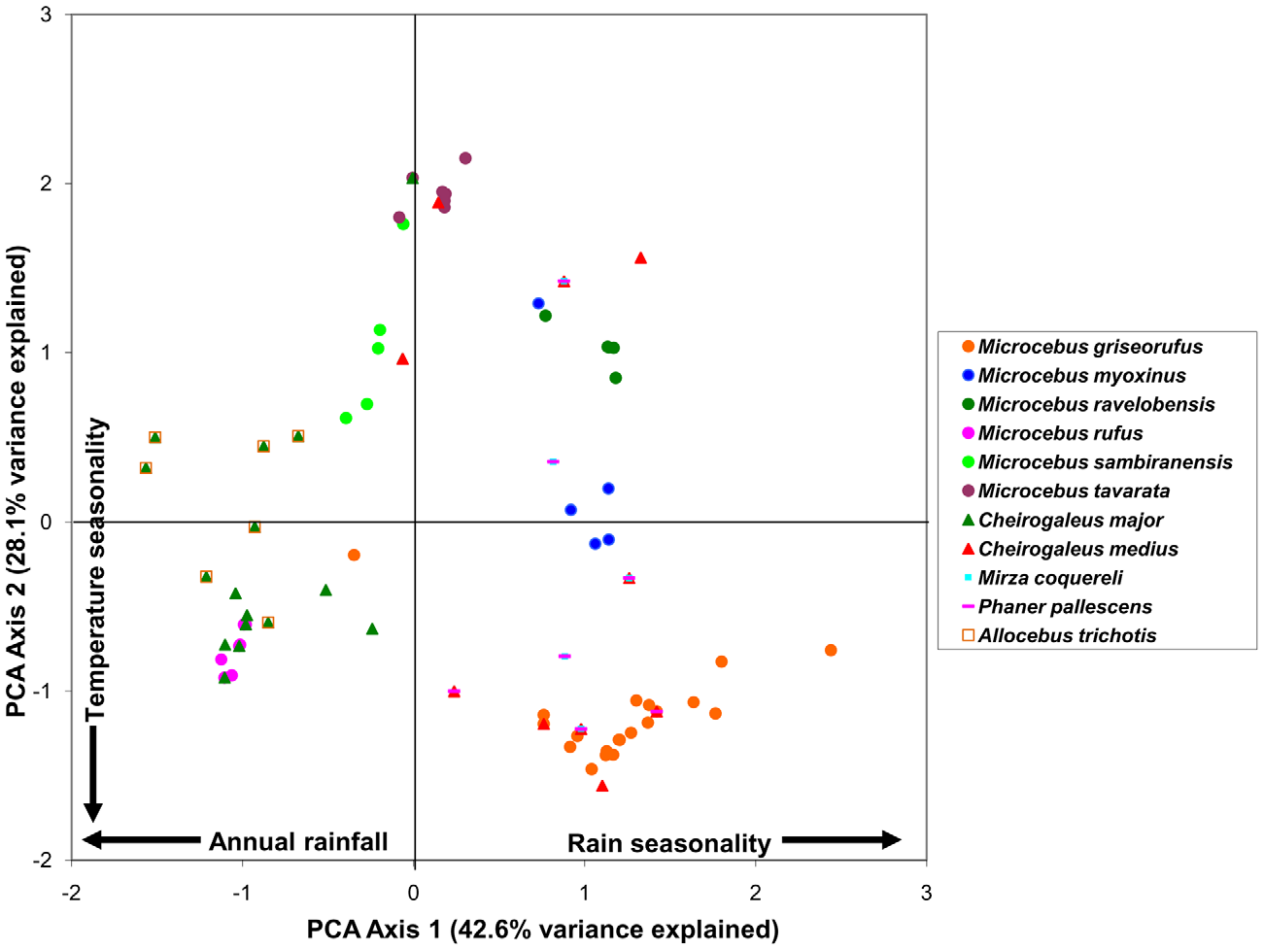
Plot of PCA scores representing the multivariate climatic niche space of Cheirogaleidae species based on their known localities. The plot illustrates the same multivariate space as figures 1–4.

**Figure 6:**
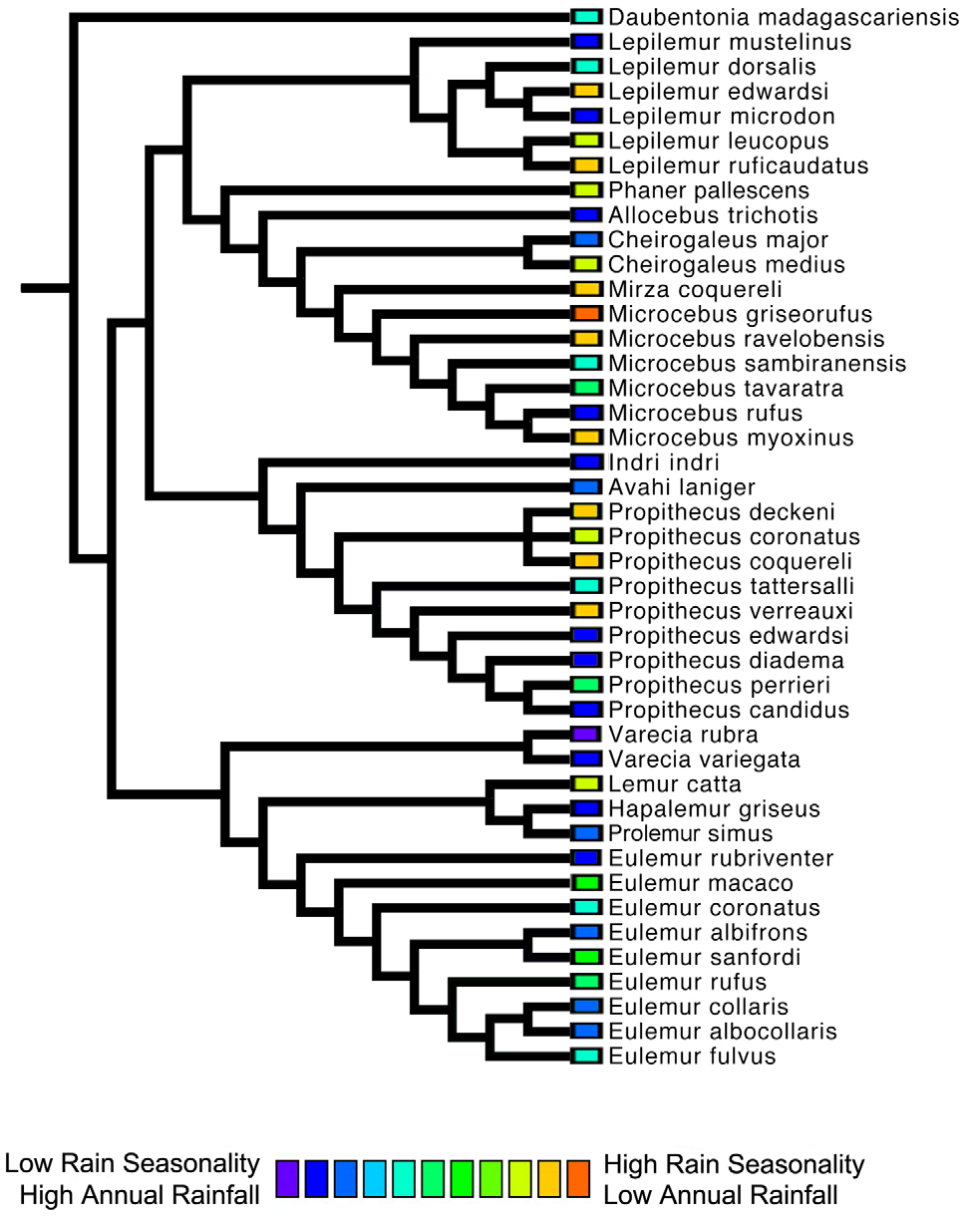
A phylogenetic perspective of Malagasy primate niche space as defined by rainfall variables (principal component 1). There is a no significant phylogenetic signal in this niche axis for all species comparisons (p = 0.116), or within the Lepilemuridae (p = 0.428), Cheirogaleidae (p = 0.265), or Lemuridae (p = 0.207) families. The Indriidae display the highest level of phylogenetic signal in the rainfall niche axis (p = 0.031). Also note that several distantly related species converge on a similar niche space.

**Figure 7:**
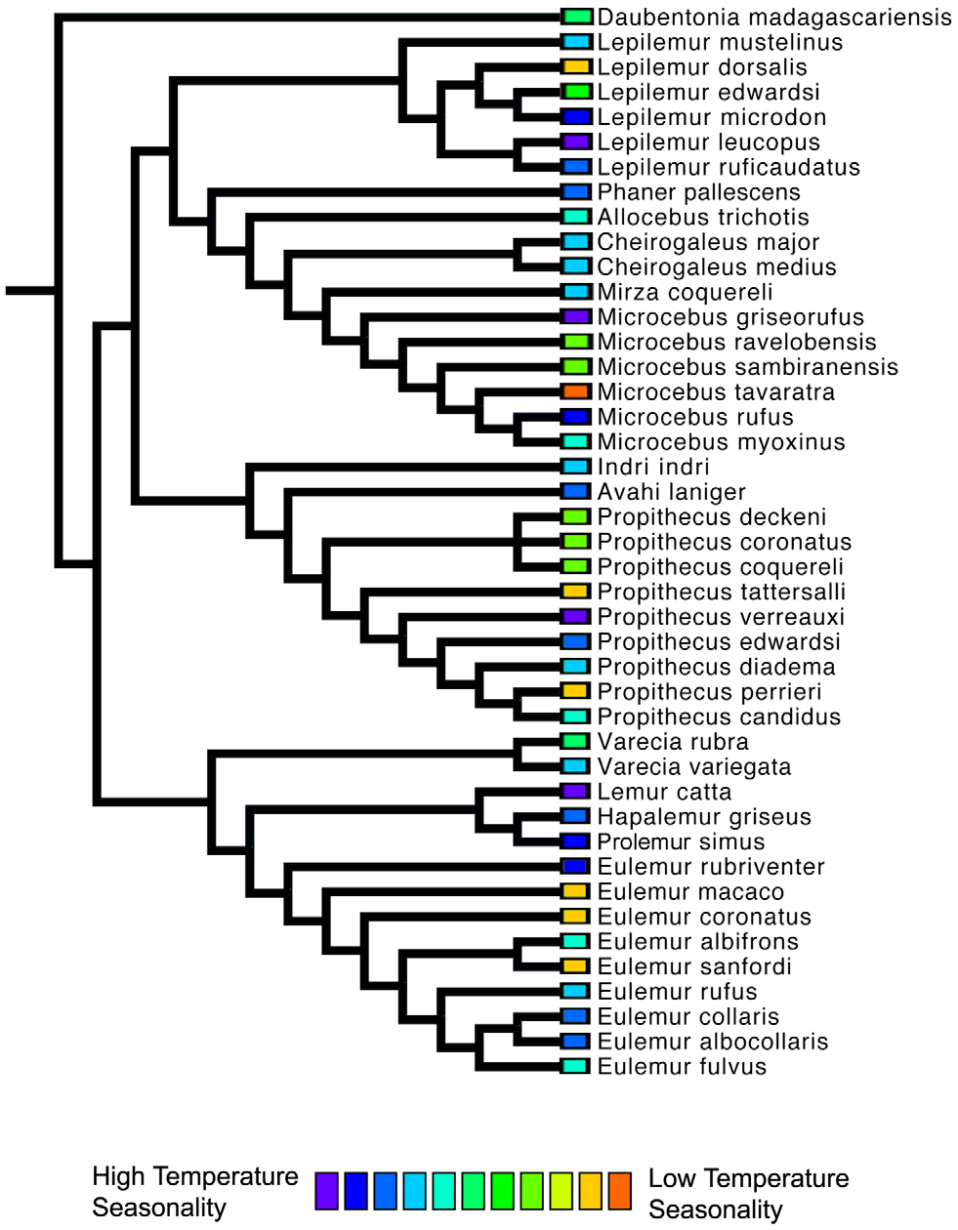
A phylogenetic perspective of Malagasy primate niche space as defined by temperature variables (principal component 2). There is no significant phylogenetic signal in this niche axis for all species comparisons (p = 0.568),or within families (Lepilemuridae (p = 0.415), Cheirogaleidae (p = 0.327), Indriidae (p = 0.304), Lemuridae (p = 0.431). Also note that several distantly related species converge on a similar niche space.

We found no significant phylogenetic signal in the first two climatic niche axes, regardless of the taxonomic group analyzed. For all Malagasy primates (n = 43), we found no significant phylogenetic signal in PC1 (*K* = 0.294, p = 0.116), which is related to rainfall patterns (Fig. 6) or in PC2 (*K* = 0.245, p = 0.429), which is related to temperature variables (Fig. 7). Patterns of phylogenetic signal were similar at the family level, except for the indriids. Significant phylogenetic signal was absent in the rainfall and temperature niche axes for both the cheirogaleids (PCA 1: *K* = 0.265, p = 0.836 and PCA 2: *K* = 0.414, p = 0.327; n = 11) and the lemurids (PCA 1: *K* = 0.430, p = 0.207 and PCA 2: *K* = 0.346, p = 0.431; n = 14). Both the lepilemurids (n = 6) and indriids (n = 11) displayed high *K* values for niche axis one (*K* = 0.825 and *K* = 0.934,), yet these values were statistically significant for the indriids only (p = 0.428 and p = 0.031, respectively). Both families exhibited no significant phylogenetic signal in the temperature niche axis (*K* = 0.507, p = 0.415 and *K* = 0.436, p = 0.304, respectively). It is important to keep in mind that samples sizes at the family level were relatively small, resulting in lower statistical power compared to using the total dataset.

## Discussion

Previous studies have suggested that a lack of phylogenetic signal in ecological data is typical of island faunas [10], [11], [12], [13], given that insular settings are well known for their adaptive radiations [21]. Our results from the climatic niche analysis of Malagasy primates are congruent with these findings and are among the first to demonstrate this pattern in island-restricted mammals. Analyses including all species revealed no correlation between phylogenetic relatedness and similarity in climatic niche. However, significant phylogenetic signal was detected at the family level for one group, the indriids. Phylogenetic signal within indriids could be the result of neutral (i.e. random) climatic niche evolution within this family, given that the *K* value was nearly 1, which is the degree of correlation between trait divergence and phylogenetic distance that is expected under Brownian motion. Alternatively, this result could relate to recent taxonomic revisions of the genus *Propithecus*, in which several subspecies were elevated to specific status [22]. The division of a single widespread species into multiple allopatric species (as would result from taxonomic inflation [23]) may “artificially” increase phylogenetic signal, by reflecting patterns of geographic variation [1]. A future test of alternative taxonomic hypotheses is possible using an ecological niche modeling approach [24].

A closer look at patterns of environmental tolerance among Malagasy primates reveals interesting evolutionary relationships. Although some sister species differ little in their climatic niche (e.g., *Varecia rubra* and *V. variegata* for rainfall tolerances; *Propithecus deckeni* and *P. coronatus* for temperature tolerances), in several cases sister taxa are markedly dissimilar (e.g., *Cheirogaleus medius* and *C. major* for rainfall tolerances; *Lepilemur microdon* and *Lepilemur edwardsi* for temperature tolerances). Furthermore, some distantly related species are more similar in their climatic niche (e.g., *Indri indri* and *Varecia variegata* for rainfall and temperature tolerances) than closely related species. Such similarities between less-related species could be the result of two different evolutionary processes. Non-sister species with similar climatic niches may have retained the ancestral niche through time, and therefore be more similar than expected based on their phylogenetic relatedness [e.g., phylogenetic niche conservatism, sensu 1]. Alternatively, non-sister species might have independently derived the same niche through convergent evolution. Unlike many previous ecological niche modeling studies [25], [26], we did not use phylogenetic methods to infer the niche of hypothetical ancestral taxa because of the low precision of ancestral reconstructions [27], [28], [29], [30]. As a consequence, we are less able to make statements about the direction in which niche evolution occurred.

Nonetheless, visualizing the niche space of lemurs provides a view of the distribution of each species in relation to climatic, and thus habitat, parameters. For example, the ecological space occupied by the genus *Eulemur* is nearly equivalent to the space occupied by all the Malagasy primates included in our analysis (Fig. 4). However, within the genus *Eulemur*, species vary in total niche space occupied. *Eulemur rufus*, a broadly-distributed species that occurs in both the seasonal dry deciduous forests of the west and humid rainforests of the east, has very large niche breadth. In contrast, *Eulemur sanfordi*, which is restricted to the rainforests of northeastern Madagascar, is much more limited in terms of total niche space. Furthermore, an interesting situation occurs between the sister taxa *E. fulvus*, and the group including *E. collaris* and *E. albocollaris*, in which localities containing the latter group fall within the *E. fulvus* ecospace. We interpret this to indicate that the climatic niches of *E. collaris* and *E. albocollaris* are more specialized than, and are nested within, the *E. fulvus* niche. Without inferring ancestral niches, the direction in which this evolutionary change occurred is difficult to discern. The sister taxa to the *E. fulvus – E. collaris – E. albocollaris* group is *E. rufus*, a species with a broad niche similar to that of *E. fulvus*. However, the sister taxa to this species group (*E. albifrons and E. sanfordi*), have comparatively narrow niches.

As has been previously demonstrated for both the recently extinct giant lemurs and still-extant Malagasy primates [31], [32], our results demonstrate that all families and most genera are widely distributed across the island, with allopatric species that occupy a wide variety of habitats and climatic conditions. In fact, closely-related species occur sympatrically at only a few localities (e.g., *Eulemur mongoz* – *E. fulvus* at Ampijoroa; *E. coronatus – Eulemur sanfordi* at Ankarana; *E. rubriventer – E. fulvus* at Ranomafana). This phenomenon, in which closely related species tend not to occur sympatrically, is unique to the Malagasy primates, where the average phylogenetic distance among species in communities is relatively high compared to primate communities in other regions [33]. In contrast, distantly related Malagasy primate species often converge on a similar climatic niche space (Figs. 6–7). This can be seen in species pairs such as *Propithecus verreauxi* and *Microcebus griseorufus*, which are not closely related, but have a similar geographic distribution.

Our results have important implications for studies of the evolution of community structure in a phylogenetic context. Ecologists have long hypothesized that closely related species have the strongest interspecific interactions. However, in the scenario of adaptive radiation, species may diverge from near relatives such that they interact just as strongly with less related species [21]. Consequently, ecological interactions among distantly related species play an important role in structuring local communities, as has been shown for *Anolis* lizards in Cuba [12].

Investigating the role that adaptation to different climatic niches has played in lemur evolution will contribute greatly to understanding this species-rich clade. However, there are limitations to our interpretations. First, climatic variables capture only one axis of a species overall niche space. Our estimates of niche overlap therefore may not represent cases of true sympatry at the within-site level. An important component of lemur community ecology and evolution is partitioning of habitats within a site [34], [35], [36], [37], [38]. An analysis of additional niche metrics such as diet, activity budget, and vertical habitat use may yield a finer-scale resolution of local distributions. Unfortunately, quantitative data for these variables are unavailable for most localities in our dataset. Second, current species distributions may be a result of recent dispersal, allopatric speciation or local extinction, and not a consequence of distributions actually tracking climatic conditions. Kamilar [39] found that geography (as a proxy for historical processes, such as dispersal) predicted primate species distributions independent of climatic conditions in Madagascar, although environmental factors explained even more variation in lemur community structure independent of geography. Likewise, Muldoon and Goodman [40] found a correlation between Malagasy non-volant mammal community structure and habitat type, suggesting that species ranges have been sorted along environmental gradients. Wilmé et al. [41] hypothesized that allopatric speciation at low altitudes during Quaternary climatic fluctuations explains the distribution of Madagascar's extant diurnal lemurs, although this process may be more complex than initially thought [42].

Paleontological evidence demonstrates that at least 17 species of large-bodied lemurs have become extinct in the past few thousand years [43], [44], resulting in a significant loss of both taxonomic and niche breadth in modern primate communities [31], [45]. The extinct lemurs belonged to five families that comprise clades within extant Lemuriformes [46] and were distributed across Madagascar in several habitats [31], [32]. Paleoenvironmental data is not available for most subfossil localities, and therefore their inclusion in this analysis is not currently possible. Furthermore, range contractions in some still-extant Malagasy primates have been extensive since the Pleistocene [32], [47], [48], presenting a problem for the interpretation sister species that appear to diverge in niche characteristics today. This scenario, for example, is displayed by two species pairs in our data set (e.g., *Lepilemur leucopus* and *L. ruficaudatus*; *Hapalemur griseus* and *Prolemur simus*). However, phylogenetic studies of Malagasy primates demonstrate a tendency for sister species to be found in parapatry [49], suggesting that current lemur distribution patterns maintain a signal that at least partly reflects the original geography of speciation [42].

Third, because of small sample sizes for family-level comparisons, and given the current instability of lemur taxonomy [23], [50], [51], [52], more data are needed to confirm and explore this pattern. Taxon sampling has been shown to affect the performance of statistical tests of trait associations [53]. Despite these caveats, we believe our results are sufficiently robust to suggest a lack of phylogenetic signal in the climatic niche of Malagasy lemurs.

We have demonstrated a pattern of niche divergence among closely related species of Malagasy primates, as has been found in other taxa [12], [13], [25], [26]. It thus appears that phylogenetic signal should not necessarily be the expected outcome of evolutionary diversification [1]. Our results further suggest that climatic diversity plays a key role in generating primate diversity in Madagascar. Key areas in each of the island's climatic regimes must be built into future protected areas Madagascar's exceptional biological diversity is to be preserved [54].

## Materials and Methods

### Data Collection

We collected data from a total of 43 lemur species, comprising 1064 site by species samples (File S1). We follow the taxonomy of Mittermeier et al. (2006). Only species with at least five known localities were included in the study. We obtained the majority of locality data from Wilmé et al. [41]. This dataset was supplemented with published and unpublished data provided by the KMM and field researchers. These datasets contained the latitude and longitude of each site to the 0.01 degree. We followed previous ecological modeling studies by characterizing climatic niche space based on the abiotic conditions that each species is known to tolerate [24], [39], [55]. Species niche spaces are therefore based on the climatic conditions as defined by their known occurrences. Because our dataset is not exhaustive, this measurement represents the minimum known environmental niche space of a species. Because climate is a widely used proxy for habitat in comparative studies [39], [56], [57], [58], the climatic niche space can be viewed as broadly representing the habitat niche space for each species.

We obtained the abiotic variables from the WorldClim GIS climate database [59]. This database contains 19 climate and one topographic variable (altitude) at a ∼1 km resolution. The climate variables include various measures of rainfall and temperature patterns, including mean and extreme annual values. The WorldClim database is created by interpolating weather data gathered during the past 50+ years from over 100 weather stations distributed throughout Madagascar. We used the “extract to point” function in ArcGIS to obtain the climate data for each locality.

Our phylogeny was based on three sources: Mayor et al. [22] for *Propithecus*, Yoder and Heckman [60] for *Microcebus*, and Horvath et al. [61] for the remaining taxa, as well as the tree topology above the species level. We utilized equal branch lengths (i.e. branch lengths set to 1.0) instead of the absolute estimated divergence times between species. This procedure is unlikely to have a large impact on the results due to the fact that the PHYSIG analysis uses a randomization approach (consequently the magnitudes of branch lengths are less important), but was needed because well-established divergence times were unavailable for numerous species.

### Data Analyses

We conducted a principal components analysis (PCA) to quantify lemur niches in multidimensional space. This allows us to account for covariation among variables and reduces the dimensionality of our dataset [62]. Before we conducted the PCA, we constructed a covariation matrix among all variables. Examining the covariation matrix allowed us to minimize the degree of multicolinearity in our dataset by removing variables that displayed r^2^ values greater than 0.85. Although a PCA accounts for covariation among variables, including several variables that are highly correlated can produce spurious results [62]. Using this criterion, we removed 11 variables from our initial dataset. Therefore, our PCA included the following nine variables: 1) annual mean temperature, 2) isothermality ((mean diurnal temperature range/temperature annual range)*100), 3) temperature seasonality (standard deviation*100), 4) temperature annual range (maximum temperature of warmest month-minimum temperature of coldest month), 5) annual precipitation, 6) precipitation during driest month, 7) precipitation seasonality (coefficient of variation of monthly rainfall), 8) precipitation during warmest quarter, and 9) altitude. We log_10_ transformed the variables before being entered into the PCA because the analysis is based on a parametric (e.g. Pearson's) correlation matrix [62]. We produced nine principal components to be certain that we accounted for all of the variation in our original dataset and that all of our components are orthogonal to each other [62].

To visualize niche space in an evolutionary context, we calculated each species mean PCA score for each component, and then plotted these values on a phylogenetic tree using Mesquite [63]. We calculated the degree of phylogenetic signal in niche space by using the *K* statistic presented by Blomberg et al. [14]. This statistic uses a generalized least squares approach to quantify the degree to which closely related species exhibit similar biological characteristics based on the typology and branch lengths of their phylogeny. The *K* statistic is a standardized ratio of the phylogenetic covariance of the species data to the expected covariance produced from Brownian motion. Because *K* is a standardized unit, *K* values based on different tree typologies and branch lengths can be compared. A *K* value of one indicates that the species trait is perfectly correlated to the phylogeny as expected under Brownian motion. Values less than one suggest that closely related species resemble each other less than expected, and values greater than one indicate that related species are more similar to each other than expected under Brownian motion [1], [9], [14]. To test for statistical significance, we used 9999 simulations to compare the *K* statistic generated from the real data to a distribution of randomized values. We calculated the *K* statistic with the Matlab script, PHYSIG.M, available from Ted Garland (UC Riverside). We measured phylogenetic signal for the first two niche axes (i.e. principal components), as these accounted for more than 70% of the variation in the original data. To account for potential differences among the major Malagasy primate clades, we examined the degree of phylogenetic signal in all Malagasy primates, as well as the lepilemurids, cheirogaleids, indriids, and lemurids separately. The one drawback of examining phylogenetic signal at the family level is the smaller number of species in these taxonomic units. Blomberg et al. [14] showed that statistical power declines with sample sizes less than 20. Therefore, some caution should be used when interpreting statistically non-significant results for these analyses because Type II error rates are higher.

## Supporting Information

File S1 Data used in the analyses.
(0.12 MB PDF)
