# Supplementary material for: The Climatic Niche Diversity of Malagasy Primates: A Phylogenetic Perspective
Source: PLoS One. 2010 Jun 11;5(6):e11073. doi: 10.1371/journal.pone.0011073 (PMC2884016; doi:10.1371/journal.pone.0011073)
Supplement: File S1 — Data used in the analyses. (0.12 MB PDF) [file pone.0011073.s001.pdf]

Species	Long	Lat	V1	V2	V3	V4	V5	V6	V7	V8	V9	V10
Allocebus trichotis	48.47	-18.47	18.1	11.1	64	26.15	26.2	9.1	17.1	20.9	15.7	20.9
Allocebus trichotis	48.5	-18.85	20	10.8	63	25.9	28	11.1	16.9	22.8	17.7	22.8
Allocebus trichotis	48.97	-17.43	22.8	10	66	22.49	30	14.9	15.1	25.3	20.6	25.3
Allocebus trichotis	49.43	-14.7	15.7	12.2	71	21.37	23.9	6.8	17.1	18	12.7	18
Allocebus trichotis	49.55	-14.31	21.4	11.4	70	20.57	29	12.8	16.2	23.6	19.3	23.6
Allocebus trichotis	49.73	-16.38	22.4	8.1	61	20.74	29	15.9	13.1	24.9	21.6	24.9
Allocebus trichotis	50.13	-15.52	21.3	8.6	62	20.95	28.2	14.5	13.7	23.7	20.2	23.7
Avahi laniger	46.55	-24.52	22	13.3	65	27.15	31.1	10.9	20.2	25	18.9	25
Avahi laniger	46.88	-22.57	21.3	11.2	63	25.66	29.3	11.6	17.7	23.8	18.2	24
Avahi laniger	47.33	-18.18	20	12.7	67	21.93	28.6	9.8	18.8	22	16.7	22
Avahi laniger	47.44	-20.69	17.2	11	63	25.79	25.4	8	17.4	19.9	13.9	19.9
Avahi laniger	47.44	-20.69	17.2	11	63	25.79	25.4	8	17.4	19.9	13.9	19.9
Avahi laniger	47.47	-21.37	20.4	10.7	61	25.58	28.4	11.1	17.3	23	18.6	23.1
Avahi laniger	47.63	-20.39	17	11	63	25.9	25.1	7.8	17.3	19.7	13.6	19.7
Avahi laniger	47.64	-20.39	17	10.9	62	26.05	25.2	7.8	17.4	19.7	13.6	19.7
Avahi laniger	47.95	-18.49	18.3	11.7	66	24.42	26.5	8.9	17.6	20.8	14.9	20.8
Avahi laniger	48.47	-18.47	18.1	11.1	64	26.15	26.2	9.1	17.1	20.9	15.7	20.9
Avahi laniger	48.5	-18.85	20	10.8	63	25.9	28	11.1	16.9	22.8	17.7	22.8
Avahi laniger	48.97	-17.43	22.8	10	66	22.49	30	14.9	15.1	25.3	20.6	25.3
Avahi laniger	49.43	-14.7	15.7	12.2	71	21.37	23.9	6.8	17.1	18	12.7	18
Avahi laniger	49.43	-14.7	15.7	12.2	71	21.37	23.9	6.8	17.1	18	12.7	18
Avahi laniger	49.55	-14.31	21.4	11.4	70	20.57	29	12.8	16.2	23.6	19.3	23.6
Avahi laniger	50.13	-15.52	21.3	8.6	62	20.95	28.2	14.5	13.7	23.7	20.2	23.7
Cheirogaleus major	46.55	-24.52	22	13.3	65	27.15	31.1	10.9	20.2	25	18.9	25
Cheirogaleus major	46.88	-22.57	21.3	11.2	63	25.66	29.3	11.6	17.7	23.8	18.2	24
Cheirogaleus major	47.43	-20.7	17.9	11.1	64	25.51	26	8.7	17.3	20.6	14.6	20.6
Cheirogaleus major	47.44	-20.68	17.1	11	63	25.73	25.2	7.8	17.4	19.8	13.7	19.8
Cheirogaleus major	47.47	-21.37	20.4	10.7	61	25.58	28.4	11.1	17.3	23	18.6	23.1
Cheirogaleus major	47.63	-20.39	17	11	63	25.9	25.1	7.8	17.3	19.7	13.6	19.7
Cheirogaleus major	47.63	-20.39	17	11	63	25.9	25.1	7.8	17.3	19.7	13.6	19.7
Cheirogaleus major	47.69	-20.08	15.6	11.1	63	25.92	23.9	6.3	17.6	18.3	12.3	18.3
Cheirogaleus major	47.76	-19.92	17.8	11.2	64	26.07	26	8.6	17.4	20.6	14.4	20.6
Cheirogaleus major	47.95	-18.49	18.3	11.7	66	24.42	26.5	8.9	17.6	20.8	14.9	20.8
Cheirogaleus major	48.39	-14.33	20.7	12.5	73	15.84	28.7	11.6	17.1	22.1	18.4	22.1
Cheirogaleus major	48.47	-18.47	18.1	11.1	64	26.15	26.2	9.1	17.1	20.9	15.7	20.9
Cheirogaleus major	48.5	-18.85	20	10.8	63	25.9	28	11.1	16.9	22.8	17.7	22.8
Cheirogaleus major	48.97	-17.43	22.8	10	66	22.49	30	14.9	15.1	25.3	20.6	25.3
Cheirogaleus major	49.07	-12.48	25	8.8	71	12.33	30.7	18.4	12.3	26.1	23.8	26.1
Cheirogaleus major	49.43	-14.7	15.7	12.2	71	21.37	23.9	6.8	17.1	18	12.7	18
Cheirogaleus major	49.55	-14.31	21.4	11.4	70	20.57	29	12.8	16.2	23.6	19.3	23.6
Cheirogaleus major	49.73	-16.38	22.4	8.1	61	20.74	29	15.9	13.1	24.9	21.6	24.9
Cheirogaleus major	50.13	-15.52	21.3	8.6	62	20.95	28.2	14.5	13.7	23.7	20.2	23.7
Cheirogaleus medius	43.43	-22.8	24.2	12.6	63	26.08	33.1	13.2	19.9	27.3	20.7	27.3
Cheirogaleus medius	44.63	-20.04	26	13.1	63	22.57	34.1	13.6	20.5	27.8	22.6	28.1
Cheirogaleus medius	44.67	-24.83	24.4	14.6	64	30.99	34.6	12.1	22.5	27.8	21	27.8
Cheirogaleus medius	44.69	-22.78	23.4	16.6	66	26.58	34.7	9.8	24.9	25.9	20.4	26.1
Cheirogaleus medius	45.27	-16.33	26.5	10.9	69	14.54	32.8	17.1	15.7	27.4	24.2	27.7
Cheirogaleus medius	46.07	-16.19	26.9	11	70	13.15	33.7	18	15.7	27.7	24.9	28.1
Cheirogaleus medius	46.33	-24.83	23.8	14.9	67	29.03	33.7	11.6	22.1	27.1	20.5	27.1
Cheirogaleus medius	46.55	-24.52	22	13.3	65	27.15	31.1	10.9	20.2	25	18.9	25
Cheirogaleus medius	48.39	-14.33	20.7	12.5	73	15.84	28.7	11.6	17.1	22.1	18.4	22.1

Cheirogaleus medius	49.07	-12.93	25.8	9.9	70	12.06	32	18	14	26.6	24	26.8
Daubentonia madagascariensis	44.75	-18.74	25.4	12.6	65	18.66	33.8	14.5	19.3	26.8	22.9	27.1
Daubentonia madagascariensis	46.40	-24.50	22.7	14.1	65	28.15	32.4	10.9	21.5	25.9	19.7	25.9
Daubentonia madagascariensis	47.00	-22.00	17.4	10.5	61	24.79	25.4	8.3	17.1	19.9	14.5	19.9
Daubentonia madagascariensis	47.40	-21.20	17.9	10.5	61	25.73	26	8.8	17.2	20.6	16.2	20.6
Daubentonia madagascariensis	47.43	-20.68	17.2	11	63	25.73	25.3	7.9	17.4	19.9	13.8	19.9
Daubentonia madagascariensis	48.20	-14.85	26.3	11.8	70	15.03	34	17.2	16.8	27.3	23.9	27.5
Daubentonia madagascariensis	48.21	-14.30	26.1	10.6	73	12.18	32.4	17.9	14.5	26.9	24.2	27.1
Daubentonia madagascariensis	49.05	-17.65	25	9.1	71	12.19	30.7	18	12.7	26	23.2	26
Daubentonia madagascariensis	49.10	-12.50	25	8.8	71	12.33	30.7	18.4	12.3	26.1	23.8	26.1
Daubentonia madagascariensis	49.37	-12.78	25.2	9.5	73	12.47	31	18	13	26.3	24	26.3
Daubentonia madagascariensis	49.60	-14.40	19.9	11.7	70	21.46	27.9	11.2	16.7	22.2	17.8	22.2
Daubentonia madagascariensis	50.00	-15.67	23.5	7.9	60	20.08	30.1	17.1	13	25.5	22.5	25.8
Eulemur albifrons	48.05	-20.84	21.4	9.7	61	24.16	28.8	13.1	15.7	24	19.4	24.1
Eulemur albifrons	48.12	-21.81	22.9	7.8	56	22.9	29.3	15.6	13.7	25.7	20.9	25.7
Eulemur albifrons	48.77	-16.94	19.3	11.1	66	23.89	27.5	10.7	16.8	21.8	17.2	22
Eulemur albifrons	48.8	-16.28	18.8	11.4	66	23.55	27.2	10.1	17.1	21.2	16.5	21.3
Eulemur albifrons	48.97	-17.43	22.8	10	66	22.49	30	14.9	15.1	25.3	20.6	25.3
Eulemur albifrons	49.14	-15.41	20	11.7	69	22.28	28.3	11.4	16.9	22.3	16.8	22.3
Eulemur albifrons	49.15	-15.41	19.9	11.7	69	22.3	28.2	11.3	16.9	22.2	16.7	22.2
Eulemur albifrons	49.16	-15.49	19.6	11.6	68	22.55	27.9	11	16.9	21.9	16.3	21.9
Eulemur albifrons	49.16	-15.48	19.6	11.6	68	22.46	27.9	11	16.9	21.9	16.3	21.9
Eulemur albifrons	49.17	-17.88	23	8.8	60	23.16	30.2	15.7	14.5	25.8	20.6	25.8
Eulemur albifrons	49.17	-15.49	19.6	11.6	69	22.39	27.9	11.1	16.8	21.9	16.4	21.9
Eulemur albifrons	49.18	-17.83	22.3	8.9	58	24.05	29.9	14.8	15.1	25.2	19.8	25.2
Eulemur albifrons	49.28	-18.52	24.1	7.5	58	20.78	30.3	17.4	12.9	26.5	23.3	26.5
Eulemur albifrons	49.38	-15.68	20.8	10.5	67	22.3	28.6	13	15.6	23.2	18.6	23.2
Eulemur albifrons	49.42	-14.68	16.3	12.3	71	21.51	24.5	7.4	17.1	18.6	13.3	18.6
Eulemur albifrons	49.43	-14.77	18.5	12.2	71	21.93	26.6	9.5	17.1	20.8	15.3	20.8
Eulemur albifrons	49.43	-14.54	19.8	12.2	70	21.61	28	10.8	17.2	22.1	16.7	22.1
Eulemur albifrons	49.44	-15.4	21.4	10.6	67	22.36	29.1	13.4	15.7	23.7	19.1	23.7
Eulemur albifrons	49.44	-14.78	18.7	12.1	70	22.15	26.9	9.8	17.1	21.1	15.5	21.1
Eulemur albifrons	49.44	-14.74	16.8	12.2	71	21.69	25.1	8	17.1	19.1	13.7	19.1
Eulemur albifrons	49.44	-14.54	20.4	12	70	21.85	28.4	11.3	17.1	22.7	18.2	22.7
Eulemur albifrons	49.46	-14.75	18.9	12.1	71	22.3	27	10	17	21.2	15.7	21.2
Eulemur albifrons	49.51	-14.76	21.1	11.3	68	22.36	28.9	12.5	16.4	23.4	18.7	23.4
Eulemur albifrons	49.53	-15.44	22	10	66	21.98	29.4	14.4	15	24.4	19.7	24.4
Eulemur albifrons	49.53	-15.23	22.7	9.8	66	21.62	30	15.3	14.7	25.1	20.5	25.1
Eulemur albifrons	49.55	-15.29	21.9	10.2	67	21.88	29.4	14.2	15.2	24.3	19.6	24.3
Eulemur albifrons	49.57	-15.28	22.6	9.7	65	21.59	29.8	15.1	14.7	24.9	20.3	24.9
Eulemur albifrons	49.58	-15.04	21	10.8	67	22.51	28.7	12.8	15.9	23.4	18.6	23.4
Eulemur albifrons	49.61	-15.19	22.3	9.9	66	21.9	29.7	14.7	15	24.7	20	24.7
Eulemur albifrons	49.61	-14.43	19.9	11.7	70	21.46	27.9	11.2	16.7	22.2	17.8	22.2
Eulemur albifrons	49.62	-15.53	24.1	8.4	63	20.47	30.8	17.6	13.2	26.1	23.3	26.5
Eulemur albifrons	49.62	-14.44	19	11.8	70	21.36	27.1	10.3	16.8	21.3	16.9	21.3
Eulemur albifrons	49.73	-16.38	22.4	8.1	61	20.74	29	15.9	13.1	24.9	21.6	24.9
Eulemur albifrons	49.74	-14.44	17.8	11.7	70	21.34	25.8	9.2	16.6	20.1	15.7	20.1
Eulemur albifrons	49.78	-14.44	22.2	10.2	67	20.4	29.5	14.4	15.1	24.5	20	24.5
Eulemur albifrons	49.8	-14.43	20.8	10.8	68	20.94	28.4	12.6	15.8	23.1	18.5	23.1
Eulemur albifrons	49.96	-15.68	23.5	7.9	60	20.08	30.1	17.1	13	25.5	22.5	25.8
Eulemur albifrons	50.05	-15.27	21.2	9.1	64	21.36	28.3	14.1	14.2	23.6	20.1	23.6
Eulemur albifrons	50.07	-15.28	21.2	9	63	21.09	28.4	14.2	14.2	23.7	20.2	23.7

Eulemur albifrons	50.17	-14.27	24.7	8.1	63	17.02	30.9	18.1	12.8	26.7	23.9	26.7
Eulemur albifrons	50.18	-15.74	23.7	7.5	59	19.54	30.1	17.5	12.6	25.7	22.7	26
Eulemur albifrons	50.23	-15.78	24	7.3	59	19.33	30.2	17.9	12.3	25.9	23	26.3
Eulemur albifrons	50.23	-15.3	23.4	7.9	61	19.17	29.8	17	12.8	25.4	22.4	25.7
Eulemur albifrons	50.28	-15.78	24.2	7.2	58	19.22	30.4	18.1	12.3	26.1	23.2	26.5
Eulemur albifrons	50.29	-15.27	22.9	8	62	19.31	29.4	16.5	12.9	24.9	21.9	25.2
Eulemur albifrons	50.43	-15.4	24.1	7.4	60	18.54	30.2	18	12.2	26	23.1	26.3
Eulemur albocollaris	46.97	-22.22	18.3	10.7	62	24.95	26.4	9.2	17.2	20.9	15.4	20.9
Eulemur albocollaris	47	-22.23	19.7	10.7	62	25.35	27.6	10.4	17.2	22.4	16.6	22.4
Eulemur albocollaris	47.02	-22.22	20.2	10.7	61	25.34	28.1	10.8	17.3	22.8	17	22.8
Eulemur albocollaris	47.03	-22.2	19.7	10.6	61	25.27	27.7	10.5	17.2	22.4	16.6	22.4
Eulemur albocollaris	47.15	-22.82	21	10.5	62	25.23	28.5	11.8	16.7	23.6	17.9	23.8
Eulemur albocollaris	47.19	-22.8	20.9	10.4	62	25.14	28.4	11.8	16.6	23.5	19.3	23.6
Eulemur albocollaris	47.23	-22.44	21.6	10.1	61	24.9	29	12.5	16.5	24.2	19.9	24.3
Eulemur albocollaris	47.72	-23.03	23.4	8.7	56	23.5	30.5	15.2	15.3	26.2	21.5	26.2
Eulemur albocollaris	47.73	-23.03	23.4	8.7	56	23.5	30.5	15.2	15.3	26.2	21.5	26.2
Eulemur collaris	46.47	-23.35	17.7	11.7	65	24.14	26.2	8.3	17.9	20.3	14.2	20.3
Eulemur collaris	46.72	-24.56	15.4	10.4	66	21.99	22.8	7.2	15.6	17.9	12.6	17.9
Eulemur collaris	46.73	-24.57	17.2	10.8	66	22.73	24.8	8.6	16.2	19.7	14.4	19.7
Eulemur collaris	46.74	-24.6	19.4	11.5	65	24.38	27.5	10	17.5	22.2	16.6	22.2
Eulemur collaris	46.74	-24.58	18.3	11.2	66	23.58	26.1	9.2	16.9	20.9	15.4	20.9
Eulemur collaris	46.77	-24.93	23.1	11.3	63	25.24	31.1	13.3	17.8	25.9	21.2	26
Eulemur collaris	46.77	-24.63	21.7	12.1	65	25.8	30.1	11.5	18.6	24.6	18.6	24.6
Eulemur collaris	46.78	-25.08	22.5	10.7	62	24.72	30.3	13.3	17	25.4	20.6	25.4
Eulemur collaris	46.82	-24.68	23	11.8	63	25.73	31.4	12.8	18.6	25.9	21.4	26
Eulemur collaris	46.84	-24.73	20.1	11.2	64	24.31	28.1	10.8	17.3	22.9	17.2	22.9
Eulemur collaris	46.85	-24.76	21	11.3	64	24.71	29	11.4	17.6	23.8	18	23.8
Eulemur collaris	46.86	-24.73	21.4	11.4	64	25	29.5	11.8	17.7	24.3	18.4	24.3
Eulemur collaris	46.88	-24.9	23.1	10.2	61	24.23	30.7	14	16.7	25.9	21.3	25.9
Eulemur collaris	47	-24.5	23.1	11.2	62	25.32	31.2	13.2	18	25.8	21.4	25.9
Eulemur collaris	47.02	-24.97	22.8	8.8	58	22.94	29.7	14.7	15	25.5	22.4	25.5
Eulemur collaris	47.02	-22.15	17.4	10.5	61	24.79	25.4	8.3	17.1	19.9	14.5	19.9
Eulemur collaris	47.04	-22.16	16.3	10.4	61	24.38	24.3	7.5	16.8	18.8	13.5	18.8
Eulemur collaris	47.06	-23.66	19.8	11.3	63	25.92	27.8	9.9	17.9	22.6	16.7	22.6
Eulemur collaris	47.17	-24.6	23	10	60	24.03	30.5	13.9	16.6	25.7	21.3	25.7
Eulemur collaris	47.2	-24.58	22.2	10	60	24.03	29.7	13.2	16.5	24.9	20.5	24.9
Eulemur collaris	47.31	-24.23	23.1	10.4	61	24.17	30.6	13.8	16.8	25.6	21.4	25.8
Eulemur coronatus	49.06	-12.93	26	9.8	70	11.92	32.2	18.3	13.9	26.8	24.3	27
Eulemur coronatus	49.07	-12.92	25.8	9.8	70	11.99	32	18.1	13.9	26.6	24.1	26.8
Eulemur coronatus	49.14	-12.97	26.1	10	69	12.12	32.5	18.2	14.3	26.9	24.3	27.1
Eulemur coronatus	49.17	-12.52	20.4	9.6	73	13.97	26.4	13.4	13	21.8	19.1	21.8
Eulemur coronatus	49.24	-13.1	24.4	10.5	71	13.05	31	16.3	14.7	25.4	22.5	25.5
Eulemur coronatus	49.37	-12.78	25.2	9.5	73	12.47	31	18	13	26.3	24	26.3
Eulemur coronatus	49.38	-12.26	26.4	7.9	68	12.05	32	20.4	11.6	27.5	25.1	27.6
Eulemur coronatus	49.49	-12.75	25.9	9	70	12.38	31.6	18.9	12.7	27	24.7	27
Eulemur coronatus	49.54	-12.81	25.6	9.2	71	12.74	31.3	18.5	12.8	26.8	24.4	26.8
Eulemur coronatus	49.57	-13.25	22.5	10.3	72	15.08	28.9	14.7	14.2	23.9	20.3	23.9
Eulemur coronatus	49.62	-13.26	23.9	9.9	72	14.31	30	16.3	13.7	25.2	21.8	25.2
Eulemur coronatus	49.7	-13.11	24.5	9.4	72	13.9	30.3	17.3	13	25.8	23.1	25.8
Eulemur coronatus	49.91	-13.1	25.8	8.3	70	13.94	31.1	19.4	11.7	27.2	24.3	27.2
Eulemur coronatus	49.92	-13.25	24.3	8.8	70	14.93	30	17.6	12.4	25.8	22.8	25.8
Eulemur coronatus	50.01	-13.36	25.5	7.9	67	15.17	31	19.3	11.7	27.1	24.9	27.1

Eulemur coronatus	50.05	-14.1	24.1	8.7	65	17.36	30.4	17.2	13.2	26	23.3	26
Eulemur fulvus	46.47	-15.72	26.9	10.2	69	11.92	33.2	18.6	14.6	27.6	25.6	27.9
Eulemur fulvus	46.67	-15.47	26.9	10.8	68	13.53	33.5	17.7	15.8	27.7	24.8	28
Eulemur fulvus	46.69	-15.48	26.9	10.8	67	13.63	33.7	17.6	16.1	27.7	24.8	28.1
Eulemur fulvus	46.79	-16.34	26.3	12.8	69	13.65	34.9	16.5	18.4	26.9	24.7	27.7
Eulemur fulvus	46.82	-16.3	26.6	12.8	69	13.63	35.2	16.7	18.5	27.3	25	28
Eulemur fulvus	46.88	-22.57	21.3	11.2	63	25.66	29.3	11.6	17.7	23.8	18.2	24
Eulemur fulvus	46.95	-16.15	26.6	12.9	68	14.29	35.2	16.3	18.9	27.5	25	28
Eulemur fulvus	46.95	-16.12	26.4	12.9	68	14.45	35	16.1	18.9	27.3	24.2	27.8
Eulemur fulvus	46.96	-16.23	26.2	12.9	69	13.5	34.7	16.2	18.5	26.9	24.1	27.5
Eulemur fulvus	47.03	-16.86	27	12.4	70	12.67	35.4	17.8	17.6	27.5	25.1	28.4
Eulemur fulvus	47.12	-15.66	26.8	13.1	62	18.48	35.6	14.8	20.8	27.9	24	28.5
Eulemur fulvus	47.14	-16.23	26.3	13	69	13.61	34.9	16.3	18.6	27.1	24.2	27.6
Eulemur fulvus	47.15	-16.23	26.5	13	69	13.61	35.1	16.4	18.7	27.3	24.4	27.8
Eulemur fulvus	47.28	-18.17	17.4	12.3	67	21.37	25.8	7.5	18.3	19.4	14.2	19.4
Eulemur fulvus	47.3	-16.08	26.3	13.2	68	15.73	35	15.6	19.4	27.3	23.9	27.8
Eulemur fulvus	47.48	-15.5	26.6	13.1	63	18.37	35.7	15.2	20.5	27.8	23.8	28.3
Eulemur fulvus	47.68	-15.33	25.8	12.9	66	17.27	34.6	15.2	19.4	26.9	23.1	27.3
Eulemur fulvus	47.74	-15.16	26.1	12.5	68	15.73	34.3	16.1	18.2	27.2	23.7	27.5
Eulemur fulvus	47.76	-19.92	17.8	11.2	64	26.07	26	8.6	17.4	20.6	14.4	20.6
Eulemur fulvus	47.76	-19.92	17.8	11.2	64	26.07	26	8.6	17.4	20.6	14.4	20.6
Eulemur fulvus	47.77	-19.68	16.4	11.1	63	25.74	24.7	7.2	17.5	19	13	19
Eulemur fulvus	47.78	-14.79	25.9	11.3	71	11.25	33.2	17.3	15.9	26.5	24.1	26.8
Eulemur fulvus	47.83	-19.71	17.3	11.1	64	26.1	25.5	8.2	17.3	20.1	13.8	20.1
Eulemur fulvus	47.84	-19.71	17.4	11.1	64	26.01	25.6	8.3	17.3	20.1	13.9	20.1
Eulemur fulvus	47.92	-13.9	26.4	9.1	71	10.74	31.8	19.1	12.7	27.1	24.7	27.3
Eulemur fulvus	47.95	-18.41	17.9	11.7	66	24.29	26.1	8.5	17.6	20.4	14.5	20.4
Eulemur fulvus	48.2	-14.87	26.3	11.8	70	15.03	34	17.2	16.8	27.3	23.9	27.5
Eulemur fulvus	48.21	-14.88	25.7	11.9	70	15.24	33.5	16.6	16.9	26.8	23.3	27
Eulemur fulvus	48.21	-14.86	25.7	11.9	71	15.01	33.4	16.7	16.7	26.8	23.4	27
Eulemur fulvus	48.23	-19.67	21.2	10.6	65	24.45	29.1	12.8	16.3	24	19.1	24
Eulemur fulvus	48.23	-16.22	19.8	12.4	69	20.48	28.3	10.5	17.8	21.7	17.6	21.7
Eulemur fulvus	48.33	-19.73	21	10.2	63	24.53	28.6	12.6	16	23.7	18.8	23.7
Eulemur fulvus	48.37	-18.87	19.5	11.1	64	25.76	27.6	10.5	17.1	22.3	17.2	22.3
Eulemur fulvus	48.42	-14.02	17.5	12.5	73	16.85	25.7	8.6	17.1	19.1	15.1	19.1
Eulemur fulvus	48.42	-13.98	22	11.7	71	15.02	29.5	13.2	16.3	23.3	19.9	23.4
Eulemur fulvus	48.43	-19.18	20.8	10.5	64	24.55	28.6	12.4	16.2	23.6	18.6	23.6
Eulemur fulvus	48.43	-18.93	19	10.8	64	25.77	27.1	10.3	16.8	21.8	16.7	21.8
Eulemur fulvus	48.43	-18.79	19.5	11.1	64	26.34	27.7	10.4	17.3	22.3	17.2	22.3
Eulemur fulvus	48.43	-14	19.2	12.4	72	16.65	27.3	10.1	17.2	20.8	16.8	20.8
Eulemur fulvus	48.46	-18.97	18.7	10.6	63	25.94	26.7	10	16.7	21.5	16.4	21.5
Eulemur fulvus	48.47	-18.97	18.2	10.6	63	26.15	26.3	9.7	16.6	21.1	15.9	21.1
Eulemur fulvus	48.47	-18.7	20	11.1	63	26.62	28.2	10.7	17.5	22.8	17.7	22.9
Eulemur fulvus	48.5	-18.89	19.6	10.8	64	25.81	27.6	10.8	16.8	22.4	17.3	22.4
Eulemur fulvus	48.51	-14.33	19.2	13	72	17.37	27.7	9.8	17.9	20.8	16.6	20.8
Eulemur fulvus	48.53	-18.92	20.8	10.5	64	24.89	28.6	12.2	16.4	23.5	18.5	23.5
Eulemur fulvus	48.58	-18.2	19.1	11.2	65	25.32	27.2	10.1	17.1	21.7	16.7	21.7
Eulemur fulvus	48.6	-18.18	19.5	11.3	65	25.37	27.6	10.4	17.2	22.1	17.1	22.1
Eulemur fulvus	48.63	-17.5	20.3	11.7	67	23.68	28.6	11.2	17.4	22.8	17.1	22.8
Eulemur fulvus	48.65	-14.27	19.7	13.2	71	18.32	28.5	10	18.5	21.4	17.1	21.4
Eulemur fulvus	48.75	-18.08	19.8	10.8	65	25.2	27.6	11	16.6	22.5	17.3	22.5
Eulemur fulvus	48.77	-17.52	19.4	11.2	66	23.97	27.6	10.8	16.8	22	16.1	22

Eulemur fulvus	48.81	-18.7	22.7	9.4	62	22.94	29.9	14.9	15	25.3	20.5	25.3
Eulemur fulvus	48.81	-18.62	22.3	9.7	62	23.72	29.6	14.2	15.4	24.9	20	24.9
Eulemur fulvus	48.83	-13.86	23.2	11.6	70	15.96	30.6	14.2	16.4	24.6	20.9	24.6
Eulemur fulvus	48.84	-13.87	22.6	11.8	70	16.54	30.2	13.5	16.7	24.1	20.2	24.1
Eulemur fulvus	48.85	-13.87	23	11.7	70	16.03	30.5	14	16.5	24.4	20.7	24.5
Eulemur fulvus	48.88	-18.72	23.8	8.9	62	21.53	30.6	16.3	14.3	26.3	23.1	26.3
Eulemur fulvus	48.93	-18.8	23.7	8.6	61	21.41	30.3	16.3	14	26.1	22.9	26.1
Eulemur fulvus	48.95	-14.21	18.6	13.4	71	20.41	27.6	8.8	18.8	20.6	15.7	20.6
Eulemur fulvus	48.97	-18.73	24.1	8.5	61	21.21	30.6	16.8	13.8	26.5	23.4	26.5
Eulemur fulvus	48.98	-17.73	22	9.9	63	23.91	29.7	14	15.7	24.8	19.7	24.8
Eulemur fulvus	49	-18.37	22.8	9.1	61	23.18	29.8	15.1	14.7	25.5	20.5	25.5
Eulemur fulvus	49	-17.69	21.6	9.9	62	23.96	29.3	13.5	15.8	24.4	19.2	24.4
Eulemur fulvus	49.03	-17.3	22.8	9.8	65	22.12	30	15.1	14.9	25.4	20.7	25.4
Eulemur fulvus	49.09	-18.05	22.5	9.1	60	23.97	30	14.9	15.1	25.3	20	25.3
Eulemur macaco	47.76	-14.38	25.9	10.1	73	9.4	31.9	18.2	13.7	26.4	24.4	26.7
Eulemur macaco	47.85	-14.49	26.2	10.3	73	9.22	32.5	18.4	14.1	26.6	24.7	26.9
Eulemur macaco	47.92	-13.6	26.5	8.2	70	11.51	31.5	19.9	11.6	27.4	24.7	27.5
Eulemur macaco	47.94	-14.39	26.4	10.2	73	9.81	32.6	18.7	13.9	27	24.9	27.2
Eulemur macaco	47.97	-14.03	26.4	9.5	72	10.71	32	18.9	13.1	27.1	24.7	27.3
Eulemur macaco	47.98	-14.22	26.6	9.9	73	10.35	32.5	19	13.5	27.3	25	27.5
Eulemur macaco	48	-14.53	26.8	10.6	72	10.6	33.3	18.7	14.6	27.4	25.1	27.6
Eulemur macaco	48.17	-14.27	26.3	10.4	72	11.86	32.6	18.2	14.4	27.1	24.5	27.3
Eulemur macaco	48.2	-14.2	25.3	10.6	72	12.23	31.6	17	14.6	26.2	23.4	26.3
Eulemur macaco	48.23	-14.29	26.1	10.6	73	12.18	32.4	17.9	14.5	26.9	24.2	27.1
Eulemur macaco	48.26	-14.61	24.7	11.7	72	14.07	32	15.9	16.1	25.7	22.5	25.8
Eulemur macaco	48.27	-14.54	25.3	11.4	73	13.44	32.3	16.7	15.6	26.3	23.2	26.4
Eulemur macaco	48.32	-13.41	24.8	8.9	70	12.94	30.5	17.8	12.7	25.9	22.9	26
Eulemur macaco	48.34	-13.39	26.2	7.8	67	12.77	31.3	19.8	11.5	27.3	24.3	27.4
Eulemur macaco	48.36	-13.66	26.1	9.4	69	12.78	31.7	18.1	13.6	27.1	24.2	27.2
Eulemur macaco	48.36	-13.55	25.9	9	68	12.81	31.4	18.3	13.1	26.9	23.9	27
Eulemur macaco	48.41	-14.28	20.3	12.6	72	16.19	28.4	11.1	17.3	21.8	17.9	21.8
Eulemur macaco	48.42	-14.02	17.5	12.5	73	16.85	25.7	8.6	17.1	19.1	15.1	19.1
Eulemur macaco	48.42	-13.98	22	11.7	71	15.02	29.5	13.2	16.3	23.3	19.9	23.4
Eulemur macaco	48.43	-14	19.2	12.4	72	16.65	27.3	10.1	17.2	20.8	16.8	20.8
Eulemur macaco	48.43	-13.96	23.2	11.3	71	14.38	30.3	14.6	15.7	24.4	21.1	24.5
Eulemur macaco	48.49	-13.71	25.6	10.1	69	13.09	31.7	17.2	14.5	26.7	23.7	26.8
Eulemur macaco	48.53	-13.42	26.4	8.9	67	12.84	32	18.9	13.1	27.4	24.4	27.4
Eulemur macaco	48.56	-13.45	25	9.6	69	13.39	31.1	17.2	13.9	26.1	23	26.2
Eulemur macaco	48.57	-13.45	25.6	9.5	68	13.07	31.6	17.8	13.8	26.6	23.6	26.7
Eulemur macaco	48.73	-13.79	23.6	11.3	71	15.17	30.7	14.8	15.9	24.9	21.4	24.9
Eulemur macaco	48.8	-13.43	26.5	9.9	68	12.87	32.8	18.4	14.4	27.4	24.7	27.5
Eulemur macaco	48.83	-13.86	23.2	11.6	70	15.96	30.6	14.2	16.4	24.6	20.9	24.6
Eulemur macaco	48.83	-13.33	26.6	9.9	68	12.7	33	18.5	14.5	27.5	24.8	27.7
Eulemur macaco	48.84	-13.87	22.6	11.8	70	16.54	30.2	13.5	16.7	24.1	20.2	24.1
Eulemur rubriventer	47.44	-20.68	17.1	11	63	25.73	25.2	7.8	17.4	19.8	13.7	19.8
Eulemur rubriventer	47.47	-20.69	17.6	11	63	25.69	25.7	8.4	17.3	20.4	15.8	20.4
Eulemur rubriventer	47.62	-20.39	16.9	11	63	26.04	25.1	7.7	17.4	19.7	13.6	19.7
Eulemur rubriventer	47.63	-20.39	17	11	63	25.9	25.1	7.8	17.3	19.7	13.6	19.7
Eulemur rubriventer	47.64	-20.39	17	10.9	62	26.05	25.2	7.8	17.4	19.7	13.6	19.7
Eulemur rubriventer	47.67	-20.06	15.9	11.2	63	25.99	24.2	6.5	17.7	18.6	12.5	18.6
Eulemur rubriventer	47.67	-20.06	15.9	11.2	63	25.99	24.2	6.5	17.7	18.6	12.5	18.6
Eulemur rubriventer	47.69	-20.08	15.6	11.1	63	25.92	23.9	6.3	17.6	18.3	12.3	18.3

Eulemur rubriventer	47.76	-19.92	17.8	11.2	64	26.07	26	8.6	17.4	20.6	14.4	20.6
Eulemur rufus	43.43	-22.8	24.2	12.6	63	26.08	33.1	13.2	19.9	27.3	20.7	27.3
Eulemur rufus	43.79	-21.3	25.1	12.5	63	25.6	33.1	13.5	19.6	27.9	21.5	27.9
Eulemur rufus	43.92	-21.67	25.1	13.4	65	25.81	33.5	13	20.5	27.9	21.4	27.9
Eulemur rufus	43.94	-23.19	23.6	14.1	65	27.18	33	11.5	21.5	26.6	19.7	26.6
Eulemur rufus	44.01	-20.75	25.4	12.1	62	24.96	33.1	13.8	19.3	28	21.8	28
Eulemur rufus	44.03	-18.07	25.7	9	62	18.4	32.1	17.6	14.5	27.4	22.9	27.4
Eulemur rufus	44.07	-20.81	25.3	12.4	63	24.97	33.2	13.6	19.6	27.9	21.7	27.9
Eulemur rufus	44.07	-17.79	25.8	9.7	64	17.97	32.3	17.2	15.1	27.5	23.1	27.5
Eulemur rufus	44.12	-21.72	25.3	14	65	25.99	34.2	12.8	21.4	28	21.5	28
Eulemur rufus	44.13	-21.83	25.4	14.1	64	26.21	34.5	12.8	21.7	28.1	21.6	28.1
Eulemur rufus	44.13	-21.77	25.4	14	64	26.26	34.5	12.9	21.6	28.1	21.6	28.1
Eulemur rufus	44.13	-21.71	25.3	14	65	25.95	34.2	12.8	21.4	28	21.5	28
Eulemur rufus	44.13	-21.7	25.3	13.9	64	26.24	34.2	12.7	21.5	28	21.4	28
Eulemur rufus	44.17	-22.64	19.1	13.9	68	23.44	28.3	7.9	20.4	21.6	15.7	21.6
Eulemur rufus	44.18	-22.1	24.6	14.8	66	25.82	34.2	11.9	22.3	27.2	20.8	27.2
Eulemur rufus	44.25	-21.63	24.8	14.1	64	25.85	33.9	12.2	21.7	27.3	20.9	27.3
Eulemur rufus	44.28	-21.27	25	13.6	63	25.8	33.8	12.4	21.4	27.6	21.2	27.6
Eulemur rufus	44.35	-20.58	25.5	12.7	62	24.62	33.5	13.3	20.2	27.7	21.9	27.9
Eulemur rufus	44.35	-20.43	25.4	12.2	62	24.35	33.3	13.7	19.6	27.7	21.9	27.9
Eulemur rufus	44.38	-20.45	25.5	12.4	62	24.2	33.4	13.5	19.9	27.7	22	27.9
Eulemur rufus	44.43	-20.17	25.6	12.2	62	23.5	33.3	13.9	19.4	27.8	22.2	28
Eulemur rufus	44.48	-21.33	24.6	14	63	25.68	33.9	12	21.9	27.1	20.8	27.1
Eulemur rufus	44.5	-22.28	24.7	15.7	66	25.66	35.2	11.5	23.7	27.2	21.8	27.3
Eulemur rufus	44.53	-19.23	26.2	12.2	64	20.08	34	15	19	27.9	23.1	28
Eulemur rufus	44.55	-20.12	25.9	12.8	63	23.06	33.9	13.7	20.2	27.9	22.5	28.2
Eulemur rufus	44.6	-19.97	26.1	12.9	64	22.32	34.1	14	20.1	28.2	22.7	28.2
Eulemur rufus	44.62	-18.65	25.9	12	65	18.78	33.8	15.4	18.4	27.4	23.4	27.6
Eulemur rufus	44.65	-20.07	26	13.3	64	22.62	34.2	13.5	20.7	27.9	22.7	28.1
Eulemur rufus	44.66	-20.07	26	13.3	63	22.52	34.3	13.5	20.8	27.8	22.7	28.1
Eulemur rufus	44.67	-22.76	23.8	16.8	66	26.74	35.3	10.1	25.2	26.3	20.9	26.5
Eulemur rufus	44.68	-20.08	26	13.4	64	22.48	34.4	13.5	20.9	27.8	22.7	28.1
Eulemur rufus	44.68	-16.87	26.4	11.7	67	15.35	33.7	16.3	17.4	27.4	24	27.8
Eulemur rufus	44.7	-21.3	24.5	14.3	63	25.93	34.1	11.7	22.4	26.9	20.6	27
Eulemur rufus	44.7	-20.58	25.8	14.1	62	24.04	35.1	12.7	22.4	28.1	22.3	28.1
Eulemur rufus	44.72	-16.88	25.7	11.9	68	15.07	33	15.7	17.3	26.7	23.4	27
Eulemur rufus	44.73	-22.82	21.9	15.5	66	25.28	32.3	9.1	23.2	24.3	18.1	24.4
Eulemur rufus	44.73	-16.94	26.1	11.9	68	15.35	33.4	16	17.4	27.1	23.7	27.4
Eulemur rufus	44.73	-16.3	26.4	10.8	68	14.47	32.8	17.1	15.7	27.3	24.2	27.7
Eulemur rufus	44.75	-18.74	25.4	12.6	65	18.66	33.8	14.5	19.3	26.8	22.9	27.1
Eulemur rufus	44.8	-20.42	26.1	14.4	62	23.59	35.8	12.8	23	28.3	22.7	28.3
Eulemur rufus	44.81	-19.13	26.2	13	64	19.78	34.8	14.6	20.2	27.7	23.1	28
Eulemur rufus	44.83	-22.68	22.8	15.8	66	25.06	33.5	9.9	23.6	25.2	19.9	25.4
Eulemur rufus	44.83	-19.97	26.2	13.7	63	22.03	35	13.5	21.5	28.1	22.8	28.2
Eulemur rufus	44.83	-19	25	13	65	19.12	33.7	13.7	20	26.5	22.1	26.8
Eulemur rufus	44.84	-22.46	22.4	15.2	67	23.98	32.5	9.9	22.6	24.7	18.7	24.8
Eulemur rufus	44.9	-21	25.4	14.4	63	25.68	35	12.3	22.7	27.7	21.6	27.8
Eulemur rufus	45.05	-19.75	26.8	13.9	64	21.05	35.8	14.1	21.7	28.5	23.5	28.7
Eulemur rufus	45.09	-22.17	24.6	15.1	64	25.94	34.9	11.6	23.3	27	20.7	27.1
Eulemur rufus	45.1	-19.12	26.7	13.7	64	19.45	35.8	14.5	21.3	28	23.6	28.5
Eulemur rufus	45.13	-22.58	22.8	14.8	66	23.67	32.6	10.5	22.1	25.1	19.2	25.1
Eulemur rufus	45.21	-16.28	26.5	10.7	69	14.35	32.7	17.3	15.4	27.5	24.3	27.7

Eulemur rufus	45.26	-22.44	22.9	14.4	66	23.51	32.4	10.8	21.6	25.2	19.4	25.2
Eulemur rufus	45.26	-22.37	23.1	14.4	66	23.94	32.8	11	21.8	25.4	19.5	25.5
Eulemur rufus	45.29	-22.32	23.5	14.5	65	24.42	33.3	11.2	22.1	25.8	19.8	25.9
Eulemur rufus	45.34	-16.1	26.7	10.1	67	14.47	32.7	17.7	15	27.7	24.5	27.9
Eulemur rufus	45.36	-16.43	26.1	11.2	69	14.51	32.7	16.5	16.2	27	23.8	27.3
Eulemur rufus	45.38	-22.48	21.8	13.6	67	22.18	30.8	10.6	20.2	24.1	18.6	24.1
Eulemur rufus	45.55	-21.45	24.7	14.2	62	26.83	34.8	12.1	22.7	27.1	20.7	27.3
Eulemur rufus	45.57	-21.43	25	14.2	62	26.96	35.1	12.4	22.7	27.5	21	27.7
Eulemur rufus	45.78	-16.98	25.4	12.6	69	14.57	33.4	15.2	18.2	26.2	23.5	26.7
Eulemur rufus	45.83	-16	26.7	10.2	70	12.69	32.7	18.3	14.4	27.4	24.7	27.7
Eulemur rufus	45.88	-16.22	26.8	10.8	70	13.24	33.3	17.9	15.4	27.6	24.7	27.9
Eulemur rufus	45.9	-16.08	26.9	10.5	71	12.76	33	18.3	14.7	27.6	24.9	27.9
Eulemur rufus	45.9	-16.05	26.9	10.4	70	12.7	33.1	18.4	14.7	27.7	24.9	28
Eulemur rufus	45.9	-16.03	26.9	10.3	71	12.61	32.9	18.5	14.4	27.7	24.9	27.9
Eulemur rufus	45.92	-16.05	26.9	10.4	71	12.69	33	18.4	14.6	27.6	24.9	27.9
Eulemur rufus	45.93	-17.08	26.2	12.8	69	14.67	34.4	16	18.4	27	24.3	27.6
Eulemur rufus	45.93	-16.1	26.9	10.5	70	12.74	33.2	18.3	14.9	27.7	24.9	28
Eulemur rufus	45.93	-16.08	26.9	10.5	70	12.77	33.2	18.4	14.8	27.6	24.9	28
Eulemur rufus	45.98	-17.07	26.6	12.8	69	14.57	34.8	16.5	18.3	27.3	24.7	28
Eulemur rufus	45.99	-17.07	26.8	12.7	69	14.6	35	16.7	18.3	27.6	24.9	28.2
Eulemur rufus	46.08	-16.52	26.6	11.9	70	13.61	34.1	17.1	17	27.3	24.9	27.9
Eulemur rufus	46.13	-16.25	26.9	11.3	70	13.17	33.9	17.8	16.1	27.6	24.8	28.1
Eulemur rufus	46.22	-15.72	26.7	9.5	73	10.74	32.3	19.3	13	27.3	25	27.6
Eulemur rufus	46.4	-16.55	26.9	12.3	69	13.88	35	17.3	17.7	27.5	25.2	28.4
Eulemur rufus	46.48	-16.38	26.7	12.3	68	14.06	34.9	17	17.9	27.4	25	28.2
Eulemur rufus	46.85	-22.21	17.1	10.8	62	24.51	25.3	8	17.3	19.6	14.3	19.6
Eulemur rufus	46.9	-22.42	18.9	10.9	61	25.18	27.1	9.5	17.6	21.5	16	21.5
Eulemur rufus	46.94	-22.43	18.9	10.7	61	25.06	26.9	9.6	17.3	21.5	16	21.5
Eulemur rufus	46.96	-22.5	16.1	10.5	62	24.1	24.1	7.4	16.7	18.7	13.4	18.7
Eulemur rufus	46.96	-22.47	19.8	10.8	62	25.21	27.7	10.5	17.2	22.5	16.7	22.5
Eulemur rufus	46.97	-22.48	19.3	10.7	62	25.24	27.2	10	17.2	21.9	16.2	21.9
Eulemur rufus	46.97	-22.38	20.9	10.8	62	25.47	28.7	11.4	17.3	23.4	17.7	23.6
Eulemur rufus	46.97	-22.22	18.3	10.7	62	24.95	26.4	9.2	17.2	20.9	15.4	20.9
Eulemur rufus	47.17	-20.78	15.3	11.2	62	25.18	23.7	5.8	17.9	17.8	12.1	17.8
Eulemur rufus	47.22	-21.2	16.9	10.7	60	25.52	25.3	7.7	17.6	19.5	15.3	19.5
Eulemur rufus	47.33	-21.6	18.4	10.2	60	25.49	26.3	9.4	16.9	21	15.1	21
Eulemur rufus	47.34	-21.83	19	10.1	61	25.16	26.5	10.1	16.4	21.6	15.8	21.6
Eulemur rufus	47.35	-21.78	18.7	10.1	60	25.16	26.4	9.8	16.6	21.3	15.5	21.3
Eulemur rufus	47.36	-21.82	19	10.1	61	25.12	26.6	10.1	16.5	21.6	17.3	21.6
Eulemur rufus	47.41	-21.74	19	10	60	25.26	26.6	10.2	16.4	21.7	17.3	21.7
Eulemur rufus	47.41	-21.51	18.4	10.2	61	25.37	26.2	9.6	16.6	21	16.6	21
Eulemur rufus	47.43	-21.48	20.6	10.6	61	25.4	28.5	11.3	17.2	23.2	18.9	23.3
Eulemur rufus	47.43	-21.29	18.5	10.4	61	25.57	26.5	9.5	17	21.2	16.8	21.2
Eulemur rufus	47.43	-21.29	18.5	10.4	61	25.57	26.5	9.5	17	21.2	16.8	21.2
Eulemur rufus	47.43	-20.69	17.2	11	63	25.73	25.3	7.9	17.4	19.9	13.8	19.9
Eulemur rufus	47.44	-20.69	17.2	11	63	25.79	25.4	8	17.4	19.9	13.9	19.9
Eulemur rufus	47.45	-21.51	20.7	10.5	61	25.51	28.6	11.4	17.2	23.3	18.9	23.4
Eulemur rufus	47.46	-21.3	19.7	10.6	61	25.45	27.7	10.5	17.2	22.3	18	22.4
Eulemur rufus	47.46	-21.2	18	10.4	61	25.77	26	9	17	20.7	16.2	20.7
Eulemur rufus	47.47	-21	18	10.7	62	25.72	26.1	9	17.1	20.6	16.3	20.7
Eulemur rufus	47.47	-20.69	17.6	11	63	25.69	25.7	8.4	17.3	20.4	15.8	20.4
Eulemur rufus	47.49	-21.59	18.9	10.1	61	25.35	26.6	10.1	16.5	21.6	17.2	21.6

Eulemur rufus	47.49	-21.54	20.2	10.3	60	25.35	28	11.1	16.9	22.8	18.5	23
Eulemur rufus	47.49	-21.39	20.3	10.6	61	25.5	28.3	11.1	17.2	22.9	18.5	23.1
Eulemur rufus	47.52	-21.34	20.3	10.6	60	25.65	28.4	11	17.4	22.9	18.6	23.1
Eulemur rufus	47.53	-21.25	20.5	10.7	61	25.61	28.6	11.2	17.4	23.1	18.7	23.2
Eulemur rufus	47.53	-21.24	20.2	10.7	61	25.71	28.2	10.9	17.3	22.8	18.4	23
Eulemur rufus	47.54	-21.26	20.4	10.7	61	25.57	28.4	11.1	17.3	23	18.6	23.2
Eulemur rufus	47.54	-21.24	20.1	10.7	61	25.71	28.1	10.8	17.3	22.7	18.3	22.9
Eulemur rufus	47.54	-21.23	20.7	10.8	61	25.51	28.8	11.3	17.5	23.3	18.9	23.4
Eulemur rufus	47.54	-21.12	18	10.4	61	25.52	25.9	9.1	16.8	20.5	16.1	20.7
Eulemur rufus	47.59	-20.88	20.7	10.9	62	25.34	28.8	11.4	17.4	23.3	18.9	23.4
Eulemur rufus	47.69	-20.29	17.2	11	63	26.25	25.4	8	17.4	20	13.8	20
Eulemur rufus	47.7	-21.37	20.6	10.3	61	25.06	28.3	11.6	16.7	23.2	18.7	23.3
Eulemur rufus	47.78	-22.65	23.5	8.2	55	23.11	30.4	15.6	14.8	26.3	21.6	26.3
Eulemur rufus	47.83	-22.35	23.3	7.9	56	22.87	29.8	15.8	14	26.1	21.4	26.1
Eulemur rufus	48.02	-22.15	23	7.2	54	22.62	29	15.9	13.1	25.7	21	25.7
Eulemur rufus	48.43	-19.18	20.8	10.5	64	24.55	28.6	12.4	16.2	23.6	18.6	23.6
Eulemur rufus	48.43	-18.93	19	10.8	64	25.77	27.1	10.3	16.8	21.8	16.7	21.8
Eulemur rufus	48.75	-18.08	19.8	10.8	65	25.2	27.6	11	16.6	22.5	17.3	22.5
Eulemur sanfordi	49.03	-12.63	25	9.1	71	12.19	30.7	18	12.7	26	23.2	26
Eulemur sanfordi	49.06	-12.93	26	9.8	70	11.92	32.2	18.3	13.9	26.8	24.3	27
Eulemur sanfordi	49.07	-12.92	25.8	9.8	70	11.99	32	18.1	13.9	26.6	24.1	26.8
Eulemur sanfordi	49.11	-12.91	25.9	9.8	70	12.05	32.2	18.2	14	26.8	24.2	26.9
Eulemur sanfordi	49.14	-12.97	26.1	10	69	12.12	32.5	18.2	14.3	26.9	24.3	27.1
Eulemur sanfordi	49.17	-12.52	20.4	9.6	73	13.97	26.4	13.4	13	21.8	19.1	21.8
Eulemur sanfordi	49.24	-13.1	24.4	10.5	71	13.05	31	16.3	14.7	25.4	22.5	25.5
Eulemur sanfordi	49.37	-12.78	25.2	9.5	73	12.47	31	18	13	26.3	24	26.3
Eulemur sanfordi	49.53	-12.81	25.3	9.2	71	12.72	31	18.2	12.8	26.5	24.1	26.5
Eulemur sanfordi	49.57	-13.25	22.5	10.3	72	15.08	28.9	14.7	14.2	23.9	20.3	23.9
Eulemur sanfordi	49.62	-13.26	23.9	9.9	72	14.31	30	16.3	13.7	25.2	21.8	25.2
Eulemur sanfordi	49.62	-13.26	23.9	9.9	72	14.31	30	16.3	13.7	25.2	21.8	25.2
Hapalemur griseus	46.88	-22.57	21.3	11.2	63	25.66	29.3	11.6	17.7	23.8	18.2	24
Hapalemur griseus	47.33	-18.18	20	12.7	67	21.93	28.6	9.8	18.8	22	16.7	22
Hapalemur griseus	47.47	-21.37	20.4	10.7	61	25.58	28.4	11.1	17.3	23	18.6	23.1
Hapalemur griseus	47.47	-20.69	17.6	11	63	25.69	25.7	8.4	17.3	20.4	15.8	20.4
Hapalemur griseus	47.63	-20.39	17	11	63	25.9	25.1	7.8	17.3	19.7	13.6	19.7
Hapalemur griseus	47.65	-20.39	17	11	63	25.94	25.2	7.9	17.3	19.8	13.7	19.8
Hapalemur griseus	47.67	-20.38	16.4	10.9	63	25.98	24.5	7.2	17.3	19.1	13	19.1
Hapalemur griseus	47.67	-20.06	15.9	11.2	63	25.99	24.2	6.5	17.7	18.6	12.5	18.6
Hapalemur griseus	47.69	-20.08	15.6	11.1	63	25.92	23.9	6.3	17.6	18.3	12.3	18.3
Hapalemur griseus	47.95	-18.49	18.3	11.7	66	24.42	26.5	8.9	17.6	20.8	14.9	20.8
Hapalemur griseus	48.47	-18.47	18.1	11.1	64	26.15	26.2	9.1	17.1	20.9	15.7	20.9
Hapalemur griseus	48.5	-18.85	20	10.8	63	25.9	28	11.1	16.9	22.8	17.7	22.8
Hapalemur griseus	48.97	-17.43	22.8	10	66	22.49	30	14.9	15.1	25.3	20.6	25.3
Hapalemur griseus	49.43	-14.7	15.7	12.2	71	21.37	23.9	6.8	17.1	18	12.7	18
Hapalemur griseus	49.55	-14.31	21.4	11.4	70	20.57	29	12.8	16.2	23.6	19.3	23.6
Hapalemur griseus	49.73	-16.38	22.4	8.1	61	20.74	29	15.9	13.1	24.9	21.6	24.9
Hapalemur griseus	50.13	-15.52	21.3	8.6	62	20.95	28.2	14.5	13.7	23.7	20.2	23.7
Hapalemur simus	46.88	-22.57	21.3	11.2	63	25.66	29.3	11.6	17.7	23.8	18.2	24
Hapalemur simus	47	-22.23	19.7	10.7	62	25.35	27.6	10.4	17.2	22.4	16.6	22.4
Hapalemur simus	47.03	-22.2	19.7	10.6	61	25.27	27.7	10.5	17.2	22.4	16.6	22.4
Hapalemur simus	47.03	-22.15	17.4	10.5	62	24.59	25.4	8.5	16.9	19.9	14.6	19.9
Hapalemur simus	47.22	-22.43	21	10.2	61	25.12	28.4	11.9	16.5	23.7	19.4	23.8

Hapalemur simus	47.42	-20.25	16.6	11.5	63	26.01	25	6.9	18.1	19.3	12.8	19.3
Hapalemur simus	47.47	-21.37	20.4	10.7	61	25.58	28.4	11.1	17.3	23	18.6	23.1
Indh indri	48.81	-18.7	22.7	9.4	62	22.94	29.9	14.9	15	25.3	20.5	25.3
Indh indri	48.88	-18.2	17.9	10.3	64	25.29	25.6	9.6	16	20.6	15.4	20.6
Indri indri	47.95	-18.41	17.9	11.7	66	24.29	26.1	8.5	17.6	20.4	14.5	20.4
Indri indri	48.2	-19.43	20.9	11	65	24.91	29.1	12.2	16.9	23.8	18.6	23.8
Indri indri	48.34	-19.19	19.1	10.7	64	25.69	27.2	10.6	16.6	22	16.9	22
Indri indri	48.37	-18.87	19.5	11.1	64	25.76	27.6	10.5	17.1	22.3	17.2	22.3
Indri indri	48.41	-18.48	19.8	11.4	64	26.42	28.1	10.5	17.6	22.5	17.3	22.6
Indri indri	48.43	-19.18	20.8	10.5	64	24.55	28.6	12.4	16.2	23.6	18.6	23.6
Indri indri	48.43	-18.93	19	10.8	64	25.77	27.1	10.3	16.8	21.8	16.7	21.8
Indri indri	48.43	-18.93	19	10.8	64	25.77	27.1	10.3	16.8	21.8	16.7	21.8
Indri indri	48.43	-18.79	19.5	11.1	64	26.34	27.7	10.4	17.3	22.3	17.2	22.3
Indri indri	48.45	-18.98	19.1	10.7	64	25.65	27.1	10.5	16.6	21.9	16.8	21.9
Indri indri	48.46	-18.97	18.7	10.6	63	25.94	26.7	10	16.7	21.5	16.4	21.5
Indri indri	48.46	-18.88	18.9	10.8	63	26.22	27	10	17	21.8	16.6	21.8
Indri indri	48.47	-18.97	18.2	10.6	63	26.15	26.3	9.7	16.6	21.1	15.9	21.1
Indri indri	48.47	-18.7	20	11.1	63	26.62	28.2	10.7	17.5	22.8	17.7	22.9
Indri indri	48.5	-18.89	19.6	10.8	64	25.81	27.6	10.8	16.8	22.4	17.3	22.4
Indri indri	48.52	-18.93	20.7	10.6	64	24.98	28.5	12.1	16.4	23.5	18.5	23.5
Indri indri	48.58	-18.2	19.1	11.2	65	25.32	27.2	10.1	17.1	21.7	16.7	21.7
Indri indri	48.6	-18.18	19.5	11.3	65	25.37	27.6	10.4	17.2	22.1	17.1	22.1
Indri indri	48.76	-17.68	19.4	11	66	24.19	27.4	10.8	16.6	21.9	16	21.9
Indri indri	48.77	-17.52	19.4	11.2	66	23.97	27.6	10.8	16.8	22	16.1	22
Indri indri	48.77	-16.94	19.3	11.1	66	23.89	27.5	10.7	16.8	21.8	17.2	22
Indri indri	48.78	-18.41	19.7	10.4	63	25.67	27.5	11.2	16.3	22.5	17.2	22.5
Indri indri	48.8	-16.28	18.8	11.4	66	23.55	27.2	10.1	17.1	21.2	16.5	21.3
Indri indri	48.88	-18.72	23.8	8.9	62	21.53	30.6	16.3	14.3	26.3	23.1	26.3
Indri indri	48.93	-18.8	23.7	8.6	61	21.41	30.3	16.3	14	26.1	22.9	26.1
Indri indri	48.97	-18.73	24.1	8.5	61	21.21	30.6	16.8	13.8	26.5	23.4	26.5
Indri indri	48.97	-18.53	22.1	9.2	61	23.5	29.2	14.3	14.9	24.8	19.7	24.8
Indri indri	48.97	-16.95	19.7	10.5	65	23.98	27.6	11.6	16	22.4	17.4	22.4
Indri indri	48.98	-17.73	22	9.9	63	23.91	29.7	14	15.7	24.8	19.7	24.8
Indri indri	49	-18.37	22.8	9.1	61	23.18	29.8	15.1	14.7	25.5	20.5	25.5
Indri indri	49	-17.69	21.6	9.9	62	23.96	29.3	13.5	15.8	24.4	19.2	24.4
Indri indri	49.03	-17.3	22.8	9.8	65	22.12	30	15.1	14.9	25.4	20.7	25.4
Indri indri	49.09	-18.05	22.5	9.1	60	23.97	30	14.9	15.1	25.3	20	25.3
Indri indri	49.14	-15.41	20	11.7	69	22.28	28.3	11.4	16.9	22.3	16.8	22.3
Indri indri	49.15	-15.41	19.9	11.7	69	22.3	28.2	11.3	16.9	22.2	16.7	22.2
Indri indri	49.16	-15.49	19.6	11.6	68	22.55	27.9	11	16.9	21.9	16.3	21.9
Indri indri	49.16	-15.48	19.6	11.6	68	22.46	27.9	11	16.9	21.9	16.3	21.9
Indri indri	49.17	-17.88	23	8.8	60	23.16	30.2	15.7	14.5	25.8	20.6	25.8
Indri indri	49.17	-15.49	19.6	11.6	69	22.39	27.9	11.1	16.8	21.9	16.4	21.9
Indri indri	49.18	-17.83	22.3	8.9	58	24.05	29.9	14.8	15.1	25.2	19.8	25.2
Indri indri	49.22	-17.9	22.7	8.6	58	23.84	30.2	15.4	14.8	25.6	20.1	25.6
Indri indri	49.22	-17.9	22.7	8.6	58	23.84	30.2	15.4	14.8	25.6	20.1	25.6
Indri indri	49.28	-18.52	24.1	7.5	58	20.78	30.3	17.4	12.9	26.5	23.3	26.5
Indri indri	49.38	-15.68	20.8	10.5	67	22.3	28.6	13	15.6	23.2	18.6	23.2
Indri indri	49.42	-14.68	16.3	12.3	71	21.51	24.5	7.4	17.1	18.6	13.3	18.6
Indri indri	49.44	-15.4	21.4	10.6	67	22.36	29.1	13.4	15.7	23.7	19.1	23.7
Indri indri	49.44	-14.78	18.7	12.1	70	22.15	26.9	9.8	17.1	21.1	15.5	21.1
Indri indri	49.51	-14.76	21.1	11.3	68	22.36	28.9	12.5	16.4	23.4	18.7	23.4

Indri indri	49.53	-15.44	22	10	66	21.98	29.4	14.4	15	24.4	19.7	24.4
Indri indri	49.53	-15.23	22.7	9.8	66	21.62	30	15.3	14.7	25.1	20.5	25.1
Indri indri	49.55	-15.29	21.9	10.2	67	21.88	29.4	14.2	15.2	24.3	19.6	24.3
Indri indri	49.57	-15.28	22.6	9.7	65	21.59	29.8	15.1	14.7	24.9	20.3	24.9
Indri indri	49.59	-15.22	22.9	9.6	66	21.4	30	15.6	14.4	25.2	22	25.2
Indri indri	49.6	-15.77	21.9	9.5	65	21.67	29.1	14.6	14.5	24.3	19.7	24.3
Indri indri	49.6	-15.57	24.2	8.5	63	20.37	30.9	17.6	13.3	26.6	23.4	26.6
Indri indri	49.63	-15.37	23.9	8.5	63	20.63	30.7	17.3	13.4	25.9	23	26.3
Indri indri	49.73	-16.38	22.4	8.1	61	20.74	29	15.9	13.1	24.9	21.6	24.9
Indri indri	49.85	-16.44	24.3	7.3	59	19.26	30.4	18.1	12.3	26.6	23.5	26.6
Lemur catta	43.32	-22.32	24.5	12.4	63	25.69	33	13.4	19.6	27.4	21.4	27.4
Lemur catta	43.38	-21.88	24.4	12.3	63	25.51	32.7	13.2	19.5	27.2	20.9	27.3
Lemur catta	43.43	-22.8	24.2	12.6	63	26.08	33.1	13.2	19.9	27.3	20.7	27.3
Lemur catta	43.65	-23.66	24.4	12	62	25.39	33.4	14.1	19.3	27.4	21.2	27.4
Lemur catta	43.67	-23.33	24.2	12.4	63	25.74	33.3	13.7	19.6	27.3	21.1	27.3
Lemur catta	43.74	-24.03	24.6	12.1	61	26.52	33.6	13.9	19.7	27.7	21.3	27.7
Lemur catta	43.75	-24.09	24.6	12.1	61	26.7	33.5	13.9	19.6	27.7	21.3	27.7
Lemur catta	43.75	-24.05	24.6	12.1	61	26.61	33.6	13.9	19.7	27.7	21.3	27.7
Lemur catta	43.75	-24.03	24.5	12.2	61	26.59	33.5	13.8	19.7	27.6	21.2	27.6
Lemur catta	43.75	-23.53	24.4	12.4	63	25.26	33.5	13.9	19.6	27.4	21.2	27.4
Lemur catta	43.77	-24.19	24.5	12.1	62	26.73	33.3	13.8	19.5	27.6	21.2	27.6
Lemur catta	43.78	-24.13	24.5	12.2	61	26.58	33.4	13.7	19.7	27.5	21.2	27.5
Lemur catta	43.81	-21.28	25.1	12.6	63	25.55	33.1	13.4	19.7	27.9	21.5	27.9
Lemur catta	43.84	-24.39	24.5	11.9	61	27.11	33.3	13.8	19.5	27.7	21.2	27.7
Lemur catta	43.85	-24.4	24.2	12	61	27.02	32.9	13.5	19.4	27.3	20.8	27.3
Lemur catta	43.85	-24.38	24.2	12	61	26.91	32.9	13.5	19.4	27.3	20.9	27.3
Lemur catta	43.85	-23.33	24	13.3	64	26.44	33.3	12.8	20.5	27.1	20.4	27.1
Lemur catta	43.9	-24.01	24.5	12.8	63	27.18	33.6	13.3	20.3	27.6	21.1	27.6
Lemur catta	43.9	-23.43	24.2	13.3	64	26.52	33.5	12.9	20.6	27.2	20.9	27.2
Lemur catta	43.91	-22.06	24.8	14	66	26.03	33.6	12.5	21.1	27.6	21	27.6
Lemur catta	43.94	-23.19	23.6	14.1	65	27.18	33	11.5	21.5	26.6	19.7	26.6
Lemur catta	43.96	-24.65	24.1	11.7	60	27.43	32.6	13.4	19.2	27.3	20.7	27.3
Lemur catta	43.96	-24.61	24.1	11.8	60	27.52	32.8	13.4	19.4	27.3	20.7	27.3
Lemur catta	44.01	-23.13	24.2	14.5	66	27.76	33.7	11.8	21.9	27.3	20.9	27.3
Lemur catta	44.01	-20.75	25.4	12.1	62	24.96	33.1	13.8	19.3	28	21.8	28
Lemur catta	44.03	-24.74	24	11.7	60	27.59	32.5	13.3	19.2	27.1	20.6	27.1
Lemur catta	44.05	-24.72	24.1	11.8	60	27.72	32.7	13.3	19.4	27.2	20.7	27.2
Lemur catta	44.07	-23.48	24.6	14.2	65	28.09	34.1	12.3	21.8	27.6	21.1	27.6
Lemur catta	44.07	-20.88	25.2	12.5	63	25.04	33.2	13.4	19.8	27.8	21.6	27.8
Lemur catta	44.07	-20.81	25.3	12.4	63	24.97	33.2	13.6	19.6	27.9	21.7	27.9
Lemur catta	44.08	-24.76	23.9	11.8	61	27.72	32.5	13.2	19.3	27	20.5	27
Lemur catta	44.08	-23.35	23.8	14.5	66	27.97	33.2	11.3	21.9	26.8	19.7	26.8
Lemur catta	44.08	-20.89	25.2	12.5	63	25.04	33.2	13.4	19.8	27.8	21.6	27.8
Lemur catta	44.1	-21.7	25.2	13.9	65	25.87	34.1	12.8	21.3	27.9	21.4	27.9
Lemur catta	44.1	-20.85	25.2	12.5	63	24.99	33.2	13.4	19.8	27.8	21.6	27.8
Lemur catta	44.12	-21.68	25.2	13.9	64	25.99	34.2	12.7	21.5	27.9	21.4	27.9
Lemur catta	44.13	-23.51	24.2	14.5	65	28.61	33.8	11.7	22.1	27.2	20.7	27.2
Lemur catta	44.13	-21.83	25.4	14.1	64	26.21	34.5	12.8	21.7	28.1	21.6	28.1
Lemur catta	44.13	-21.73	25.3	14	65	26.1	34.3	12.8	21.5	28	21.5	28
Lemur catta	44.13	-21.71	25.3	14	65	25.95	34.2	12.8	21.4	28	21.5	28
Lemur catta	44.13	-21.7	25.3	13.9	64	26.24	34.2	12.7	21.5	28	21.4	28
Lemur catta	44.15	-20.79	25.3	12.5	62	24.98	33.3	13.4	19.9	27.9	21.7	27.9

Lemur catta	44.17	-22.64	19.1	13.9	68	23.44	28.3	7.9	20.4	21.6	15.7	21.6
Lemur catta	44.18	-22.1	24.6	14.8	66	25.82	34.2	11.9	22.3	27.2	20.8	27.2
Lemur catta	44.19	-22.68	20.7	14.6	67	24.44	30.3	8.8	21.5	23.2	17.1	23.2
Lemur catta	44.22	-21.08	25.1	13.2	64	25.38	33.5	12.9	20.6	27.7	21.4	27.7
Lemur catta	44.23	-24.22	24	13.9	64	28.59	33.5	12.1	21.4	27.1	20.6	27.1
Lemur catta	44.23	-20.79	25.2	12.7	62	24.8	33.3	13.1	20.2	27.7	21.6	27.7
Lemur catta	44.42	-23.67	24.7	15.5	64	30.4	35.2	11.2	24	27.7	21.1	27.7
Lemur catta	44.5	-24.33	24.5	14.9	65	29.93	34.4	11.8	22.6	27.7	21	27.7
Lemur catta	44.5	-22.28	24.7	15.7	66	25.66	35.2	11.5	23.7	27.2	21.8	27.3
Lemur catta	44.62	-25.11	24.3	13	61	30.83	33.7	12.7	21	27.8	20.9	27.8
Lemur catta	44.64	-23.63	25.3	16.5	66	30.32	35.9	11.1	24.8	28.2	21.6	28.2
Lemur catta	44.67	-22.76	23.8	16.8	66	26.74	35.3	10.1	25.2	26.3	20.9	26.5
Lemur catta	44.73	-22.82	21.9	15.5	66	25.28	32.3	9.1	23.2	24.3	18.1	24.4
Lemur catta	44.83	-22.68	22.8	15.8	66	25.06	33.5	9.9	23.6	25.2	19.9	25.4
Lemur catta	44.84	-22.46	22.4	15.2	67	23.98	32.5	9.9	22.6	24.7	18.7	24.8
Lemur catta	44.93	-24.87	24.7	15.1	65	31.12	34.9	12	22.9	28.1	21.2	28.1
Lemur catta	44.96	-21.98	24.8	15	64	26.66	35.2	11.8	23.4	27.3	20.8	27.5
Lemur catta	45.03	-21.88	25.3	15	63	27.74	35.8	12	23.8	27.8	21.1	28
Lemur catta	45.05	-21.72	25.7	14.8	61	28.68	36.3	12.3	24	28.3	21.5	28.6
Lemur catta	45.26	-22.44	22.9	14.4	66	23.51	32.4	10.8	21.6	25.2	19.4	25.2
Lemur catta	45.26	-22.37	23.1	14.4	66	23.94	32.8	11	21.8	25.4	19.5	25.5
Lemur catta	45.29	-22.32	23.5	14.5	65	24.42	33.3	11.2	22.1	25.8	19.8	25.9
Lemur catta	45.35	-22.28	21.9	13.7	65	23.01	31.2	10.3	20.9	24.2	18.5	24.2
Lemur catta	45.36	-25.46	23.4	10.8	60	26.69	31.4	13.6	17.8	26.4	20.1	26.4
Lemur catta	45.37	-22.63	21.9	13.7	67	22.25	30.9	10.5	20.4	24.1	18.6	24.1
Lemur catta	45.38	-22.48	21.8	13.6	67	22.18	30.8	10.6	20.2	24.1	18.6	24.1
Lemur catta	45.53	-21.28	25.2	14.3	62	26.59	35.3	12.5	22.8	27.6	21.3	27.8
Lemur catta	45.58	-21.42	24.9	14.2	62	26.81	35	12.3	22.7	27.3	20.9	27.5
Lemur catta	45.75	-22.48	21	13	65	23.48	30	10.2	19.8	23.3	17.5	23.3
Lemur catta	45.83	-24.83	23.9	14.4	65	29.46	33.7	11.8	21.9	27.1	20.7	27.1
Lemur catta	46.03	-24.78	24.2	14.9	66	29.95	34.2	11.8	22.4	27.5	20.9	27.5
Lemur catta	46.07	-24.71	23.8	15	67	29.53	33.8	11.5	22.3	27.1	20.6	27.1
Lemur catta	46.08	-25.17	23.2	12.8	63	28.96	32.2	12.1	20.1	26.5	21.3	26.5
Lemur catta	46.08	-24.81	24.1	14.9	66	29.84	34.1	11.8	22.3	27.5	20.9	27.5
Lemur catta	46.1	-24.55	24.2	15.2	66	29.84	34.3	11.5	22.8	27.5	21	27.5
Lemur catta	46.13	-24.68	23.4	14.8	66	28.81	33.3	11.2	22.1	26.6	20.2	26.6
Lemur catta	46.13	-24.68	23.4	14.8	66	28.81	33.3	11.2	22.1	26.6	20.2	26.6
Lemur catta	46.15	-24.73	23.5	14.8	67	29	33.3	11.3	22	26.7	20.3	26.7
Lemur catta	46.17	-24.76	23.6	14.8	66	29.2	33.5	11.4	22.1	26.8	20.4	26.8
Lemur catta	46.2	-24.87	24.2	14.9	67	29.66	34.1	11.9	22.2	27.5	20.9	27.5
Lemur catta	46.28	-24.98	24.3	14.8	66	29.59	34.1	12	22.1	27.6	20.9	27.6
Lemur catta	46.38	-25.03	24.5	14.7	65	29.65	34.3	12	22.3	27.8	22.5	27.8
Lemur catta	46.42	-24.85	24	14.8	67	28.63	33.8	12	21.8	27.3	20.7	27.3
Lemur catta	46.42	-24.47	22.7	14.1	65	28.15	32.4	10.9	21.5	25.9	19.7	25.9
Lemur catta	46.45	-24.69	23.2	14.2	66	28.22	32.8	11.5	21.3	26.4	20	26.4
Lemur catta	46.47	-24.85	23.8	14.4	67	28.21	33.3	11.9	21.4	26.9	20.5	26.9
Lemur catta	46.5	-24.7	23	13.9	66	27.72	32.4	11.5	20.9	26.1	19.8	26.1
Lemur catta	46.5	-22.6	22	12.1	62	26.17	30.8	11.5	19.3	24.7	18.2	24.7
Lemur catta	46.52	-25.05	21.9	12.3	65	26.24	30.5	11.7	18.8	24.9	20.1	24.9
Lemur catta	46.54	-24.83	23.8	13.8	66	27.75	33.2	12.3	20.9	27	20.6	27
Lemur catta	46.56	-24.97	23.9	13.4	66	27.55	32.9	12.6	20.3	27	21.9	27
Lemur catta	46.6	-24.91	21.3	12.1	65	25.58	29.7	11.3	18.4	24.2	18.2	24.2

Lemur catta	46.61	-24.82	23.5	13.1	65	27.03	32.5	12.4	20.1	26.5	21.7	26.6
Lemur catta	46.63	-24.97	22.8	12.5	65	26.35	31.5	12.3	19.2	25.8	20.9	25.8
Lemur catta	46.65	-25.17	22.5	11.4	64	25.6	30.5	12.8	17.7	25.4	20.4	25.4
Lemur catta	46.67	-25.18	23.7	11.2	63	25.9	31.7	14	17.7	26.7	21.5	26.7
Lemur catta	46.68	-24.53	20	12	65	24.94	28.3	10.1	18.2	22.8	17.1	22.8
Lemur catta	46.76	-22.44	20.9	11.3	62	25.68	29.2	11	18.2	23.6	17.8	23.6
Lemur catta	46.78	-25.08	22.5	10.7	62	24.72	30.3	13.3	17	25.4	20.6	25.4
Lemur catta	46.82	-25.03	23.3	10.1	61	23.92	30.8	14.4	16.4	26.1	21.4	26.1
Lemur catta	46.82	-22.17	17.7	10.9	61	24.87	26	8.4	17.6	20.2	14.8	20.2
Lemur catta	46.82	-22.17	17.7	10.9	61	24.87	26	8.4	17.6	20.2	14.8	20.2
Lemur catta	46.82	-22.16	17.6	10.9	62	24.82	25.9	8.4	17.5	20.1	14.7	20.1
Lemur catta	46.82	-22.16	17.6	10.9	62	24.82	25.9	8.4	17.5	20.1	14.7	20.1
Lemur catta	46.83	-22.16	15.7	10.8	62	24.03	24	6.8	17.2	18.2	13	18.2
Lemur catta	46.83	-22.16	15.7	10.8	62	24.03	24	6.8	17.2	18.2	13	18.2
Lemur catta	46.83	-22.15	16.4	10.8	62	24.43	24.7	7.4	17.3	18.9	13.6	18.9
Lemur catta	46.83	-22.15	16.4	10.8	62	24.43	24.7	7.4	17.3	18.9	13.6	18.9
Lemur catta	46.84	-22.16	14.4	10.7	62	23.55	22.6	5.6	17	16.9	11.8	16.9
Lemur catta	46.85	-22.21	17.1	10.8	62	24.51	25.3	8	17.3	19.6	14.3	19.6
Lemur catta	46.85	-22.16	13.8	10.6	63	23.15	21.8	5	16.8	16.2	11.2	16.2
Lemur catta	46.86	-22.16	13.9	10.6	63	23.51	21.9	5.1	16.8	16.3	11.2	16.3
Lemur catta	46.87	-22.18	12.4	10.5	63	22.7	20.3	3.7	16.6	14.7	9.9	14.7
Lemur catta	46.88	-25.07	23.1	9.3	61	23.01	30.2	15	15.2	25.8	21.2	25.8
Lemur catta	46.93	-21.85	19.7	10.9	60	25.49	28	10.1	17.9	22.3	15.9	22.3
Lemur catta	47.22	-21.2	16.9	10.7	60	25.52	25.3	7.7	17.6	19.5	15.3	19.5
Lepilemur dorsalis	47.97	-14.27	26.6	9.9	73	10.35	32.5	19	13.5	27.3	25	27.5
Lepilemur dorsalis	48.21	-14.30	26.1	10.6	73	12.18	32.4	17.9	14.5	26.9	24.2	27.1
Lepilemur dorsalis	48.33	-13.38	26.2	7.8	67	12.77	31.3	19.8	11.5	27.3	24.3	27.4
Lepilemur dorsalis	48.58	-13.47	25.6	9.5	68	13.07	31.6	17.8	13.8	26.6	23.6	26.7
Lepilemur dorsalis	48.78	-13.42	19.7	10.4	63	25.67	27.5	11.2	16.3	22.5	17.2	22.5
Lepilemur dorsalis	49.37	-12.78	25.2	9.5	73	12.47	31	18	13	26.3	24	26.3
Lepilemur edwardsi	44.58	-18.67	25.9	12	65	18.78	33.8	15.4	18.4	27.4	23.4	27.6
Lepilemur edwardsi	45.27	-16.32	26.5	10.9	69	14.54	32.8	17.1	15.7	27.4	24.2	27.7
Lepilemur edwardsi	46.48	-16.38	26.7	12.3	68	14.06	34.9	17	17.9	27.4	25	28.2
Lepilemur edwardsi	46.80	-16.32	26.3	12.8	69	13.65	34.9	16.5	18.4	26.9	24.7	27.7
Lepilemur edwardsi	46.82	-16.32	26.6	12.8	69	13.63	35.2	16.7	18.5	27.3	25	28
Lepilemur leucopus	43.80	-24.10	24.5	12.2	61	26.58	33.4	13.7	19.7	27.5	21.2	27.5
Lepilemur leucopus	46.05	-24.75	24.2	14.9	66	29.95	34.2	11.8	22.4	27.5	20.9	27.5
Lepilemur leucopus	46.33	-24.83	23.8	14.9	67	29.03	33.7	11.6	22.1	27.1	20.5	27.1
Lepilemur leucopus	46.40	-24.50	22.7	14.1	65	28.15	32.4	10.9	21.5	25.9	19.7	25.9
Lepilemur leucopus	46.55	-24.52	22	13.3	65	27.15	31.1	10.9	20.2	25	18.9	25
Lepilemur microdon	47.00	-22.00	17.4	10.5	61	24.79	25.4	8.3	17.1	19.9	14.5	19.9
Lepilemur microdon	47.4	-21.23	17.9	10.5	61	25.73	26	8.8	17.2	20.6	16.2	20.6
Lepilemur microdon	47.40	-21.20	17.9	10.5	61	25.73	26	8.8	17.2	20.6	16.2	20.6
Lepilemur microdon	47.42	-21.23	16.6	11.5	63	26.01	25	6.9	18.1	19.3	12.8	19.3
Lepilemur microdon	47.43	-20.68	17.2	11	63	25.73	25.3	7.9	17.4	19.9	13.8	19.9
Lepilemur microdon	47.47	-21.37	20.4	10.7	61	25.58	28.4	11.1	17.3	23	18.6	23.1
Lepilemur mustelinus	46.55	-24.52	22	13.3	65	27.15	31.1	10.9	20.2	25	18.9	25
Lepilemur mustelinus	46.88	-22.57	21.3	11.2	63	25.66	29.3	11.6	17.7	23.8	18.2	24
Lepilemur mustelinus	47.44	-20.69	17.2	11	63	25.79	25.4	8	17.4	19.9	13.9	19.9
Lepilemur mustelinus	47.44	-20.68	17.1	11	63	25.73	25.2	7.8	17.4	19.8	13.7	19.8
Lepilemur mustelinus	47.69	-20.08	15.6	11.1	63	25.92	23.9	6.3	17.6	18.3	12.3	18.3
Lepilemur mustelinus	47.85	-19.73	17.4	11.1	64	26.01	25.6	8.3	17.3	20.1	13.9	20.1

Lepilemur mustelinus	47.95	-18.49	18.3	11.7	66	24.42	26.5	8.9	17.6	20.8	14.9	20.8
Lepilemur mustelinus	47.95	-18.48	18.3	11.7	66	24.42	26.5	8.9	17.6	20.8	14.9	20.8
Lepilemur mustelinus	48.42	-18.78	19.5	11.1	64	26.34	27.7	10.4	17.3	22.3	17.2	22.3
Lepilemur mustelinus	48.47	-18.47	18.1	11.1	64	26.15	26.2	9.1	17.1	20.9	15.7	20.9
Lepilemur mustelinus	48.5	-18.85	20	10.8	63	25.9	28	11.1	16.9	22.8	17.7	22.8
Lepilemur mustelinus	48.97	-17.43	22.8	10	66	22.49	30	14.9	15.1	25.3	20.6	25.3
Lepilemur mustelinus	48.97	-17.43	22.8	10	66	22.49	30	14.9	15.1	25.3	20.6	25.3
Lepilemur mustelinus	49.43	-14.7	15.7	12.2	71	21.37	23.9	6.8	17.1	18	12.7	18
Lepilemur mustelinus	49.55	-14.31	21.4	11.4	70	20.57	29	12.8	16.2	23.6	19.3	23.6
Lepilemur mustelinus	49.57	-14.3	21.4	11.4	70	20.57	29	12.8	16.2	23.6	19.3	23.6
Lepilemur mustelinus	49.73	-16.38	22.4	8.1	61	20.74	29	15.9	13.1	24.9	21.6	24.9
Lepilemur mustelinus	50.13	-15.52	21.3	8.6	62	20.95	28.2	14.5	13.7	23.7	20.2	23.7
Lepilemur mustelinus	50.13	-15.52	21.3	8.6	62	20.95	28.2	14.5	13.7	23.7	20.2	23.7
Lepilemur ruficaudatus	44.05	-20.87	25.2	12.5	63	25.04	33.2	13.4	19.8	27.8	21.6	27.8
Lepilemur ruficaudatus	44.17	-21.70	25.3	13.9	64	26.24	34.2	12.7	21.5	28	21.4	28
Lepilemur ruficaudatus	44.63	-20.05	26	13.1	63	22.57	34.1	13.6	20.5	27.8	22.6	28.1
Lepilemur ruficaudatus	44.68	-22.78	23.4	16.6	66	26.58	34.7	9.8	24.9	25.9	20.4	26.1
Lepilemur ruficaudatus	44.77	-19.00	26	12.8	64	19.32	34.4	14.7	19.7	27.5	23	27.7
Lepilemur ruficaudatus	45.90	-16.05	26.9	10.4	70	12.7	33.1	18.4	14.7	27.7	24.9	28
Lepilemur ruficaudatus	43.43	-22.80	24.2	12.6	63	26.08	33.1	13.2	19.9	27.3	20.7	27.3
Microcebus griseorufus	43.24	-22.2	24.5	12.1	62	25.66	33	13.5	19.5	27.5	21	27.5
Microcebus griseorufus	43.38	-21.88	24.4	12.3	63	25.51	32.7	13.2	19.5	27.2	20.9	27.3
Microcebus griseorufus	43.41	-22.53	24.1	12.7	63	25.77	32.9	12.9	20	27.1	20.6	27.1
Microcebus griseorufus	43.43	-22.8	24.2	12.6	63	26.08	33.1	13.2	19.9	27.3	20.7	27.3
Microcebus griseorufus	43.52	-22.78	24.2	13	64	26.17	33.1	12.9	20.2	27.2	20.6	27.2
Microcebus griseorufus	43.67	-23.33	24.2	12.4	63	25.74	33.3	13.7	19.6	27.3	21.1	27.3
Microcebus griseorufus	43.75	-24.05	24.6	12.1	61	26.61	33.6	13.9	19.7	27.7	21.3	27.7
Microcebus griseorufus	43.77	-24.19	24.5	12.1	62	26.73	33.3	13.8	19.5	27.6	21.2	27.6
Microcebus griseorufus	43.85	-24.4	24.2	12	61	27.02	32.9	13.5	19.4	27.3	20.8	27.3
Microcebus griseorufus	43.9	-24.01	24.5	12.8	63	27.18	33.6	13.3	20.3	27.6	21.1	27.6
Microcebus griseorufus	44.03	-24.74	24	11.7	60	27.59	32.5	13.3	19.2	27.1	20.6	27.1
Microcebus griseorufus	44.57	-23.68	25	16.1	65	30.37	35.6	11.1	24.5	28	21.3	28
Microcebus griseorufus	44.63	-23.68	25.2	16.3	65	30.48	35.9	11.1	24.8	28.2	21.5	28.2
Microcebus griseorufus	44.64	-23.63	25.3	16.5	66	30.32	35.9	11.1	24.8	28.2	21.6	28.2
Microcebus griseorufus	44.65	-23.65	25.2	16.5	66	30.18	36	11.2	24.8	28.2	21.6	28.2
Microcebus griseorufus	45.23	-24.45	23.8	15.9	67	29.97	34.3	10.7	23.6	27	20.5	27
Microcebus griseorufus	46.33	-24.83	23.8	14.9	67	29.03	33.7	11.6	22.1	27.1	20.5	27.1
Microcebus griseorufus	46.38	-25.03	24.5	14.7	65	29.65	34.3	12	22.3	27.8	22.5	27.8
Microcebus griseorufus	46.4	-24.87	24	14.9	67	28.78	33.9	11.8	22.1	27.3	20.7	27.3
Microcebus griseorufus	46.82	-25.03	23.3	10.1	61	23.92	30.8	14.4	16.4	26.1	21.4	26.1
Microcebus myoxinus	44.45	-19.25	26.2	11.9	63	20.31	33.8	15.2	18.6	28	23.1	28
Microcebus myoxinus	44.75	-18.74	25.4	12.6	65	18.66	33.8	14.5	19.3	26.8	22.9	27.1
Microcebus myoxinus	44.81	-19.13	26.2	13	64	19.78	34.8	14.6	20.2	27.7	23.1	28
Microcebus myoxinus	44.82	-19.1	25.9	13	64	19.57	34.5	14.3	20.2	27.4	22.9	27.7
Microcebus myoxinus	45.36	-16.43	26.1	11.2	69	14.51	32.7	16.5	16.2	27	23.8	27.3
Microcebus ravelobensis	46.79	-16.34	26.3	12.8	69	13.65	34.9	16.5	18.4	26.9	24.7	27.7
Microcebus ravelobensis	46.83	-16.3	26.3	12.8	69	13.57	34.9	16.5	18.4	26.9	24.7	27.7
Microcebus ravelobensis	46.95	-16.12	26.4	12.9	68	14.45	35	16.1	18.9	27.3	24.2	27.8
Microcebus ravelobensis	47.15	-16.23	26.5	13	69	13.61	35.1	16.4	18.7	27.3	24.4	27.8
Microcebus ravelobensis	48.2	-14.87	26.3	11.8	70	15.03	34	17.2	16.8	27.3	23.9	27.5
Microcebus rufus	47.44	-20.7	18.1	11	63	25.82	26.2	8.9	17.3	20.8	16.3	20.8
Microcebus rufus	47.44	-20.69	17.2	11	63	25.79	25.4	8	17.4	19.9	13.9	19.9

Microcebus rufus	47.44	-20.68	17.1	11	63	25.73	25.2	7.8	17.4	19.8	13.7	19.8
Microcebus rufus	47.64	-20.39	17	10.9	62	26.05	25.2	7.8	17.4	19.7	13.6	19.7
Microcebus rufus	47.67	-20.06	15.9	11.2	63	25.99	24.2	6.5	17.7	18.6	12.5	18.6
Microcebus rufus	47.69	-20.08	15.6	11.1	63	25.92	23.9	6.3	17.6	18.3	12.3	18.3
Microcebus rufus	47.69	-20.08	15.6	11.1	63	25.92	23.9	6.3	17.6	18.3	12.3	18.3
Microcebus rufus	47.76	-19.92	17.8	11.2	64	26.07	26	8.6	17.4	20.6	14.4	20.6
Microcebus sambiranensls	48.3	-14.03	20.1	12.1	73	15.44	27.8	11.3	16.5	21.5	17.9	21.5
Microcebus sambiranensls	48.42	-14.02	17.5	12.5	73	16.85	25.7	8.6	17.1	19.1	15.1	19.1
Microcebus sambiranensls	48.42	-13.98	22	11.7	71	15.02	29.5	13.2	16.3	23.3	19.9	23.4
Microcebus sambiranensls	48.43	-14	19.2	12.4	72	16.65	27.3	10.1	17.2	20.8	16.8	20.8
Microcebus sambiranensls	48.57	-13.63	25.8	10	68	13.28	32	17.5	14.5	26.9	23.9	27
Microcebus tavaratra	49.05	-12.93	26.6	9.6	70	11.67	32.8	19.1	13.7	27.4	25	27.6
Microcebus tavaratra	49.06	-12.93	26	9.8	70	11.92	32.2	18.3	13.9	26.8	24.3	27
Microcebus tavaratra	49.07	-12.48	25	8.8	71	12.33	30.7	18.4	12.3	26.1	23.8	26.1
Microcebus tavaratra	49.09	-12.93	26	9.9	70	11.92	32.3	18.3	14	26.9	24.3	27
Microcebus tavaratra	49.1	-12.95	26.1	9.9	69	11.96	32.5	18.3	14.2	26.9	24.4	27.1
Microcebus tavaratra	49.11	-12.91	25.9	9.8	70	12.05	32.2	18.2	14	26.8	24.2	26.9
Microcebus tavaratra	49.23	-12.87	24.6	9.9	71	12.82	30.8	17	13.8	25.7	22.7	25.7
Mirza coquereli	44.17	-22.17	24.3	14.9	66	25.53	34	11.6	22.4	26.8	20.5	26.8
Mirza coquereli	44.58	-18.78	26.1	12	65	19.09	33.9	15.5	18.4	27.6	23.5	27.8
Mirza coquereli	44.63	-20.04	26	13.1	63	22.57	34.1	13.6	20.5	27.8	22.6	28.1
Mirza coquereli	44.69	-22.78	23.4	16.6	66	26.58	34.7	9.8	24.9	25.9	20.4	26.1
Mirza coquereli	45.27	-16.33	26.5	10.9	69	14.54	32.8	17.1	15.7	27.4	24.2	27.7
Phaner pallescens	43.43	-22.8	24.2	12.6	63	26.08	33.1	13.2	19.9	27.3	20.7	27.3
Phaner pallescens	44.17	-22.17	24.3	14.9	66	25.53	34	11.6	22.4	26.8	20.5	26.8
Phaner pallescens	44.58	-18.78	26.1	12	65	19.09	33.9	15.5	18.4	27.6	23.5	27.8
Phaner pallescens	44.63	-20.04	26	13.1	63	22.57	34.1	13.6	20.5	27.8	22.6	28.1
Phaner pallescens	44.69	-22.78	23.4	16.6	66	26.58	34.7	9.8	24.9	25.9	20.4	26.1
Phaner pallescens	45.27	-16.33	26.5	10.9	69	14.54	32.8	17.1	15.7	27.4	24.2	27.7
Phaner pallescens	46.55	-24.52	22	13.3	65	27.15	31.1	10.9	20.2	25	18.9	25
Propithecus candidus	49.42	-14.68	16.3	12.3	71	21.51	24.5	7.4	17.1	18.6	13.3	18.6
Propithecus candidus	49.43	-14.54	19.8	12.2	70	21.61	28	10.8	17.2	22.1	16.7	22.1
Propithecus candidus	49.44	-14.78	18.7	12.1	70	22.15	26.9	9.8	17.1	21.1	15.5	21.1
Propithecus candidus	49.44	-14.74	16.8	12.2	71	21.69	25.1	8	17.1	19.1	13.7	19.1
Propithecus candidus	49.61	-14.43	19.9	11.7	70	21.46	27.9	11.2	16.7	22.2	17.8	22.2
Propithecus candidus	49.62	-14.44	19	11.8	70	21.36	27.1	10.3	16.8	21.3	16.9	21.3
Propithecus candidus	49.74	-14.45	15.4	11.7	70	20.93	23.4	6.9	16.5	17.7	13.3	17.7
Propithecus candidus	49.74	-14.44	17.8	11.7	70	21.34	25.8	9.2	16.6	20.1	15.7	20.1
Propithecus candidus	49.74	-14.44	17.8	11.7	70	21.34	25.8	9.2	16.6	20.1	15.7	20.1
Propithecus candidus	49.76	-14.44	20.7	10.9	68	21.1	28.4	12.5	15.9	23	18.5	23
Propithecus candidus	49.76	-14.43	20.4	11	68	21.19	28.1	12.1	16	22.7	18.2	22.7
Propithecus candidus	50.27	-14.89	24.8	7.9	63	17.15	30.8	18.3	12.5	26.4	23.9	26.8
Propithecus coquereli	46.52	-15.62	27	10.3	70	12.09	33.2	18.5	14.7	27.7	25.1	28
Propithecus coquereli	46.67	-15.47	26.9	10.8	68	13.53	33.5	17.7	15.8	27.7	24.8	28
Propithecus coquereli	46.69	-15.48	26.9	10.8	67	13.63	33.7	17.6	16.1	27.7	24.8	28.1
Propithecus coquereli	46.79	-16.34	26.3	12.8	69	13.65	34.9	16.5	18.4	26.9	24.7	27.7
Propithecus coquereli	46.82	-16.3	26.6	12.8	69	13.63	35.2	16.7	18.5	27.3	25	28
Propithecus coquereli	46.83	-15.5	26.8	11.5	65	15.02	34.1	16.6	17.5	27.6	24.5	28.1
Propithecus coquereli	46.95	-16.15	26.6	12.9	68	14.29	35.2	16.3	18.9	27.5	25	28
Propithecus coquereli	46.95	-16.12	26.4	12.9	68	14.45	35	16.1	18.9	27.3	24.2	27.8
Propithecus coquereli	46.96	-16.23	26.2	12.9	69	13.5	34.7	16.2	18.5	26.9	24.1	27.5
Propithecus coquereli	47.08	-15.81	26.9	13.1	64	17.69	35.6	15.2	20.4	28	24.2	28.6

Propithecus coquereli	47.12	-15.8	26.8	13.2	63	17.91	35.7	15	20.7	27.9	24.1	28.5
Propithecus coquereli	47.12	-15.66	26.8	13.1	62	18.48	35.6	14.8	20.8	27.9	24	28.5
Propithecus coquereli	47.14	-16.23	26.3	13	69	13.61	34.9	16.3	18.6	27.1	24.2	27.6
Propithecus coquereli	47.15	-16.23	26.5	13	69	13.61	35.1	16.4	18.7	27.3	24.4	27.8
Propithecus coquereli	47.23	-15.01	26.9	11.2	67	13.25	34	17.5	16.5	27.7	24.8	28
Propithecus coquereli	47.23	-15	26.9	11.2	68	13.15	33.9	17.5	16.4	27.6	24.8	27.9
Propithecus coquereli	47.24	-15.05	26.7	11.4	67	13.67	33.9	17.1	16.8	27.5	24.6	27.8
Propithecus coquereli	47.48	-15.5	26.6	13.1	63	18.37	35.7	15.2	20.5	27.8	23.8	28.3
Propithecus coquereli	47.68	-15.33	25.8	12.9	66	17.27	34.6	15.2	19.4	26.9	23.1	27.3
Propithecus coquereli	47.71	-14.94	26.3	11.7	70	12.77	33.9	17.2	16.7	27	24.3	27.4
Propithecus coquereli	47.74	-15.16	26.1	12.5	68	15.73	34.3	16.1	18.2	27.2	23.7	27.5
Propithecus coquereli	47.78	-14.79	25.9	11.3	71	11.25	33.2	17.3	15.9	26.5	24.1	26.8
Propithecus coquereli	48.2	-14.87	26.3	11.8	70	15.03	34	17.2	16.8	27.3	23.9	27.5
Propithecus coquereli	48.21	-14.86	25.7	11.9	71	15.01	33.4	16.7	16.7	26.8	23.4	27
Propithecus coquereli	48.3	-15.26	25.2	12.3	69	17.9	33.7	15.9	17.8	26.8	22.9	26.9
Propithecus coronatus	45.39	-18.28	21.7	13.1	66	17.85	30.4	10.8	19.6	23	18.9	23.3
Propithecus coronatus	45.43	-18.53	22.2	13.4	67	18.17	31.1	11.1	20	23.5	19.4	23.8
Propithecus coronatus	45.92	-16.05	26.9	10.4	71	12.69	33	18.4	14.6	27.6	24.9	27.9
Propithecus coronatus	45.93	-17.08	26.2	12.8	69	14.67	34.4	16	18.4	27	24.3	27.6
Propithecus coronatus	45.93	-16.1	26.9	10.5	70	12.74	33.2	18.3	14.9	27.7	24.9	28
Propithecus coronatus	45.93	-16.08	26.9	10.5	70	12.77	33.2	18.4	14.8	27.6	24.9	28
Propithecus coronatus	45.95	-16.12	26.9	10.6	70	12.7	33.2	18.2	15	27.7	24.9	28
Propithecus coronatus	45.97	-16.18	27	10.8	70	12.93	33.5	18.1	15.4	27.7	24.9	28.1
Propithecus coronatus	45.98	-17.08	26.6	12.8	69	14.57	34.7	16.4	18.3	27.3	24.6	28
Propithecus coronatus	46	-16.15	26.9	10.8	70	12.77	33.5	18.2	15.3	27.7	24.9	28.1
Propithecus coronatus	46.02	-16.15	26.9	10.8	70	12.87	33.5	18.1	15.4	27.6	24.9	28.1
Propithecus coronatus	46.04	-18.78	22.9	13.6	67	18.47	31.9	11.7	20.2	24.4	20	24.5
Propithecus coronatus	46.05	-18.78	22.8	13.5	67	18.4	31.7	11.6	20.1	24.3	19.9	24.4
Propithecus coronatus	46.22	-15.72	26.7	9.5	73	10.74	32.3	19.3	13	27.3	25	27.6
Propithecus coronatus	46.24	-15.75	26.9	9.6	73	10.8	32.5	19.4	13.1	27.5	25.2	27.8
Propithecus coronatus	46.4	-16.55	26.9	12.3	69	13.88	35	17.3	17.7	27.5	25.2	28.4
Propithecus coronatus	46.48	-16.38	26.7	12.3	68	14.06	34.9	17	17.9	27.4	25	28.2
Propithecus coronatus	47.28	-18.17	17.4	12.3	67	21.37	25.8	7.5	18.3	19.4	14.2	19.4
Propithecus deckeni	44.07	-17.79	25.8	9.7	64	17.97	32.3	17.2	15.1	27.5	23.1	27.5
Propithecus deckeni	44.21	-17.81	25.8	10.1	65	17.65	32.4	17	15.4	27.4	23.2	27.4
Propithecus deckeni	44.33	-16.93	26.6	11.2	67	15.66	33.5	16.9	16.6	27.9	24.2	28
Propithecus deckeni	44.68	-16.87	26.4	11.7	67	15.35	33.7	16.3	17.4	27.4	24	27.8
Propithecus deckeni	44.72	-18.98	26.2	12.6	64	19.3	34.5	15	19.5	27.8	23.2	28
Propithecus deckeni	44.72	-18.7	25.8	12.4	64	18.71	34	14.9	19.1	27.2	23.3	27.4
Propithecus deckeni	44.72	-16.88	25.7	11.9	68	15.07	33	15.7	17.3	26.7	23.4	27
Propithecus deckeni	44.73	-16.94	26.1	11.9	68	15.35	33.4	16	17.4	27.1	23.7	27.4
Propithecus deckeni	44.75	-18.74	25.4	12.6	65	18.66	33.8	14.5	19.3	26.8	22.9	27.1
Propithecus deckeni	44.76	-18.99	26	12.8	64	19.32	34.4	14.7	19.7	27.5	23	27.7
Propithecus deckeni	44.81	-19.13	26.2	13	64	19.78	34.8	14.6	20.2	27.7	23.1	28
Propithecus deckeni	44.83	-19.13	25.9	13.1	64	19.46	34.5	14.2	20.3	27.4	22.8	27.6
Propithecus deckeni	44.83	-19	25	13	65	19.12	33.7	13.7	20	26.5	22.1	26.8
Propithecus deckeni	44.92	-18.72	23.6	13	65	18.34	32.3	12.5	19.8	24.9	20.7	25.3
Propithecus deckeni	45.07	-16.09	26.6	10.3	68	14.37	32.6	17.6	15	27.5	24.4	27.8
Propithecus deckeni	45.09	-16.09	26.5	10.3	68	14.4	32.6	17.6	15	27.5	24.3	27.7
Propithecus deckeni	45.1	-16.18	26.4	10.5	68	14.54	32.6	17.3	15.3	27.4	24.2	27.7
Propithecus deckeni	45.13	-16.08	26.6	10.2	68	14.4	32.7	17.7	15	27.6	24.4	27.8
Propithecus deckeni	45.15	-16.13	26.5	10.3	68	14.44	32.5	17.5	15	27.5	24.3	27.7

Propithecus deckeni	45.2	-16.28	26.5	10.7	69	14.35	32.7	17.3	15.4	27.5	24.3	27.7
Propithecus deckeni	45.2	-16.27	26.5	10.7	69	14.39	32.7	17.3	15.4	27.5	24.3	27.7
Propithecus deckeni	45.21	-16.28	26.5	10.7	69	14.35	32.7	17.3	15.4	27.5	24.3	27.7
Propithecus deckeni	45.21	-16.15	26.5	10.4	68	14.48	32.6	17.4	15.2	27.5	24.3	27.7
Propithecus deckeni	45.25	-16	26.5	10	67	14.33	32.5	17.7	14.8	27.5	24.3	27.7
Propithecus deckeni	45.34	-16.1	26.7	10.1	67	14.47	32.7	17.7	15	27.7	24.5	27.9
Propithecus deckeni	45.35	-16.47	26	11.3	69	14.44	32.7	16.4	16.3	26.9	23.7	27.2
Propithecus deckeni	45.36	-16.43	26.1	11.2	69	14.51	32.7	16.5	16.2	27	23.8	27.3
Propithecus deckeni	45.36	-16.01	26.7	10	67	14.08	32.7	17.9	14.8	27.7	24.6	27.9
Propithecus deckeni	45.39	-18.28	21.7	13.1	66	17.85	30.4	10.8	19.6	23	18.9	23.3
Propithecus deckeni	45.4	-18.3	21.7	13.1	66	17.75	30.4	10.8	19.6	23	18.9	23.3
Propithecus deckeni	45.4	-18.27	21.6	13.1	67	17.66	30.2	10.7	19.5	22.9	18.8	23.2
Propithecus deckeni	45.42	-18.26	21.3	13.1	67	17.59	30	10.5	19.5	22.6	18.6	22.9
Propithecus deckeni	45.43	-18.53	22.2	13.4	67	18.17	31.1	11.1	20	23.5	19.4	23.8
Propithecus deckeni	45.48	-15.97	26.8	9.9	68	13.67	32.6	18.1	14.5	27.7	24.7	27.9
Propithecus deckeni	45.78	-16.98	25.4	12.6	69	14.57	33.4	15.2	18.2	26.2	23.5	26.7
Propithecus deckeni	45.83	-16	26.7	10.2	70	12.69	32.7	18.3	14.4	27.4	24.7	27.7
Propithecus deckeni	45.87	-16	26.8	10.2	70	12.65	32.8	18.4	14.4	27.5	24.8	27.8
Propithecus deckeni	45.88	-16.22	26.8	10.8	70	13.24	33.3	17.9	15.4	27.6	24.7	27.9
Propithecus deckeni	45.9	-16.08	26.9	10.5	71	12.76	33	18.3	14.7	27.6	24.9	27.9
Propithecus deckeni	45.9	-16.05	26.9	10.4	70	12.7	33.1	18.4	14.7	27.7	24.9	28
Propithecus deckeni	45.9	-16.03	26.9	10.3	71	12.61	32.9	18.5	14.4	27.7	24.9	27.9
Propithecus deckeni	45.93	-17.08	26.2	12.8	69	14.67	34.4	16	18.4	27	24.3	27.6
Propithecus deckeni	45.93	-16.1	26.9	10.5	70	12.74	33.2	18.3	14.9	27.7	24.9	28
Propithecus deckeni	45.95	-16.12	26.9	10.6	70	12.7	33.2	18.2	15	27.7	24.9	28
Propithecus deckeni	45.97	-17.06	26.4	12.8	69	14.65	34.6	16.2	18.4	27.2	24.5	27.8
Propithecus deckeni	45.97	-16.25	26.9	11	70	13.18	33.6	17.9	15.7	27.7	24.8	28.1
Propithecus deckeni	45.97	-16.18	27	10.8	70	12.93	33.5	18.1	15.4	27.7	24.9	28.1
Propithecus deckeni	45.98	-17.07	26.6	12.8	69	14.57	34.8	16.5	18.3	27.3	24.7	28
Propithecus deckeni	45.98	-16.25	26.9	11	70	13.18	33.6	17.9	15.7	27.7	24.8	28.1
Propithecus deckeni	45.99	-17.07	26.8	12.7	69	14.6	35	16.7	18.3	27.6	24.9	28.2
Propithecus deckeni	46	-16.15	26.9	10.8	70	12.77	33.5	18.2	15.3	27.7	24.9	28.1
Propithecus deckeni	46.02	-16.15	26.9	10.8	70	12.87	33.5	18.1	15.4	27.6	24.9	28.1
Propithecus deckeni	46.03	-17.12	26.9	12.8	69	14.42	35	16.7	18.3	27.6	24.9	28.3
Propithecus deckeni	46.22	-15.72	26.7	9.5	73	10.74	32.3	19.3	13	27.3	25	27.6
Propithecus diadema	47.77	-19.68	16.4	11.1	63	25.74	24.7	7.2	17.5	19	13	19
Propithecus diadema	47.84	-19.71	17.4	11.1	64	26.01	25.6	8.3	17.3	20.1	13.9	20.1
Propithecus diadema	47.95	-18.47	18.3	11.7	66	24.35	26.5	8.8	17.7	20.7	14.8	20.7
Propithecus diadema	47.95	-18.41	17.9	11.7	66	24.29	26.1	8.5	17.6	20.4	14.5	20.4
Propithecus diadema	48.05	-18.92	19.4	11.3	65	25.74	27.5	10.2	17.3	22.1	15.8	22.1
Propithecus diadema	48.23	-19.67	21.2	10.6	65	24.45	29.1	12.8	16.3	24	19.1	24
Propithecus diadema	48.33	-19.5	21	10.5	64	24.54	28.7	12.5	16.2	23.7	18.7	23.7
Propithecus diadema	48.42	-18.5	19.8	11.4	65	26.26	28	10.5	17.5	22.6	17.4	22.6
Propithecus diadema	48.43	-18.93	19	10.8	64	25.77	27.1	10.3	16.8	21.8	16.7	21.8
Propithecus diadema	48.43	-18.79	19.5	11.1	64	26.34	27.7	10.4	17.3	22.3	17.2	22.3
Propithecus diadema	48.45	-18.98	19.1	10.7	64	25.65	27.1	10.5	16.6	21.9	16.8	21.9
Propithecus diadema	48.46	-18.97	18.7	10.6	63	25.94	26.7	10	16.7	21.5	16.4	21.5
Propithecus diadema	48.47	-18.97	18.2	10.6	63	26.15	26.3	9.7	16.6	21.1	15.9	21.1
Propithecus diadema	48.5	-18.89	19.6	10.8	64	25.81	27.6	10.8	16.8	22.4	17.3	22.4
Propithecus diadema	48.76	-17.68	19.4	11	66	24.19	27.4	10.8	16.6	21.9	16	21.9
Propithecus diadema	48.77	-17.52	19.4	11.2	66	23.97	27.6	10.8	16.8	22	16.1	22
Propithecus diadema	48.77	-16.94	19.3	11.1	66	23.89	27.5	10.7	16.8	21.8	17.2	22

Propithecus diadema	48.78	-18.41	19.7	10.4	63	25.67	27.5	11.2	16.3	22.5	17.2	22.5
Propithecus diadema	48.8	-16.28	18.8	11.4	66	23.55	27.2	10.1	17.1	21.2	16.5	21.3
Propithecus diadema	48.81	-18.7	22.7	9.4	62	22.94	29.9	14.9	15	25.3	20.5	25.3
Propithecus diadema	48.98	-17.73	22	9.9	63	23.91	29.7	14	15.7	24.8	19.7	24.8
Propithecus diadema	49.09	-18.05	22.5	9.1	60	23.97	30	14.9	15.1	25.3	20	25.3
Propithecus diadema	49.13	-16.85	20.2	9.9	64	23.63	27.8	12.5	15.3	22.9	17.9	22.9
Propithecus diadema	49.42	-16.85	22.7	8.2	63	20.84	29.2	16.2	13	25.1	21.9	25.1
Propithecus diadema	49.73	-16.38	22.4	8.1	61	20.74	29	15.9	13.1	24.9	21.6	24.9
Propithecus edwardsi	47	-22.23	19.7	10.7	62	25.35	27.6	10.4	17.2	22.4	16.6	22.4
Propithecus edwardsi	47.02	-22.22	20.2	10.7	61	25.34	28.1	10.8	17.3	22.8	17	22.8
Propithecus edwardsi	47.02	-22.15	17.4	10.5	61	24.79	25.4	8.3	17.1	19.9	14.5	19.9
Propithecus edwardsi	47.04	-22.16	16.3	10.4	61	24.38	24.3	7.5	16.8	18.8	13.5	18.8
Propithecus edwardsi	47.27	-21.72	18.5	10.2	60	25.37	26.4	9.5	16.9	21.1	15.3	21.1
Propithecus edwardsi	47.34	-21.83	19	10.1	61	25.16	26.5	10.1	16.4	21.6	15.8	21.6
Propithecus edwardsi	47.35	-21.78	18.7	10.1	60	25.16	26.4	9.8	16.6	21.3	15.5	21.3
Propithecus edwardsi	47.36	-21.82	19	10.1	61	25.12	26.6	10.1	16.5	21.6	17.3	21.6
Propithecus edwardsi	47.38	-21.23	17.9	10.5	61	25.73	26	8.8	17.2	20.6	16.2	20.6
Propithecus edwardsi	47.4	-22.95	22.8	9.8	60	24.59	30.2	13.9	16.3	25.6	21.1	25.6
Propithecus edwardsi	47.41	-21.74	19	10	60	25.26	26.6	10.2	16.4	21.7	17.3	21.7
Propithecus edwardsi	47.41	-21.51	18.4	10.2	61	25.37	26.2	9.6	16.6	21	16.6	21
Propithecus edwardsi	47.43	-21.29	18.5	10.4	61	25.57	26.5	9.5	17	21.2	16.8	21.2
Propithecus edwardsi	47.43	-20.71	17.7	11	63	25.61	25.8	8.5	17.3	20.4	14.4	20.4
Propithecus edwardsi	47.43	-20.69	17.2	11	63	25.73	25.3	7.9	17.4	19.9	13.8	19.9
Propithecus edwardsi	47.44	-20.7	18.1	11	63	25.82	26.2	8.9	17.3	20.8	16.3	20.8
Propithecus edwardsi	47.44	-20.69	17.2	11	63	25.79	25.4	8	17.4	19.9	13.9	19.9
Propithecus edwardsi	47.45	-20.71	17.8	11	63	25.56	25.8	8.6	17.2	20.5	14.5	20.5
Propithecus edwardsi	47.46	-21.3	19.7	10.6	61	25.45	27.7	10.5	17.2	22.3	18	22.4
Propithecus edwardsi	47.46	-21.2	18	10.4	61	25.77	26	9	17	20.7	16.2	20.7
Propithecus edwardsi	47.47	-21	18	10.7	62	25.72	26.1	9	17.1	20.6	16.3	20.7
Propithecus edwardsi	47.49	-21.59	18.9	10.1	61	25.35	26.6	10.1	16.5	21.6	17.2	21.6
Propithecus edwardsi	47.49	-21.54	20.2	10.3	60	25.35	28	11.1	16.9	22.8	18.5	23
Propithecus edwardsi	47.49	-21.39	20.3	10.6	61	25.5	28.3	11.1	17.2	22.9	18.5	23.1
Propithecus edwardsi	47.53	-21.25	20.5	10.7	61	25.61	28.6	11.2	17.4	23.1	18.7	23.2
Propithecus edwardsi	47.54	-21.12	18	10.4	61	25.52	25.9	9.1	16.8	20.5	16.1	20.7
Propithecus edwardsi	47.58	-20.7	20.6	11.1	63	25.37	28.8	11.3	17.5	23.2	18.8	23.4
Propithecus edwardsi	47.62	-20.33	16.6	11	62	25.95	24.8	7.3	17.5	19.3	13.2	19.3
Propithecus edwardsi	47.7	-21.37	20.6	10.3	61	25.06	28.3	11.6	16.7	23.2	18.7	23.3
Propithecus edwardsi	47.78	-19.93	19.9	11.4	65	25.47	28.1	10.7	17.4	22.6	17.9	22.7
Propithecus edwardsi	47.8	-20.4	19.8	10.8	63	25.4	27.8	10.8	17	22.4	17.8	22.5
Propithecus edwardsi	47.83	-22.35	23.3	7.9	56	22.87	29.8	15.8	14	26.1	21.4	26.1
Propithecus edwardsi	48.27	-20.19	21.9	9.7	62	23.71	29.3	13.8	15.5	24.5	19.9	24.6
Propithecus edwardsi	48.68	-20.05	23.5	7.8	59	20.79	29.6	16.5	13.1	25.9	23	25.9
Propithecus perrieri	49.07	-12.93	25.8	9.9	70	12.06	32	18	14	26.6	24	26.8
Propithecus perrieri	49.34	-12.92	23.3	10.1	72	13.55	29.6	15.7	13.9	24.5	21.4	24.5
Propithecus perrieri	49.37	-12.78	25.2	9.5	73	12.47	31	18	13	26.3	24	26.3
Propithecus perrieri	49.49	-12.75	25.9	9	70	12.38	31.6	18.9	12.7	27	24.7	27
Propithecus perrieri	49.5	-12.77	25.7	9.1	72	12.3	31.4	18.8	12.6	26.8	24.5	26.8
Propithecus perrieri	49.53	-12.81	25.3	9.2	71	12.72	31	18.2	12.8	26.5	24.1	26.5
Propithecus perrieri	49.53	-12.81	25.3	9.2	71	12.72	31	18.2	12.8	26.5	24.1	26.5
Propithecus perrieri	49.57	-12.73	25	9.1	72	12.76	30.7	18.1	12.6	26.2	23.7	26.2
Propithecus tattersalli	49.45	-13.15	25.3	10.1	71	13.06	31.6	17.5	14.1	26.4	23.4	26.4
Propithecus tattersalli	49.45	-13.02	26.2	9.6	71	12.28	32.2	18.7	13.5	27.2	24.4	27.3

Propithecus tattersalli	49.62	-13.26	23.9	9.9	72	14.31	30	16.3	13.7	25.2	21.8	25.2
Propithecus tattersalli	49.68	-13.15	25.6	9.1	71	13.33	31.3	18.6	12.7	26.9	24.4	26.9
Propithecus tattersalli	49.68	-13.12	25.5	9.2	71	13.44	31.2	18.4	12.8	26.8	24.2	26.8
Propithecus tattersalli	49.7	-13.11	24.5	9.4	72	13.9	30.3	17.3	13	25.8	23.1	25.8
Propithecus tattersalli	49.76	-13.22	23.1	9.7	72	14.93	29.2	15.9	13.3	24.6	21.6	24.6
Propithecus tattersalli	49.82	-13.07	25.3	8.8	72	13.73	30.9	18.7	12.2	26.7	23.9	26.7
Propithecus verreauxi	43.27	-22.28	24.5	12.3	63	25.57	33	13.5	19.5	27.4	21	27.4
Propithecus verreauxi	43.43	-22.8	24.2	12.6	63	26.08	33.1	13.2	19.9	27.3	20.7	27.3
Propithecus verreauxi	43.62	-21.92	24.6	13	64	25.8	33.1	12.9	20.2	27.5	21	27.5
Propithecus verreauxi	43.75	-24.05	24.6	12.1	61	26.61	33.6	13.9	19.7	27.7	21.3	27.7
Propithecus verreauxi	43.77	-24.19	24.5	12.1	62	26.73	33.3	13.8	19.5	27.6	21.2	27.6
Propithecus verreauxi	43.78	-24.13	24.5	12.2	61	26.58	33.4	13.7	19.7	27.5	21.2	27.5
Propithecus verreauxi	43.81	-21.28	25.1	12.6	63	25.55	33.1	13.4	19.7	27.9	21.5	27.9
Propithecus verreauxi	43.85	-23.33	24	13.3	64	26.44	33.3	12.8	20.5	27.1	20.4	27.1
Propithecus verreauxi	43.9	-23.43	24.2	13.3	64	26.52	33.5	12.9	20.6	27.2	20.9	27.2
Propithecus verreauxi	43.9	-21.2	25.2	12.7	63	25.47	33.2	13.3	19.9	27.9	21.5	27.9
Propithecus verreauxi	43.91	-22.06	24.8	14	66	26.03	33.6	12.5	21.1	27.6	21	27.6
Propithecus verreauxi	43.93	-22.05	24.8	14	65	25.98	33.8	12.5	21.3	27.6	21.1	27.6
Propithecus verreauxi	43.96	-24.61	24.1	11.8	60	27.52	32.8	13.4	19.4	27.3	20.7	27.3
Propithecus verreauxi	44.01	-20.75	25.4	12.1	62	24.96	33.1	13.8	19.3	28	21.8	28
Propithecus verreauxi	44.02	-20.78	25.4	12.2	62	25.15	33.2	13.8	19.4	28	21.8	28
Propithecus verreauxi	44.03	-24.74	24	11.7	60	27.59	32.5	13.3	19.2	27.1	20.6	27.1
Propithecus verreauxi	44.06	-20.8	25.3	12.3	62	25.01	33.2	13.6	19.6	27.9	21.7	27.9
Propithecus verreauxi	44.07	-20.88	25.2	12.5	63	25.04	33.2	13.4	19.8	27.8	21.6	27.8
Propithecus verreauxi	44.07	-20.81	25.3	12.4	63	24.97	33.2	13.6	19.6	27.9	21.7	27.9
Propithecus verreauxi	44.08	-23.35	23.8	14.5	66	27.97	33.2	11.3	21.9	26.8	19.7	26.8
Propithecus verreauxi	44.09	-23.52	24.8	14.2	65	28.19	34.3	12.5	21.8	27.8	21.3	27.8
Propithecus verreauxi	44.09	-21.5	24.7	13.6	65	25.7	33.3	12.4	20.9	27.3	20.9	27.3
Propithecus verreauxi	44.12	-21.72	25.3	14	65	25.99	34.2	12.8	21.4	28	21.5	28
Propithecus verreauxi	44.13	-23.51	24.2	14.5	65	28.61	33.8	11.7	22.1	27.2	20.7	27.2
Propithecus verreauxi	44.13	-21.83	25.4	14.1	64	26.21	34.5	12.8	21.7	28.1	21.6	28.1
Propithecus verreauxi	44.13	-21.77	25.4	14	64	26.26	34.5	12.9	21.6	28.1	21.6	28.1
Propithecus verreauxi	44.13	-21.73	25.3	14	65	26.1	34.3	12.8	21.5	28	21.5	28
Propithecus verreauxi	44.13	-21.71	25.3	14	65	25.95	34.2	12.8	21.4	28	21.5	28
Propithecus verreauxi	44.13	-21.7	25.3	13.9	64	26.24	34.2	12.7	21.5	28	21.4	28
Propithecus verreauxi	44.15	-20.79	25.3	12.5	62	24.98	33.3	13.4	19.9	27.9	21.7	27.9
Propithecus verreauxi	44.17	-22.64	19.1	13.9	68	23.44	28.3	7.9	20.4	21.6	15.7	21.6
Propithecus verreauxi	44.18	-22.1	24.6	14.8	66	25.82	34.2	11.9	22.3	27.2	20.8	27.2
Propithecus verreauxi	44.19	-22.68	20.7	14.6	67	24.44	30.3	8.8	21.5	23.2	17.1	23.2
Propithecus verreauxi	44.2	-21.62	25.1	14	64	26.05	34.1	12.5	21.6	27.7	21.2	27.7
Propithecus verreauxi	44.23	-24.22	24	13.9	64	28.59	33.5	12.1	21.4	27.1	20.6	27.1
Propithecus verreauxi	44.25	-21.63	24.8	14.1	64	25.85	33.9	12.2	21.7	27.3	20.9	27.3
Propithecus verreauxi	44.26	-21.83	24.8	14.5	65	25.88	34.3	12.1	22.2	27.4	21	27.4
Propithecus verreauxi	44.27	-21.37	24.9	13.7	64	25.73	33.8	12.5	21.3	27.5	21.2	27.5
Propithecus verreauxi	44.28	-21.28	25	13.6	63	25.91	33.8	12.4	21.4	27.6	21.2	27.6
Propithecus verreauxi	44.35	-20.58	25.5	12.7	62	24.62	33.5	13.3	20.2	27.7	21.9	27.9
Propithecus verreauxi	44.35	-20.43	25.4	12.2	62	24.35	33.3	13.7	19.6	27.7	21.9	27.9
Propithecus verreauxi	44.38	-20.45	25.5	12.4	62	24.2	33.4	13.5	19.9	27.7	22	27.9
Propithecus verreauxi	44.43	-20.17	25.6	12.2	62	23.5	33.3	13.9	19.4	27.8	22.2	28
Propithecus verreauxi	44.48	-21.33	24.6	14	63	25.68	33.9	12	21.9	27.1	20.8	27.1
Propithecus verreauxi	44.5	-24.33	24.5	14.9	65	29.93	34.4	11.8	22.6	27.7	21	27.7
Propithecus verreauxi	44.5	-22.28	24.7	15.7	66	25.66	35.2	11.5	23.7	27.2	21.8	27.3

Propithecus verreauxi	44.53	-19.23	26.2	12.2	64	20.08	34	15	19	27.9	23.1	28
Propithecus verreauxi	44.55	-20.12	25.9	12.8	63	23.06	33.9	13.7	20.2	27.9	22.5	28.2
Propithecus verreauxi	44.57	-25.09	24.1	12.9	62	30.53	33.5	12.7	20.8	27.5	20.7	27.5
Propithecus verreauxi	44.6	-19.97	26.1	12.9	64	22.32	34.1	14	20.1	28.2	22.7	28.2
Propithecus verreauxi	44.64	-23.63	25.3	16.5	66	30.32	35.9	11.1	24.8	28.2	21.6	28.2
Propithecus verreauxi	44.65	-25.05	24.4	13.3	61	31.4	34	12.5	21.5	27.8	20.9	27.8
Propithecus verreauxi	44.65	-23.64	25.2	16.5	66	30.24	35.9	11.1	24.8	28.2	21.6	28.2
Propithecus verreauxi	44.65	-20.07	26	13.3	64	22.62	34.2	13.5	20.7	27.9	22.7	28.1
Propithecus verreauxi	44.66	-20.07	26	13.3	63	22.52	34.3	13.5	20.8	27.8	22.7	28.1
Propithecus verreauxi	44.67	-22.76	23.8	16.8	66	26.74	35.3	10.1	25.2	26.3	20.9	26.5
Propithecus verreauxi	44.67	-21.29	24.5	14.3	63	25.98	34.1	11.7	22.4	27	20.7	27
Propithecus verreauxi	44.68	-20.08	26	13.4	64	22.48	34.4	13.5	20.9	27.8	22.7	28.1
Propithecus verreauxi	44.7	-20.58	25.8	14.1	62	24.04	35.1	12.7	22.4	28.1	22.3	28.1
Propithecus verreauxi	44.72	-22.33	23.2	15.5	66	24.7	33.7	10.5	23.2	25.6	19.5	25.7
Propithecus verreauxi	44.73	-22.82	21.9	15.5	66	25.28	32.3	9.1	23.2	24.3	18.1	24.4
Propithecus verreauxi	44.75	-21.39	24.7	14.4	63	26.18	34.5	11.8	22.7	27.2	20.8	27.3
Propithecus verreauxi	44.8	-20.42	26.1	14.4	62	23.59	35.8	12.8	23	28.3	22.7	28.3
Propithecus verreauxi	44.82	-21.38	25	14.5	63	26.7	34.9	12	22.9	27.5	21.1	27.6
Propithecus verreauxi	44.83	-22.68	22.8	15.8	66	25.06	33.5	9.9	23.6	25.2	19.9	25.4
Propithecus verreauxi	44.84	-22.46	22.4	15.2	67	23.98	32.5	9.9	22.6	24.7	18.7	24.8
Propithecus verreauxi	44.9	-21	25.4	14.4	63	25.68	35	12.3	22.7	27.7	21.6	27.8
Propithecus verreauxi	44.98	-21.91	25	15	63	27.28	35.5	11.9	23.6	27.5	20.9	27.7
Propithecus verreauxi	45.02	-22.02	24.8	15	64	26.65	35.2	11.8	23.4	27.3	20.8	27.5
Propithecus verreauxi	45.05	-19.75	26.8	13.9	64	21.05	35.8	14.1	21.7	28.5	23.5	28.7
Propithecus verreauxi	45.06	-21.98	25.1	15	63	27.14	35.5	12	23.5	27.6	21	27.8
Propithecus verreauxi	45.08	-21.63	25.7	14.8	61	28.84	36.3	12.3	24	28.3	21.5	28.6
Propithecus verreauxi	45.13	-22.58	22.8	14.8	66	23.67	32.6	10.5	22.1	25.1	19.2	25.1
Propithecus verreauxi	45.26	-22.44	22.9	14.4	66	23.51	32.4	10.8	21.6	25.2	19.4	25.2
Propithecus verreauxi	45.26	-22.37	23.1	14.4	66	23.94	32.8	11	21.8	25.4	19.5	25.5
Propithecus verreauxi	45.29	-22.32	23.5	14.5	65	24.42	33.3	11.2	22.1	25.8	19.8	25.9
Propithecus verreauxi	45.35	-21.45	25.5	14.5	61	27.78	35.8	12.4	23.4	27.9	21.4	28.2
Propithecus verreauxi	45.38	-22.48	21.8	13.6	67	22.18	30.8	10.6	20.2	24.1	18.6	24.1
Propithecus verreauxi	45.43	-21.43	25	14.4	62	27.27	35.3	12.2	23.1	27.5	21	27.7
Propithecus verreauxi	45.46	-21.4	25.6	14.4	62	27.37	35.8	12.7	23.1	28	21.5	28.2
Propithecus verreauxi	45.48	-21.43	25.7	14.4	62	27.47	35.9	12.7	23.2	28.2	21.6	28.4
Propithecus verreauxi	45.52	-21.4	25.5	14.4	62	27.23	35.6	12.6	23	27.9	21.4	28.1
Propithecus verreauxi	45.57	-21.35	23.8	14.1	63	26.01	33.8	11.5	22.3	26.2	19.9	26.3
Propithecus verreauxi	46.1	-24.55	24.2	15.2	66	29.84	34.3	11.5	22.8	27.5	21	27.5
Propithecus verreauxi	46.13	-24.8	24.2	14.9	67	29.78	34.1	11.9	22.2	27.6	20.9	27.6
Propithecus verreauxi	46.15	-24.73	23.5	14.8	67	29	33.3	11.3	22	26.7	20.3	26.7
Propithecus verreauxi	46.16	-24.76	23.7	14.9	67	29.2	33.6	11.5	22.1	26.9	20.5	26.9
Propithecus verreauxi	46.17	-24.76	23.6	14.8	66	29.2	33.5	11.4	22.1	26.8	20.4	26.8
Propithecus verreauxi	46.28	-24.98	24.3	14.8	66	29.59	34.1	12	22.1	27.6	20.9	27.6
Propithecus verreauxi	46.38	-25.03	24.5	14.7	65	29.65	34.3	12	22.3	27.8	22.5	27.8
Propithecus verreauxi	46.4	-25.13	24.4	13.8	65	29.38	33.8	12.8	21	27.8	22.3	27.8
Propithecus verreauxi	46.4	-24.87	24	14.9	67	28.78	33.9	11.8	22.1	27.3	20.7	27.3
Propithecus verreauxi	46.43	-25.07	24.4	14.3	65	29.54	34	12.2	21.8	27.7	22.3	27.7
Propithecus verreauxi	46.45	-24.69	23.2	14.2	66	28.22	32.8	11.5	21.3	26.4	20	26.4
Propithecus verreauxi	46.52	-25.05	21.9	12.3	65	26.24	30.5	11.7	18.8	24.9	20.1	24.9
Propithecus verreauxi	46.54	-24.83	23.8	13.8	66	27.75	33.2	12.3	20.9	27	20.6	27
Propithecus verreauxi	46.56	-24.97	23.9	13.4	66	27.55	32.9	12.6	20.3	27	21.9	27
Propithecus verreauxi	46.57	-20.52	16.7	12.5	65	24.05	25.6	6.4	19.2	19	13.1	19

Propithecus verreauxi	46.57	-20.5	16.6	12.5	65	24.05	25.5	6.3	19.2	18.9	13	18.9
Propithecus verreauxi	46.6	-24.91	21.3	12.1	65	25.58	29.7	11.3	18.4	24.2	18.2	24.2
Propithecus verreauxi	46.61	-24.82	23.5	13.1	65	27.03	32.5	12.4	20.1	26.5	21.7	26.6
Propithecus verreauxi	46.63	-24.97	22.8	12.5	65	26.35	31.5	12.3	19.2	25.8	20.9	25.8
Propithecus verreauxi	46.65	-25.17	22.5	11.4	64	25.6	30.5	12.8	17.7	25.4	20.4	25.4
Propithecus verreauxi	46.77	-24.93	23.1	11.3	63	25.24	31.1	13.3	17.8	25.9	21.2	26
Propithecus verreauxi	46.77	-24.63	21.7	12.1	65	25.8	30.1	11.5	18.6	24.6	18.6	24.6
Propithecus verreauxi	46.78	-25.08	22.5	10.7	62	24.72	30.3	13.3	17	25.4	20.6	25.4
Propithecus verreauxi	46.88	-24.9	23.1	10.2	61	24.23	30.7	14	16.7	25.9	21.3	25.9
Varecia rubra	49.58	-15.04	21	10.8	67	22.51	28.7	12.8	15.9	23.4	18.6	23.4
Varecia rubra	49.59	-15.19	24.1	8.9	64	20.51	31	17.1	13.9	26.4	23.2	26.4
Varecia rubra	49.61	-15.19	22.3	9.9	66	21.9	29.7	14.7	15	24.7	20	24.7
Varecia rubra	49.67	-15.38	23.4	8.7	63	20.98	30.3	16.6	13.7	25.8	22.4	25.8
Varecia rubra	49.75	-15.43	23.8	7.9	61	20.51	30.5	17.6	12.9	25.9	22.9	26.2
Varecia rubra	49.95	-15.43	21	9.2	63	21.63	28.2	13.8	14.4	23.5	20	23.5
Varecia rubra	50.01	-15.29	22.1	8.7	63	20.82	29.1	15.3	13.8	24.6	21.1	24.6
Varecia rubra	50.05	-15.27	21.2	9.1	64	21.36	28.3	14.1	14.2	23.6	20.1	23.6
Varecia rubra	50.18	-15.74	23.7	7.5	59	19.54	30.1	17.5	12.6	25.7	22.7	26
Varecia rubra	50.23	-15.78	24	7.3	59	19.33	30.2	17.9	12.3	25.9	23	26.3
Varecia rubra	50.23	-15.3	23.4	7.9	61	19.17	29.8	17	12.8	25.4	22.4	25.7
Varecia rubra	50.29	-15.27	22.9	8	62	19.31	29.4	16.5	12.9	24.9	21.9	25.2
Varecia rubra	50.43	-15.4	24.1	7.4	60	18.54	30.2	18	12.2	26	23.1	26.3
Varecia variegata	46.88	-22.57	21.3	11.2	63	25.66	29.3	11.6	17.7	23.8	18.2	24
Varecia variegata	47.34	-21.83	19	10.1	61	25.16	26.5	10.1	16.4	21.6	15.8	21.6
Varecia variegata	47.35	-21.78	18.7	10.1	60	25.16	26.4	9.8	16.6	21.3	15.5	21.3
Varecia variegata	47.36	-21.82	19	10.1	61	25.12	26.6	10.1	16.5	21.6	17.3	21.6
Varecia variegata	47.41	-21.74	19	10	60	25.26	26.6	10.2	16.4	21.7	17.3	21.7
Varecia variegata	47.41	-21.51	18.4	10.2	61	25.37	26.2	9.6	16.6	21	16.6	21
Varecia variegata	47.43	-21.29	18.5	10.4	61	25.57	26.5	9.5	17	21.2	16.8	21.2
Varecia variegata	47.46	-21.3	19.7	10.6	61	25.45	27.7	10.5	17.2	22.3	18	22.4
Varecia variegata	47.49	-21.59	18.9	10.1	61	25.35	26.6	10.1	16.5	21.6	17.2	21.6
Varecia variegata	47.49	-21.54	20.2	10.3	60	25.35	28	11.1	16.9	22.8	18.5	23
Varecia variegata	47.49	-21.39	20.3	10.6	61	25.5	28.3	11.1	17.2	22.9	18.5	23.1
Varecia variegata	47.63	-20.39	17	11	63	25.9	25.1	7.8	17.3	19.7	13.6	19.7
Varecia variegata	47.64	-20.39	17	10.9	62	26.05	25.2	7.8	17.4	19.7	13.6	19.7
Varecia variegata	47.72	-23.03	23.4	8.7	56	23.5	30.5	15.2	15.3	26.2	21.5	26.2
Varecia variegata	47.73	-23.03	23.4	8.7	56	23.5	30.5	15.2	15.3	26.2	21.5	26.2
Varecia variegata	47.95	-18.49	18.3	11.7	66	24.42	26.5	8.9	17.6	20.8	14.9	20.8
Varecia variegata	48.05	-20.84	21.4	9.7	61	24.16	28.8	13.1	15.7	24	19.4	24.1
Varecia variegata	48.12	-21.81	22.9	7.8	56	22.9	29.3	15.6	13.7	25.7	20.9	25.7
Varecia variegata	48.34	-19.19	19.1	10.7	64	25.69	27.2	10.6	16.6	22	16.9	22
Varecia variegata	48.43	-18.93	19	10.8	64	25.77	27.1	10.3	16.8	21.8	16.7	21.8
Varecia variegata	48.43	-18.79	19.5	11.1	64	26.34	27.7	10.4	17.3	22.3	17.2	22.3
Varecia variegata	48.46	-18.97	18.7	10.6	63	25.94	26.7	10	16.7	21.5	16.4	21.5
Varecia variegata	48.47	-18.97	18.2	10.6	63	26.15	26.3	9.7	16.6	21.1	15.9	21.1
Varecia variegata	48.5	-18.89	19.6	10.8	64	25.81	27.6	10.8	16.8	22.4	17.3	22.4
Varecia variegata	48.58	-18.2	19.1	11.2	65	25.32	27.2	10.1	17.1	21.7	16.7	21.7
Varecia variegata	48.6	-18.18	19.5	11.3	65	25.37	27.6	10.4	17.2	22.1	17.1	22.1
Varecia variegata	48.75	-18.08	19.8	10.8	65	25.2	27.6	11	16.6	22.5	17.3	22.5
Varecia variegata	48.77	-17.52	19.4	11.2	66	23.97	27.6	10.8	16.8	22	16.1	22
Varecia variegata	48.77	-16.94	19.3	11.1	66	23.89	27.5	10.7	16.8	21.8	17.2	22
Varecia variegata	48.78	-18.41	19.7	10.4	63	25.67	27.5	11.2	16.3	22.5	17.2	22.5

Varecia variegata	48.8	-16.28	18.8	11.4	66	23.55	27.2	10.1	17.1	21.2	16.5	21.3
Varecia variegata	48.81	-18.7	22.7	9.4	62	22.94	29.9	14.9	15	25.3	20.5	25.3
Varecia variegata	48.88	-18.2	17.9	10.3	64	25.29	25.6	9.6	16	20.6	15.4	20.6
Varecia variegata	48.97	-18.53	22.1	9.2	61	23.5	29.2	14.3	14.9	24.8	19.7	24.8
Varecia variegata	48.97	-16.95	19.7	10.5	65	23.98	27.6	11.6	16	22.4	17.4	22.4
Varecia variegata	48.98	-17.73	22	9.9	63	23.91	29.7	14	15.7	24.8	19.7	24.8
Varecia variegata	49	-18.37	22.8	9.1	61	23.18	29.8	15.1	14.7	25.5	20.5	25.5
Varecia variegata	49	-17.69	21.6	9.9	62	23.96	29.3	13.5	15.8	24.4	19.2	24.4
Varecia variegata	49.03	-17.3	22.8	9.8	65	22.12	30	15.1	14.9	25.4	20.7	25.4
Varecia variegata	49.05	-18.1	22.7	9.2	60	23.77	30	14.9	15.1	25.5	20.3	25.5
Varecia variegata	49.14	-15.41	20	11.7	69	22.28	28.3	11.4	16.9	22.3	16.8	22.3
Varecia variegata	49.15	-15.41	19.9	11.7	69	22.3	28.2	11.3	16.9	22.2	16.7	22.2
Varecia variegata	49.16	-15.49	19.6	11.6	68	22.55	27.9	11	16.9	21.9	16.3	21.9
Varecia variegata	49.16	-15.48	19.6	11.6	68	22.46	27.9	11	16.9	21.9	16.3	21.9
Varecia variegata	49.18	-17.83	22.3	8.9	58	24.05	29.9	14.8	15.1	25.2	19.8	25.2
Varecia variegata	49.22	-17.9	22.7	8.6	58	23.84	30.2	15.4	14.8	25.6	20.1	25.6
Varecia variegata	49.38	-15.68	20.8	10.5	67	22.3	28.6	13	15.6	23.2	18.6	23.2
Varecia variegata	49.44	-15.4	21.4	10.6	67	22.36	29.1	13.4	15.7	23.7	19.1	23.7
Varecia variegata	49.53	-15.44	22	10	66	21.98	29.4	14.4	15	24.4	19.7	24.4
Varecia variegata	49.53	-15.23	22.7	9.8	66	21.62	30	15.3	14.7	25.1	20.5	25.1
Varecia variegata	49.55	-15.29	21.9	10.2	67	21.88	29.4	14.2	15.2	24.3	19.6	24.3
Varecia variegata	49.57	-15.28	22.6	9.7	65	21.59	29.8	15.1	14.7	24.9	20.3	24.9
Varecia variegata	49.59	-15.22	22.9	9.6	66	21.4	30	15.6	14.4	25.2	22	25.2
Varecia variegata	49.73	-16.38	22.4	8.1	61	20.74	29	15.9	13.1	24.9	21.6	24.9
Varecia variegata	50.07	-15.28	21.2	9	63	21.09	28.4	14.2	14.2	23.7	20.2	23.7

V1	bio_1_AnnMeanTemp
V2	bio_2_MeanDiurnalTempRange
V3	bio_3_Isothermality
V4	bio_4_TempSeasonality (vworldclim value * 0.01)
V5	bio_5_MaxTempWarmestMonth
V6	bio_6_MinTempColdestMonth
V7	bio_7_TempAnnRange
V8	bio_8_MeanTempWettestQuart
V9	bio_9_MeanTempDriestQuart
V10	bio_10_MeanTempWarmQuart
V11	bio_11_MeanTempColdQuart
V12	bio_12_AnnPrecip
V13	bio_13_PrecipWettestMonth
V14	bio_14_PrecipDriestMonth
V15	bio_15_PrecipSeasonalityCV
V16	bio_16_PrecipWettestQuart
V17	bio_17_PrecipDriestQuart
V18	bio_18_PrecipWarmestQuart
V19	bio_19_PrecipColdestQuart
V20	Altitude

V11	V12	V13	V14	V15	V16	V17	V18	V19	V20
14.4	1492	294	29	80	811	118	811	125	1253
16.4	2075	363	59	65	1034	218	1034	261	884
19.8	1919	330	50	64	946	187	946	212	335
12.7	1541	304	31	79	835	117	835	117	1838
18.4	1688	310	40	71	861	155	861	164	733
19.7	2409	351	75	47	1025	275	1025	385	369
18.3	2343	324	72	42	927	274	927	406	603
18.1	834	168	17	79	459	60	459	67	494
17.5	1129	251	23	88	643	77	641	86	631
16.7	1448	356	5	106	929	17	929	17	1138
13.5	1577	301	39	76	812	125	812	134	1306
13.5	1577	301	39	76	812	125	812	134	1306
16.8	2018	410	46	76	1082	161	1041	207	666
13.3	1632	302	42	73	832	131	832	140	1374
13.3	1637	302	43	73	832	134	832	143	1372
14.8	1368	317	15	96	825	57	799	63	1266
14.4	1492	294	29	80	811	118	811	125	1253
16.4	2075	363	59	65	1034	218	1034	261	884
19.8	1919	330	50	64	946	187	946	212	335
12.7	1541	304	31	79	835	117	835	117	1838
12.7	1541	304	31	79	835	117	835	117	1838
18.4	1688	310	40	71	861	155	861	164	733
18.3	2343	324	72	42	927	274	927	406	603
18.1	834	168	17	79	459	60	459	67	494
17.5	1129	251	23	88	643	77	641	86	631
14.2	1636	319	40	76	841	129	841	140	1176
13.4	1569	298	39	76	809	124	809	132	1334
16.8	2018	410	46	76	1082	161	1041	207	666
13.3	1632	302	42	73	832	131	832	140	1374
13.3	1632	302	42	73	832	131	832	140	1374
11.9	1494	277	35	75	783	110	783	116	1707
14.2	1624	300	38	76	846	123	846	130	1275
14.8	1368	317	15	96	825	57	799	63	1266
18.4	1463	353	6	109	941	23	941	23	1020
14.4	1492	294	29	80	811	118	811	125	1253
16.4	2075	363	59	65	1034	218	1034	261	884
19.8	1919	330	50	64	946	187	946	212	335
23.2	1348	356	12	108	895	49	881	53	271
12.7	1541	304	31	79	835	117	835	117	1838
18.4	1688	310	40	71	861	155	861	164	733
19.7	2409	351	75	47	1025	275	1025	385	369
18.3	2343	324	72	42	927	274	927	406	603
20.7	434	112	3	103	289	13	289	13	56
22.6	914	292	1	126	664	3	489	3	47
20.1	512	110	6	88	309	26	309	34	216
19.4	735	194	4	106	487	16	419	17	561
24.2	1301	404	2	126	964	8	657	8	42
24.9	1458	448	1	123	1041	7	344	7	29
19.8	633	109	16	66	317	54	317	80	140
18.1	834	168	17	79	459	60	459	67	494
18.4	1463	353	6	109	941	23	941	23	1020

23.9	1633	415	14	107	1070	47	760	52	171
22.5	1265	340	2	110	850	12	380	12	233
18.7	790	165	13	83	446	48	446	56	365
13.7	1232	269	24	91	733	75	733	80	1391
14.2	1504	314	35	79	806	117	806	136	1183
13.5	1571	300	39	76	811	124	811	132	1311
23.9	1621	421	5	110	1084	15	453	15	83
24.2	1816	467	10	104	1155	33	769	33	81
23.2	1448	375	13	108	952	53	952	58	269
23.2	1348	356	12	108	895	49	881	53	271
23.3	1414	340	17	99	879	68	879	69	213
16.9	1663	303	41	70	846	155	846	164	1019
20.7	2806	384	81	42	1078	306	1057	504	138
18.1	2470	417	61	59	1172	257	1056	373	429
20	2530	408	83	49	1128	312	1128	454	22
16.1	1230	263	15	87	700	79	690	100	979
15.5	1376	324	18	96	831	75	828	79	1126
19.8	1919	330	50	64	946	187	946	212	335
16.8	1612	357	27	88	935	105	935	105	1045
16.7	1614	356	28	87	932	108	932	108	1061
16.3	1598	350	28	86	919	110	919	110	1106
16.3	1598	350	28	86	919	110	919	110	1107
19.9	2388	363	73	50	1058	280	1058	352	343
16.4	1600	350	28	86	919	110	919	110	1099
19	2147	337	65	54	983	239	983	297	536
21.3	3036	496	91	45	1260	360	1260	543	9
17.7	1773	340	42	73	939	154	939	175	815
13.3	1544	305	31	79	838	116	838	116	1726
15.3	1635	322	36	76	874	136	874	136	1319
16.7	1650	320	35	77	881	135	881	135	1073
18.1	1935	352	48	68	986	177	986	206	720
15.5	1652	324	37	76	880	140	880	140	1266
13.7	1568	309	33	78	846	123	846	123	1626
17.2	1689	325	37	76	896	143	896	144	959
15.7	1663	323	38	75	880	145	880	145	1238
17.8	1866	344	49	69	949	180	949	199	774
18.9	2154	352	57	60	1023	216	1023	272	548
19.7	2316	364	61	57	1068	237	1068	306	397
18.8	2146	353	58	60	1020	217	1020	271	575
19.5	2346	361	62	55	1062	248	1062	323	414
17.7	1968	344	56	63	964	201	964	234	773
19.2	2284	352	62	55	1035	246	1035	317	468
16.9	1663	303	41	70	846	155	846	164	1019
21.4	2988	418	74	46	1188	295	1182	491	9
16	1622	297	39	71	832	148	832	153	1196
19.7	2409	351	75	47	1025	275	1025	385	369
14.8	1595	286	40	69	805	152	805	158	1427
19.3	1836	305	61	57	848	223	848	259	493
17.8	1738	294	53	61	824	196	824	221	799
20.7	2806	384	81	42	1078	306	1057	504	138
18.2	2232	309	68	45	910	262	910	373	640
18.3	2247	310	69	44	907	265	907	380	622

22.4	1770	257	76	40	679	240	679	370	10
21.1	2744	371	84	40	1040	311	1003	513	101
21.3	2745	371	85	40	1039	311	998	518	60
20.8	2513	328	76	39	940	294	907	472	180
21.6	2744	369	85	39	1036	312	988	523	18
20.2	2383	312	74	38	887	285	870	446	296
21.6	2507	325	79	38	931	298	879	486	55
14.7	1179	263	23	91	702	72	702	76	1216
16	1182	263	25	89	685	81	685	86	931
16.5	1223	269	27	88	697	87	697	94	829
16.1	1221	269	27	88	701	86	701	91	914
17.4	1682	314	40	77	901	148	867	171	586
17.3	1727	317	42	76	918	156	879	182	600
18	1867	354	53	75	1002	172	916	209	479
20.2	2380	340	81	45	1001	314	1001	413	23
20.2	2382	339	82	44	998	316	998	414	21
14.2	1089	229	17	90	643	57	643	57	1470
12.2	1215	209	36	65	605	125	605	132	1779
13.9	1157	203	34	66	579	117	579	125	1439
16	1068	194	32	67	540	105	540	115	964
14.9	1122	200	33	66	564	112	564	121	1214
19.6	1242	187	46	49	540	165	500	225	112
18	1071	192	35	64	526	110	526	127	456
19.1	1113	162	45	42	449	157	449	216	227
19.4	1392	222	47	55	632	172	604	213	110
16.7	1131	194	38	59	537	129	537	146	762
17.5	1164	194	41	56	534	141	534	163	572
17.9	1220	202	43	56	555	148	555	172	466
19.8	1611	257	60	47	700	223	615	308	25
19.5	1813	284	60	53	808	231	771	276	61
19.7	1810	251	74	37	706	275	706	381	4
13.7	1232	269	24	91	733	75	733	80	1391
12.7	1280	271	24	90	761	76	761	82	1599
16.1	1653	318	36	75	869	141	869	160	739
19.6	2223	328	80	44	915	319	843	406	18
18.8	2123	308	75	46	891	296	839	364	186
19.6	2743	414	89	48	1154	374	1069	456	35
24.2	1645	420	14	108	1081	47	766	52	138
24	1629	414	14	108	1067	47	758	52	167
24.2	1641	415	14	107	1072	48	762	53	123
18.4	1353	310	16	95	812	65	812	70	1048
22.4	1567	365	15	101	981	55	722	61	376
23.3	1414	340	17	99	879	68	879	69	213
24.7	1138	309	12	108	755	51	747	57	67
24	1362	316	21	93	821	81	821	84	101
23.7	1370	305	23	89	806	88	806	91	136
20.3	1421	278	23	83	793	95	768	95	639
21.8	1435	279	26	80	789	106	789	106	385
22.4	1397	265	28	77	753	113	753	117	283
23.8	1359	236	40	61	648	150	648	164	19
22.2	1376	238	38	61	656	152	656	164	261
23.4	1388	244	45	53	601	174	601	197	6

21.7	1676	249	68	41	674	242	674	305	161
25	1505	456	1	123	1086	6	374	6	32
24.8	1489	457	1	124	1070	5	803	5	13
24.8	1489	457	1	124	1069	5	803	5	12
24.2	1492	438	1	119	1033	6	391	6	172
24.5	1484	436	1	119	1031	6	383	6	112
17.5	1129	251	23	88	643	77	641	86	631
24.4	1502	446	1	120	1059	6	373	6	114
24.2	1505	448	1	121	1063	5	375	5	150
24.1	1518	450	1	121	1065	5	388	5	218
25.1	1649	453	1	117	1137	7	439	7	141
24	1476	449	1	124	1074	5	780	5	13
24.2	1541	455	1	120	1087	5	390	5	205
24.4	1536	453	1	120	1085	5	386	5	166
14.2	1404	355	5	106	901	18	847	18	1582
23.9	1531	457	1	122	1099	4	807	4	176
23.8	1542	475	1	125	1126	6	817	6	40
23.1	1549	467	1	123	1106	4	386	4	198
23.7	1539	453	1	120	1076	5	397	5	140
14.2	1624	300	38	76	846	123	846	130	1275
14.2	1624	300	38	76	846	123	846	130	1275
12.7	1424	270	30	80	770	94	770	100	1616
24.1	1589	434	2	113	1074	7	468	7	197
13.6	1539	287	35	78	819	111	819	118	1394
13.7	1551	288	35	78	822	113	822	121	1377
24.7	1882	482	13	101	1177	45	772	45	45
14.4	1313	311	13	99	802	49	779	55	1353
23.9	1621	421	5	110	1084	15	453	15	83
23.3	1638	428	4	111	1100	14	849	14	176
23.4	1637	427	5	111	1098	15	848	15	177
18	2217	376	52	64	1074	206	1036	299	635
16.8	1344	368	4	114	905	19	886	22	1145
17.7	2269	377	54	60	1076	222	1076	330	663
16	1763	321	49	70	899	168	893	224	937
15.1	1437	331	10	103	897	33	897	37	1619
19.8	1613	381	9	103	1013	33	1001	35	803
17.6	2369	397	60	61	1142	248	1142	355	674
15.5	1807	324	51	68	903	179	900	244	1012
15.8	1778	322	49	70	909	169	909	195	993
16.8	1462	338	9	104	917	30	917	33	1318
15.2	1832	324	52	66	905	184	903	252	1067
14.7	1780	314	51	67	887	175	880	240	1156
16.3	1869	334	52	70	962	179	929	207	928
16	1992	351	56	65	987	208	987	286	937
16.6	1398	326	8	106	892	27	892	27	1294
17.4	2599	442	72	62	1280	291	1280	354	711
15.4	1418	280	24	83	790	104	790	107	1108
15.8	1430	281	24	82	797	105	797	108	1034
17	1172	262	9	98	715	49	709	60	866
17.1	1417	321	9	103	893	30	893	36	1209
16.1	1476	282	27	78	806	117	806	126	977
16.1	1220	254	14	89	703	77	703	77	1025

19.6	2586	461	71	57	1232	306	1232	386	341
19	2478	435	69	58	1192	285	1192	356	450
20.9	1654	365	16	98	1011	49	980	55	547
20.2	1609	352	15	97	979	47	956	53	668
20.7	1634	359	15	97	996	48	968	55	585
21	2676	491	73	55	1255	317	1255	425	92
20.8	2739	500	74	54	1264	323	1264	448	109
15.7	1450	307	14	95	870	50	870	61	1419
21.3	2744	506	75	54	1263	324	1263	456	24
18.8	1825	317	45	65	912	173	912	201	535
19.6	2479	418	72	54	1145	292	1145	371	362
18.3	1742	308	40	67	880	160	880	186	622
19.9	1998	336	55	61	963	203	963	234	305
19.2	2219	353	66	55	1028	248	1028	310	497
24.4	1723	459	4	109	1124	16	709	16	179
24.7	1715	452	4	109	1121	14	707	14	135
24.7	1938	470	19	95	1178	68	788	68	25
24.9	1755	461	6	107	1134	20	728	20	66
24.7	1866	485	11	103	1176	38	770	38	36
25	1815	479	8	105	1157	28	753	28	7
25.1	1711	444	6	107	1112	19	717	19	10
24.5	1822	473	9	104	1159	32	767	32	43
23.4	1805	464	9	104	1151	31	762	31	222
24.2	1816	467	10	104	1155	33	769	33	81
22.5	1679	431	6	109	1104	19	729	19	350
23.2	1716	438	7	106	1115	24	740	24	233
22.9	1951	450	21	95	1183	75	1145	75	320
24.3	2105	467	28	89	1239	101	1191	101	65
24.2	2105	506	23	94	1277	85	864	85	15
23.9	2103	492	24	93	1266	88	863	88	82
17.9	1447	345	6	108	928	24	928	24	1095
15.1	1437	331	10	103	897	33	897	37	1619
19.8	1613	381	9	103	1013	33	1001	35	803
16.8	1462	338	9	104	917	30	917	33	1318
21.1	1720	411	11	102	1079	40	1056	40	576
23.7	2053	495	21	96	1257	78	856	78	74
24.4	2057	478	24	94	1247	88	1215	88	6
23	1942	454	19	98	1198	69	832	69	259
23.6	2004	469	21	96	1229	77	855	77	143
21.4	1717	389	15	98	1060	49	1027	53	489
24.5	1935	464	19	100	1212	65	905	68	12
20.9	1654	365	16	98	1011	49	980	55	547
24.7	1912	467	17	103	1215	59	895	62	15
20.2	1609	352	15	97	979	47	956	53	668
13.4	1569	298	39	76	809	124	809	132	1334
14	1643	317	41	76	840	132	840	144	1213
13.3	1626	301	42	74	829	131	829	139	1381
13.3	1632	302	42	73	832	131	832	140	1374
13.3	1637	302	43	73	832	134	832	143	1372
12.2	1493	278	34	76	785	108	785	113	1657
12.2	1493	278	34	76	785	108	785	113	1657
11.9	1494	277	35	75	783	110	783	116	1707

14.2	1624	300	38	76	846	123	846	130	1275
20.7	434	112	3	103	289	13	289	13	56
21.5	651	194	1	126	487	5	487	5	20
21.4	693	190	1	119	498	6	498	6	66
19.7	490	122	4	100	319	17	319	17	280
21.8	713	241	0	134	545	2	545	2	4
22.9	1076	343	3	127	804	12	804	12	7
21.7	730	243	1	133	553	3	553	3	31
23.1	1128	350	3	124	826	12	826	12	37
21.5	756	205	2	113	528	9	528	9	102
21.6	742	198	2	113	516	9	516	9	74
21.6	746	201	2	113	520	9	520	9	75
21.5	757	206	2	113	529	9	529	9	102
21.4	761	207	2	114	532	9	532	9	108
15.7	800	201	5	106	532	17	532	17	1325
20.8	784	204	3	109	534	12	534	12	290
20.9	823	228	2	115	574	9	574	9	259
21.2	827	246	1	122	593	5	593	5	199
21.9	781	267	0	133	592	2	588	2	49
21.9	780	277	0	136	605	1	594	1	28
22	787	275	0	136	606	2	595	2	38
22.2	825	289	0	136	633	2	622	2	12
20.8	877	253	2	118	622	7	495	7	329
20.8	737	200	3	109	504	14	422	15	385
23.1	1049	314	2	119	753	9	578	9	31
22.5	865	288	1	129	645	3	637	3	17
22.7	917	295	1	126	667	3	491	3	21
23	1216	340	2	113	835	12	657	12	108
22.7	913	290	1	125	662	3	487	3	47
22.7	920	291	1	126	664	3	492	3	56
19.8	728	194	4	107	486	16	414	17	483
22.7	925	290	1	124	665	3	494	3	65
24	1293	409	1	129	954	7	614	7	50
20.6	893	251	2	117	635	7	508	7	407
22.3	873	268	1	124	631	4	470	4	115
23.4	1320	392	2	123	950	9	637	9	165
18.1	753	192	5	105	494	19	435	19	862
23.7	1320	390	2	123	949	9	637	9	94
24.2	1232	385	1	132	938	7	589	7	47
22.5	1265	340	2	110	850	12	380	12	233
22.7	907	271	1	122	648	4	486	4	94
23.1	1185	333	2	113	809	11	347	11	98
19.1	742	192	5	106	489	18	430	19	696
22.8	1016	299	2	120	713	6	546	6	95
22.1	1255	339	2	110	844	11	381	11	322
18.7	750	193	6	106	496	22	444	22	807
21.6	942	264	2	118	675	7	524	7	256
23.5	1136	328	2	116	774	9	326	9	35
20.7	753	199	3	109	512	13	437	13	408
23.6	1291	349	3	108	845	14	388	14	73
19.2	754	192	4	104	493	14	432	14	703
24.3	1279	400	1	128	957	7	643	7	28

19.4	765	197	3	105	504	11	438	11	667
19.5	765	199	3	106	508	12	441	12	631
19.8	761	200	3	107	509	11	442	11	572
24.5	1263	409	1	130	959	6	643	6	2
23.8	1348	411	2	123	977	9	689	9	121
18.6	792	201	2	103	517	9	517	9	841
20.7	967	260	3	114	679	12	562	12	385
21	985	263	4	113	689	14	567	14	314
23.1	1528	442	2	113	1022	12	414	13	362
24.7	1396	438	1	125	1013	6	313	6	50
24.7	1425	442	1	124	1029	7	326	7	41
24.9	1413	441	1	125	1022	6	321	6	23
24.9	1410	440	1	125	1020	6	320	6	13
24.9	1409	440	1	125	1018	6	320	6	15
24.9	1418	442	1	124	1023	6	324	6	20
23.9	1565	463	2	116	1057	10	428	12	255
24.9	1422	442	1	125	1026	6	326	6	17
24.9	1422	442	1	124	1025	6	326	6	22
24.3	1573	469	2	116	1070	10	425	12	186
24.5	1571	470	2	117	1073	10	422	12	145
24.5	1516	458	1	121	1077	7	372	8	114
24.8	1472	450	1	123	1048	7	352	7	38
25	1480	452	1	124	1073	5	649	5	72
24.8	1511	450	1	120	1055	7	390	7	64
24.5	1487	443	2	120	1035	8	379	8	79
13.5	1149	259	17	95	707	55	707	60	1481
15.2	1113	250	21	91	661	69	661	72	1117
15.2	1148	253	23	90	673	76	673	79	1107
12.6	1271	262	24	88	746	79	746	84	1643
16.1	1154	253	24	87	661	81	661	88	910
15.6	1176	255	24	87	677	82	677	88	1018
17.2	1186	259	26	87	670	86	666	96	698
14.7	1179	263	23	91	702	72	702	76	1216
11.6	1377	270	30	82	757	93	757	94	1742
13.2	1323	274	29	82	727	104	727	111	1411
14.7	1462	320	32	84	827	109	827	116	1102
15.3	1520	316	37	81	833	123	833	133	986
15.1	1508	317	36	82	833	121	833	130	1034
15.4	1557	321	38	80	846	128	846	141	973
15.4	1626	332	40	78	872	134	872	153	953
14.7	1570	332	37	80	858	124	858	140	1081
17	1952	396	48	77	1051	157	1015	195	627
14.8	1631	343	39	79	870	127	870	151	1052
14.8	1631	343	39	79	870	127	870	151	1052
13.5	1571	300	39	76	811	124	811	132	1311
13.5	1577	301	39	76	812	125	812	134	1306
17.1	1973	395	49	76	1059	162	1018	202	609
16.1	1895	394	44	77	1012	149	988	188	801
14.3	1617	330	40	77	845	130	845	158	1141
14.4	1663	332	42	75	854	134	850	165	1134
14	1643	317	41	76	840	132	840	144	1213
15.3	1731	350	44	76	912	143	912	172	948

16.6	1948	386	49	75	1034	162	999	204	690
16.7	2025	408	47	76	1081	164	1040	211	672
16.7	2100	420	47	74	1117	171	1066	225	660
16.9	2159	435	46	75	1151	174	1090	234	639
16.6	2112	428	45	75	1123	170	1067	227	687
16.8	2157	433	46	75	1147	175	1087	235	648
16.5	2102	426	45	75	1116	169	1061	227	704
17.1	2217	445	46	75	1182	180	1113	243	598
14.3	1709	338	43	74	873	142	868	180	1137
17.1	2278	487	39	76	1220	182	1103	260	621
13.5	1652	304	43	73	839	135	839	145	1345
17.1	2322	429	54	69	1199	209	1113	287	571
20.4	2462	381	80	47	1057	305	1057	411	4
20.3	2557	417	88	50	1131	303	1131	434	4
20.1	2689	426	88	47	1148	323	1148	501	1
17.6	2369	397	60	61	1142	248	1142	355	674
15.5	1807	324	51	68	903	179	900	244	1012
16.1	1476	282	27	78	806	117	806	126	977
23.2	1448	375	13	108	952	53	952	58	269
24.2	1645	420	14	108	1081	47	766	52	138
24	1629	414	14	108	1067	47	758	52	167
24.1	1612	409	14	107	1053	49	748	54	142
24.2	1641	415	14	107	1072	48	762	53	123
18.4	1353	310	16	95	812	65	812	70	1048
22.4	1567	365	15	101	981	55	722	61	376
23.3	1414	340	17	99	879	68	879	69	213
23.4	1368	306	22	90	809	85	809	89	181
20.3	1421	278	23	83	793	95	768	95	639
21.8	1435	279	26	80	789	106	789	106	385
21.8	1435	279	26	80	789	106	789	106	385
17.5	1129	251	23	88	643	77	641	86	631
16.7	1448	356	5	106	929	17	929	17	1138
16.8	2018	410	46	76	1082	161	1041	207	666
14	1643	317	41	76	840	132	840	144	1213
13.3	1632	302	42	73	832	131	832	140	1374
13.4	1645	303	43	73	835	136	835	145	1362
12.7	1606	294	42	73	818	133	818	142	1492
12.2	1493	278	34	76	785	108	785	113	1657
11.9	1494	277	35	75	783	110	783	116	1707
14.8	1368	317	15	96	825	57	799	63	1266
14.4	1492	294	29	80	811	118	811	125	1253
16.4	2075	363	59	65	1034	218	1034	261	884
19.8	1919	330	50	64	946	187	946	212	335
12.7	1541	304	31	79	835	117	835	117	1838
18.4	1688	310	40	71	861	155	861	164	733
19.7	2409	351	75	47	1025	275	1025	385	369
18.3	2343	324	72	42	927	274	927	406	603
17.5	1129	251	23	88	643	77	641	86	631
16	1182	263	25	89	685	81	685	86	931
16.1	1221	269	27	88	701	86	701	91	914
13.8	1238	269	24	90	735	76	735	81	1387
17.4	1711	315	46	76	921	154	860	185	590

12.8	1484	287	28	79	793	92	793	92	1485
16.8	2018	410	46	76	1082	161	1041	207	666
19.6	2586	461	71	57	1232	306	1232	386	341
14.3	1561	284	35	71	811	142	811	164	1309
14.4	1313	311	13	99	802	49	779	55	1353
17.7	2184	371	49	66	1068	196	1068	280	728
15.7	1928	334	50	67	959	183	940	253	975
16	1763	321	49	70	899	168	893	224	937
16	1626	316	33	80	886	128	863	136	955
17.6	2369	397	60	61	1142	248	1142	355	674
15.5	1807	324	51	68	903	179	900	244	1012
15.5	1807	324	51	68	903	179	900	244	1012
15.8	1778	322	49	70	909	169	909	195	993
15.6	1889	334	53	66	930	192	930	265	985
15.2	1832	324	52	66	905	184	903	252	1067
15.3	1799	322	51	68	901	177	901	210	1052
14.7	1780	314	51	67	887	175	880	240	1156
16.3	1869	334	52	70	962	179	929	207	928
16	1992	351	56	65	987	208	987	286	937
17.3	2578	436	71	62	1270	287	1270	350	717
15.4	1418	280	24	83	790	104	790	107	1108
15.8	1430	281	24	82	797	105	797	108	1034
16	1245	261	15	88	718	81	718	81	1045
16.1	1220	254	14	89	703	77	703	77	1025
16.1	1230	263	15	87	700	79	690	100	979
16.1	1819	319	48	68	928	177	928	209	972
15.5	1376	324	18	96	831	75	828	79	1126
21	2676	491	73	55	1255	317	1255	425	92
20.8	2739	500	74	54	1264	323	1264	448	109
21.3	2744	506	75	54	1263	324	1263	456	24
18.8	2481	423	71	55	1156	294	1156	374	493
16.5	1436	279	27	76	763	116	763	133	875
18.8	1825	317	45	65	912	173	912	201	535
19.6	2479	418	72	54	1145	292	1145	371	362
18.3	1742	308	40	67	880	160	880	186	622
19.9	1998	336	55	61	963	203	963	234	305
19.2	2219	353	66	55	1028	248	1028	310	497
16.8	1612	357	27	88	935	105	935	105	1045
16.7	1614	356	28	87	932	108	932	108	1061
16.3	1598	350	28	86	919	110	919	110	1106
16.3	1598	350	28	86	919	110	919	110	1107
19.9	2388	363	73	50	1058	280	1058	352	343
16.4	1600	350	28	86	919	110	919	110	1099
19	2147	337	65	54	983	239	983	297	536
19.4	2332	355	72	50	1034	273	1034	345	460
19.4	2332	355	72	50	1034	273	1034	345	460
21.3	3036	496	91	45	1260	360	1260	543	9
17.7	1773	340	42	73	939	154	939	175	815
13.3	1544	305	31	79	838	116	838	116	1726
18.1	1935	352	48	68	986	177	986	206	720
15.5	1652	324	37	76	880	140	880	140	1266
17.8	1866	344	49	69	949	180	949	199	774

18.9	2154	352	57	60	1023	216	1023	272	548
19.7	2316	364	61	57	1068	237	1068	306	397
18.8	2146	353	58	60	1020	217	1020	271	575
19.5	2346	361	62	55	1062	248	1062	323	414
19.8	2432	365	63	53	1077	254	1077	347	340
18.9	2137	338	59	56	993	228	993	288	534
21.5	2880	407	72	47	1166	287	1166	461	12
21.1	3047	422	73	46	1210	296	1191	505	51
19.7	2409	351	75	47	1025	275	1025	385	369
21.8	2780	399	88	43	1102	314	1102	484	8
21	477	138	1	116	340	8	313	9	23
20.9	487	158	0	128	371	4	341	4	50
20.7	434	112	3	103	289	13	289	13	56
21	381	87	4	91	238	19	238	21	3
20.8	341	76	4	88	206	17	206	18	6
21	414	94	5	90	257	21	257	25	4
20.9	416	94	5	90	258	21	258	25	1
21	416	95	5	91	259	21	259	25	2
20.9	417	95	5	91	259	21	259	25	19
21	378	89	4	92	240	18	240	20	15
20.8	424	96	5	90	263	21	263	25	27
20.8	426	97	5	91	265	21	265	25	43
21.5	658	198	1	128	493	4	493	4	25
20.8	434	95	6	87	264	24	264	29	16
20.5	444	98	6	87	271	24	271	29	91
20.5	444	99	5	88	272	23	272	27	92
20.4	400	98	4	95	258	17	258	17	128
20.7	451	106	5	94	285	21	285	24	86
20.5	418	103	4	97	270	17	270	18	120
21	739	187	2	111	508	10	508	10	143
19.7	490	122	4	100	319	17	319	17	280
20.4	446	95	6	86	268	25	268	31	82
20.3	450	97	6	87	271	25	271	31	93
20.3	489	123	4	100	320	16	320	17	167
21.8	713	241	0	134	545	2	545	2	4
20.2	454	96	6	86	271	26	271	33	118
20.3	454	96	6	86	271	26	230	33	100
20.5	476	120	4	100	311	17	311	18	118
21.6	735	240	1	130	553	3	553	3	55
21.7	730	243	1	133	553	3	553	3	31
20.1	460	97	7	85	275	27	233	34	142
19.7	524	133	4	101	344	17	344	17	306
21.6	737	240	1	130	554	3	554	3	59
21.4	755	206	2	116	530	9	530	9	111
21.6	740	243	1	131	557	3	557	3	58
21.4	762	209	2	115	535	9	535	9	114
20	526	134	4	102	345	17	345	18	250
21.6	742	198	2	113	516	9	516	9	74
21.5	755	205	2	115	528	9	528	9	97
21.5	757	206	2	113	529	9	529	9	102
21.4	761	207	2	114	532	9	532	9	108
21.7	749	249	1	132	565	3	565	3	56

15.7	800	201	5	106	532	17	532	17	1325
20.8	784	204	3	109	534	12	534	12	290
17.1	759	193	5	105	505	18	505	18	1029
21.4	787	244	1	125	578	4	578	4	139
19.9	537	129	5	97	342	21	342	25	289
21.6	774	254	1	130	579	3	579	3	106
20.2	620	159	4	103	408	18	408	20	268
20.2	567	135	5	95	359	22	359	27	246
20.8	737	200	3	109	504	14	422	15	385
20	437	87	8	81	250	30	250	40	49
20.7	631	156	5	100	405	20	405	23	129
19.8	728	194	4	107	486	16	414	17	483
18.1	753	192	5	105	494	19	435	19	862
19.1	742	192	5	106	489	18	430	19	696
18.7	750	193	6	106	496	22	444	22	807
20.3	500	105	7	87	300	26	300	37	135
20.8	769	204	3	110	530	12	446	12	380
21.1	772	203	2	112	537	11	445	11	297
21.5	785	205	2	113	550	10	447	10	214
19.4	765	197	3	105	504	11	438	11	667
19.5	765	199	3	106	508	12	441	12	631
19.8	761	200	3	107	509	11	442	11	572
18.5	792	207	3	106	528	10	463	10	851
19.7	437	88	12	69	228	40	228	60	137
18.6	787	197	3	103	508	10	508	10	841
18.6	792	201	2	103	517	9	517	9	841
21.3	1050	279	4	114	737	14	597	14	296
20.9	990	265	4	113	693	14	572	14	339
17.5	794	215	3	107	525	10	525	10	988
19.8	563	106	10	78	313	33	313	54	208
20	591	108	12	73	317	40	317	63	81
19.7	619	118	11	77	339	39	339	57	182
19.2	563	88	17	55	260	62	260	99	126
20	587	105	13	71	308	43	308	67	81
20	683	138	11	82	383	38	383	53	124
19.3	646	127	11	80	359	39	359	55	307
19.3	646	127	11	80	359	39	359	55	307
19.4	628	119	12	76	341	42	341	60	251
19.5	619	115	12	74	331	43	331	63	220
20.1	581	99	14	66	293	48	293	75	53
20.2	561	90	16	58	264	57	264	88	27
20.4	581	87	19	52	257	67	257	103	20
20.1	682	112	19	61	324	66	324	96	74
18.7	790	165	13	83	446	48	446	56	365
19.3	767	142	19	71	395	63	395	82	242
19.8	731	121	22	60	344	75	344	104	124
19.1	802	146	22	68	405	71	405	91	271
18.2	860	225	11	101	540	35	540	35	608
18.3	777	124	28	53	351	95	351	122	466
20	838	137	26	58	383	92	383	124	78
20.1	800	123	28	51	342	97	339	139	80
17.7	879	148	30	58	415	99	415	120	594

19.7	951	155	31	56	428	109	426	144	119
19.2	891	140	32	51	386	111	386	149	259
18.9	864	127	35	42	349	119	340	165	307
20.1	904	128	38	39	350	130	350	191	44
16.4	1010	191	29	71	529	90	529	97	883
17.1	934	229	17	96	564	52	564	55	756
19.1	1113	162	45	42	449	157	449	216	227
20.1	1327	194	52	43	549	189	549	267	7
14	1110	257	16	97	688	51	688	55	1385
14	1110	257	16	97	688	51	688	55	1385
13.9	1113	257	16	97	690	51	690	55	1401
13.9	1113	257	16	97	690	51	690	55	1401
12.2	1216	271	16	94	751	55	751	60	1750
12.2	1216	271	16	94	751	55	751	60	1750
12.8	1177	262	16	95	729	53	729	58	1626
12.8	1177	262	16	95	729	53	729	58	1626
11	1283	285	17	93	784	60	784	66	1992
13.5	1149	259	17	95	707	55	707	60	1481
10.4	1308	287	19	91	791	65	791	70	2113
10.4	1312	287	19	91	792	66	792	72	2098
9	1362	290	21	88	804	75	804	80	2371
20	1402	196	58	39	553	211	553	295	0.001
15.9	1085	263	15	98	669	50	669	50	955
13.2	1323	274	29	82	727	104	727	111	1411
25	1815	479	8	105	1157	28	753	28	7
24.2	1816	467	10	104	1155	33	769	33	81
24.3	2105	467	28	89	1239	101	1191	101	65
23.6	2004	469	21	96	1229	77	855	77	143
16.1	1819	319	48	68	928	177	928	209	972
23.3	1414	340	17	99	879	68	879	69	213
23	1216	340	2	113	835	12	657	12	108
24.2	1301	404	2	126	964	8	657	8	42
24.5	1487	443	2	120	1035	8	379	8	79
24.2	1492	438	1	119	1033	6	391	6	172
24.5	1484	436	1	119	1031	6	383	6	112
20.8	426	97	5	91	265	21	265	25	43
20	591	108	12	73	317	40	317	63	81
19.8	633	109	16	66	317	54	317	80	140
18.7	790	165	13	83	446	48	446	56	365
18.1	834	168	17	79	459	60	459	67	494
13.7	1232	269	24	91	733	75	733	80	1391
14.2	1504	314	35	79	806	117	806	136	1183
14.2	1504	314	35	79	806	117	806	136	1183
12.8	1484	287	28	79	793	92	793	92	1485
13.5	1571	300	39	76	811	124	811	132	1311
16.8	2018	410	46	76	1082	161	1041	207	666
18.1	834	168	17	79	459	60	459	67	494
17.5	1129	251	23	88	643	77	641	86	631
13.5	1577	301	39	76	812	125	812	134	1306
13.4	1569	298	39	76	809	124	809	132	1334
11.9	1494	277	35	75	783	110	783	116	1707
13.7	1551	288	35	78	822	113	822	121	1377

14.8	1368	317	15	96	825	57	799	63	1266
14.8	1368	317	15	96	825	57	799	63	1266
15.8	1778	322	49	70	909	169	909	195	993
14.4	1492	294	29	80	811	118	811	125	1253
16.4	2075	363	59	65	1034	218	1034	261	884
19.8	1919	330	50	64	946	187	946	212	335
19.8	1919	330	50	64	946	187	946	212	335
12.7	1541	304	31	79	835	117	835	117	1838
18.4	1688	310	40	71	861	155	861	164	733
18.4	1688	310	40	71	861	155	861	164	733
19.7	2409	351	75	47	1025	275	1025	385	369
18.3	2343	324	72	42	927	274	927	406	603
18.3	2343	324	72	42	927	274	927	406	603
21.6	735	240	1	130	553	3	553	3	55
21.4	761	207	2	114	532	9	532	9	108
22.6	914	292	1	126	664	3	489	3	47
19.4	735	194	4	106	487	16	419	17	561
23	1198	332	2	113	817	11	354	11	123
24.9	1410	440	1	125	1020	6	320	6	13
20.7	434	112	3	103	289	13	289	13	56
21	463	140	1	119	337	8	310	8	5
20.9	487	158	0	128	371	4	341	4	50
20.6	492	131	2	108	339	11	339	11	111
20.7	434	112	3	103	289	13	289	13	56
20.6	455	115	3	105	303	13	303	13	90
20.8	341	76	4	88	206	17	206	18	6
21	416	95	5	91	259	21	259	25	2
20.8	424	96	5	90	263	21	263	25	27
20.5	444	98	6	87	271	24	271	29	91
20.7	451	106	5	94	285	21	285	24	86
20.2	454	96	6	86	271	26	271	33	118
20.5	630	158	5	101	408	20	408	23	193
20.6	630	156	5	100	406	20	406	23	145
20.7	631	156	5	100	405	20	405	23	129
20.7	632	156	5	101	406	20	406	23	137
19.5	641	143	6	94	403	22	403	33	312
19.8	633	109	16	66	317	54	317	80	140
20.4	581	87	19	52	257	67	257	103	20
20.1	649	106	18	61	310	62	310	91	81
20.1	1327	194	52	43	549	189	549	267	7
23.1	1011	309	2	121	736	9	559	9	9
22.5	1265	340	2	110	850	12	380	12	233
23.1	1185	333	2	113	809	11	347	11	98
22.9	1201	334	2	112	817	11	355	11	154
23.8	1348	411	2	123	977	9	689	9	121
24.2	1492	438	1	119	1033	6	391	6	172
24.2	1501	441	1	119	1042	6	390	6	186
24.2	1505	448	1	121	1063	5	375	5	150
24.4	1536	453	1	120	1085	5	386	5	166
23.9	1621	421	5	110	1084	15	453	15	83
14.4	1666	328	41	77	855	131	855	143	1132
13.5	1577	301	39	76	812	125	812	134	1306

13.4	1569	298	39	76	809	124	809	132	1334
13.3	1637	302	43	73	832	134	832	143	1372
12.2	1493	278	34	76	785	108	785	113	1657
11.9	1494	277	35	75	783	110	783	116	1707
11.9	1494	277	35	75	783	110	783	116	1707
14.2	1624	300	38	76	846	123	846	130	1275
17.9	1499	357	8	106	948	27	948	29	1153
15.1	1437	331	10	103	897	33	897	37	1619
19.8	1613	381	9	103	1013	33	1001	35	803
16.8	1462	338	9	104	917	30	917	33	1318
23.9	2038	485	22	96	1248	80	859	80	57
24.9	1665	428	15	108	1097	49	772	54	35
24.2	1645	420	14	108	1081	47	766	52	138
23.2	1348	356	12	108	895	49	881	53	271
24.2	1635	417	14	108	1073	47	761	52	129
24.3	1650	420	14	108	1083	47	767	52	124
24.1	1612	409	14	107	1053	49	748	54	142
22.7	1502	366	14	103	953	56	950	61	319
20.5	785	205	3	109	534	12	534	12	364
23.1	1165	332	2	114	812	12	634	12	71
22.6	914	292	1	126	664	3	489	3	47
19.4	735	194	4	106	487	16	419	17	561
24.2	1301	404	2	126	964	8	657	8	42
20.7	434	112	3	103	289	13	289	13	56
20.5	785	205	3	109	534	12	534	12	364
23.1	1165	332	2	114	812	12	634	12	71
22.6	914	292	1	126	664	3	489	3	47
19.4	735	194	4	106	487	16	419	17	561
24.2	1301	404	2	126	964	8	657	8	42
18.1	834	168	17	79	459	60	459	67	494
13.3	1544	305	31	79	838	116	838	116	1726
16.7	1650	320	35	77	881	135	881	135	1073
15.5	1652	324	37	76	880	140	880	140	1266
13.7	1568	309	33	78	846	123	846	123	1626
16.9	1663	303	41	70	846	155	846	164	1019
16	1622	297	39	71	832	148	832	153	1196
12.5	1556	284	36	72	800	141	800	142	1878
14.8	1595	286	40	69	805	152	805	158	1427
14.8	1595	286	40	69	805	152	805	158	1427
17.7	1738	297	52	62	832	192	832	214	817
17.4	1712	295	50	63	827	185	827	204	894
22.4	2059	258	69	37	751	263	744	396	24
25.1	1499	455	1	124	1083	6	371	6	14
24.8	1489	457	1	124	1070	5	803	5	13
24.8	1489	457	1	124	1069	5	803	5	12
24.2	1492	438	1	119	1033	6	391	6	172
24.5	1484	436	1	119	1031	6	383	6	112
24.5	1491	458	1	124	1065	6	800	6	40
24.4	1502	446	1	120	1059	6	373	6	114
24.2	1505	448	1	121	1063	5	375	5	150
24.1	1518	450	1	121	1065	5	388	5	218
24.2	1482	446	1	124	1070	5	780	5	15

24.1	1483	447	1	124	1073	5	780	5	22
24	1476	449	1	124	1074	5	780	5	13
24.2	1541	455	1	120	1087	5	390	5	205
24.4	1536	453	1	120	1085	5	386	5	166
24.8	1543	483	1	123	1104	6	372	6	11
24.8	1545	483	1	123	1103	6	374	6	15
24.6	1541	485	1	123	1107	6	368	6	42
23.8	1542	475	1	125	1126	6	817	6	40
23.1	1549	467	1	123	1106	4	386	4	198
24.3	1552	442	2	116	1064	7	429	7	129
23.7	1539	453	1	120	1076	5	397	5	140
24.1	1589	434	2	113	1074	7	468	7	197
23.9	1621	421	5	110	1084	15	453	15	83
23.4	1637	427	5	111	1098	15	848	15	177
22.5	1695	462	3	116	1162	11	881	12	210
18.9	1611	378	4	102	1004	17	518	17	1003
19.4	1591	373	4	102	995	16	513	16	920
24.9	1418	442	1	124	1023	6	324	6	20
23.9	1565	463	2	116	1057	10	428	12	255
24.9	1422	442	1	125	1026	6	326	6	17
24.9	1422	442	1	124	1025	6	326	6	22
24.9	1430	443	1	124	1028	7	329	7	17
24.9	1440	445	1	123	1035	7	333	7	17
24.3	1576	470	2	116	1071	10	427	12	201
24.9	1441	445	1	123	1034	7	335	7	23
24.9	1446	446	1	124	1036	7	338	7	28
20	1607	367	6	102	992	19	809	19	819
19.9	1609	367	6	101	994	19	810	19	839
25	1480	452	1	124	1073	5	649	5	72
25.2	1482	453	1	124	1072	5	366	5	34
24.8	1511	450	1	120	1055	7	390	7	64
24.5	1487	443	2	120	1035	8	379	8	79
14.2	1404	355	5	106	901	18	847	18	1582
23.1	1128	350	3	124	826	12	826	12	37
23.2	1181	357	3	122	852	12	852	12	44
24.2	1209	408	1	136	925	6	570	6	16
24	1293	409	1	129	954	7	614	7	50
23.2	1172	329	2	114	807	11	636	11	67
22.8	1250	340	2	111	845	12	371	12	160
23.4	1320	392	2	123	950	9	637	9	165
23.7	1320	390	2	123	949	9	637	9	94
22.5	1265	340	2	110	850	12	380	12	233
23	1198	332	2	113	817	11	354	11	123
23.1	1185	333	2	113	809	11	347	11	98
22.8	1203	335	2	112	818	11	356	11	168
22.1	1255	339	2	110	844	11	381	11	322
20.7	1389	353	2	107	906	12	436	12	605
24.4	1236	391	1	130	940	7	615	7	16
24.3	1239	393	1	130	942	7	618	7	26
24.2	1253	393	1	129	947	7	624	7	37
24.4	1239	395	1	130	943	7	620	7	8
24.3	1249	397	1	129	947	7	626	7	32

24.3	1278	399	1	128	956	7	642	7	28
24.3	1275	399	1	128	956	7	640	7	29
24.3	1279	400	1	128	957	7	643	7	28
24.3	1259	401	1	130	952	7	634	7	31
24.3	1248	404	1	130	949	7	588	7	38
24.5	1263	409	1	130	959	6	643	6	2
23.7	1356	409	2	121	976	9	691	9	139
23.8	1348	411	2	123	977	9	689	9	121
24.6	1261	411	1	130	958	6	590	6	5
18.9	1611	378	4	102	1004	17	518	17	1003
18.9	1610	378	4	102	1004	17	518	17	1008
18.8	1614	379	4	102	1006	17	519	17	1025
18.6	1623	381	4	102	1011	17	522	17	1078
19.4	1591	373	4	102	995	16	513	16	920
24.7	1293	418	1	129	971	6	599	6	3
23.1	1528	442	2	113	1022	12	414	13	362
24.7	1396	438	1	125	1013	6	313	6	50
24.8	1404	439	1	125	1016	6	318	6	34
24.7	1425	442	1	124	1029	7	326	7	41
24.9	1413	441	1	125	1022	6	321	6	23
24.9	1410	440	1	125	1020	6	320	6	13
24.9	1409	440	1	125	1018	6	320	6	15
23.9	1565	463	2	116	1057	10	428	12	255
24.9	1422	442	1	125	1026	6	326	6	17
24.9	1430	443	1	124	1028	7	329	7	17
24.1	1573	468	2	116	1069	10	426	12	218
24.8	1447	446	1	124	1040	7	337	7	28
24.9	1440	445	1	123	1035	7	333	7	17
24.3	1573	469	2	116	1070	10	425	12	186
24.8	1448	446	1	124	1040	7	338	7	30
24.5	1571	470	2	117	1073	10	422	12	145
24.9	1441	445	1	123	1034	7	335	7	23
24.9	1446	446	1	124	1036	7	338	7	28
24.6	1588	477	2	117	1082	9	432	12	156
25	1480	452	1	124	1073	5	649	5	72
12.7	1424	270	30	80	770	94	770	100	1616
13.7	1551	288	35	78	822	113	822	121	1377
14.8	1358	316	15	97	821	55	796	61	1279
14.4	1313	311	13	99	802	49	779	55	1353
15.8	1701	347	30	85	947	115	914	127	996
18	2217	376	52	64	1074	206	1036	299	635
17.7	2289	383	54	62	1088	224	1088	329	683
16	1655	318	35	79	896	134	896	144	951
15.5	1807	324	51	68	903	179	900	244	1012
15.8	1778	322	49	70	909	169	909	195	993
15.6	1889	334	53	66	930	192	930	265	985
15.2	1832	324	52	66	905	184	903	252	1067
14.7	1780	314	51	67	887	175	880	240	1156
16	1992	351	56	65	987	208	987	286	937
16	1245	261	15	88	718	81	718	81	1045
16.1	1220	254	14	89	703	77	703	77	1025
16.1	1230	263	15	87	700	79	690	100	979

16.1	1819	319	48	68	928	177	928	209	972
15.5	1376	324	18	96	831	75	828	79	1126
19.6	2586	461	71	57	1232	306	1232	386	341
18.8	1825	317	45	65	912	173	912	201	535
19.2	2219	353	66	55	1028	248	1028	310	497
17	1659	300	40	68	841	156	841	185	769
20	2595	372	76	47	1094	295	1094	408	262
19.7	2409	351	75	47	1025	275	1025	385	369
16	1182	263	25	89	685	81	685	86	931
16.5	1223	269	27	88	697	87	697	94	829
13.7	1232	269	24	91	733	75	733	80	1391
12.7	1280	271	24	90	761	76	761	82	1599
14.8	1399	309	30	86	803	99	803	104	1098
15.3	1520	316	37	81	833	123	833	133	986
15.1	1508	317	36	82	833	121	833	130	1034
15.4	1557	321	38	80	846	128	846	141	973
14.2	1504	314	35	79	806	117	806	136	1183
19.4	2260	378	64	65	1124	238	1045	292	164
15.4	1626	332	40	78	872	134	872	153	953
14.7	1570	332	37	80	858	124	858	140	1081
14.8	1631	343	39	79	870	127	870	151	1052
14.1	1620	315	40	76	833	129	833	139	1201
13.5	1571	300	39	76	811	124	811	132	1311
14.4	1666	328	41	77	855	131	855	143	1132
13.5	1577	301	39	76	812	125	812	134	1306
14.1	1638	318	41	76	839	132	839	143	1192
16.1	1895	394	44	77	1012	149	988	188	801
14.3	1617	330	40	77	845	130	845	158	1141
14.4	1663	332	42	75	854	134	850	165	1134
15.3	1731	350	44	76	912	143	912	172	948
16.6	1948	386	49	75	1034	162	999	204	690
16.7	2025	408	47	76	1081	164	1040	211	672
16.9	2159	435	46	75	1151	174	1090	234	639
14.3	1709	338	43	74	873	142	868	180	1137
17.1	2150	445	43	75	1132	172	1049	231	661
12.9	1582	293	39	74	814	123	814	130	1469
17.1	2322	429	54	69	1199	209	1113	287	571
16.4	1829	333	46	75	934	142	932	163	896
16.2	2038	373	50	70	1010	174	976	227	841
20.3	2557	417	88	50	1131	303	1131	434	4
18.7	2353	392	57	59	1114	234	1006	355	427
20.8	2664	408	74	48	1146	298	1146	501	24
23.9	1633	415	14	107	1070	47	760	52	171
21.4	1446	330	16	97	883	67	871	73	513
23.3	1414	340	17	99	879	68	879	69	213
24	1362	316	21	93	821	81	821	84	101
23.9	1366	314	21	93	820	82	820	85	122
23.4	1368	306	22	90	809	85	809	89	181
23.4	1368	306	22	90	809	85	809	89	181
23.1	1325	295	22	89	777	86	777	93	223
23.4	1522	332	20	93	908	77	669	86	195
24.4	1503	339	21	93	903	80	659	80	45

21.8	1435	279	26	80	789	106	789	106	385
23.7	1426	277	30	77	764	119	764	121	82
23.5	1418	274	30	77	763	118	763	120	109
22.4	1397	265	28	77	753	113	753	117	283
21	1386	252	29	73	726	119	697	125	497
23.3	1365	243	35	68	689	134	689	144	122
21	465	138	1	119	336	8	309	8	12
20.7	434	112	3	103	289	13	289	13	56
21	590	167	1	119	429	6	396	6	66
21	416	95	5	91	259	21	259	25	2
20.8	424	96	5	90	263	21	263	25	27
20.8	426	97	5	91	265	21	265	25	43
21.5	658	198	1	128	493	4	493	4	25
20.4	400	98	4	95	258	17	258	17	128
20.5	418	103	4	97	270	17	270	18	120
21.5	687	210	1	126	513	4	513	4	37
21	739	187	2	111	508	10	508	10	143
21.1	743	188	2	111	509	10	509	10	139
20.3	450	97	6	87	271	25	271	31	93
21.8	713	241	0	134	545	2	545	2	4
21.8	713	240	0	134	544	2	544	2	2
20.2	454	96	6	86	271	26	271	33	118
21.7	726	242	1	133	551	3	551	3	26
21.6	735	240	1	130	553	3	553	3	55
21.7	730	243	1	133	553	3	553	3	31
19.7	524	133	4	101	344	17	344	17	306
20.7	476	120	4	100	311	17	311	18	87
20.9	786	224	1	118	560	6	560	6	216
21.5	756	205	2	113	528	9	528	9	102
20	526	134	4	102	345	17	345	18	250
21.6	742	198	2	113	516	9	516	9	74
21.6	746	201	2	113	520	9	520	9	75
21.5	755	205	2	115	528	9	528	9	97
21.5	757	206	2	113	529	9	529	9	102
21.4	761	207	2	114	532	9	532	9	108
21.7	749	249	1	132	565	3	565	3	56
15.7	800	201	5	106	532	17	532	17	1325
20.8	784	204	3	109	534	12	534	12	290
17.1	759	193	5	105	505	18	505	18	1029
21.2	796	221	2	115	557	9	557	9	170
19.9	537	129	5	97	342	21	342	25	289
20.9	823	228	2	115	574	9	574	9	259
21	801	215	2	112	552	10	552	10	259
21.2	837	245	1	120	595	6	595	6	212
21.2	828	246	1	120	594	5	594	5	200
21.9	781	267	0	133	592	2	588	2	49
21.9	780	277	0	136	605	1	594	1	28
22	787	275	0	136	606	2	595	2	38
22.2	825	289	0	136	633	2	622	2	12
20.8	877	253	2	118	622	7	495	7	329
20.2	567	135	5	95	359	22	359	27	246
20.8	737	200	3	109	504	14	422	15	385

23.1	1049	314	2	119	753	9	578	9	31
22.5	865	288	1	129	645	3	637	3	17
19.9	446	90	8	81	257	30	257	39	109
22.7	917	295	1	126	667	3	491	3	21
20.7	631	156	5	100	405	20	405	23	129
20	447	90	8	82	258	30	258	39	67
20.7	631	156	5	100	405	20	405	23	131
22.7	913	290	1	125	662	3	487	3	47
22.7	920	291	1	126	664	3	492	3	56
19.8	728	194	4	107	486	16	414	17	483
20.7	893	252	2	118	635	7	506	7	391
22.7	925	290	1	124	665	3	494	3	65
22.3	873	268	1	124	631	4	470	4	115
19.5	754	200	4	109	510	17	444	17	665
18.1	753	192	5	105	494	19	435	19	862
20.8	878	243	2	116	622	8	500	8	374
22.7	907	271	1	122	648	4	486	4	94
21.1	875	241	2	117	621	8	498	8	325
19.1	742	192	5	106	489	18	430	19	696
18.7	750	193	6	106	496	22	444	22	807
21.6	942	264	2	118	675	7	524	7	256
20.9	772	204	2	111	535	11	447	11	345
20.8	764	202	3	111	525	12	443	12	374
23.5	1136	328	2	116	774	9	326	9	35
21	764	202	3	111	527	12	442	12	324
21.5	804	210	2	113	567	10	457	10	229
19.2	754	192	4	104	493	14	432	14	703
19.4	765	197	3	105	504	11	438	11	667
19.5	765	199	3	106	508	12	441	12	631
19.8	761	200	3	107	509	11	442	11	572
21.4	915	242	3	114	645	11	522	11	260
18.6	792	201	2	103	517	9	517	9	841
21	951	253	3	114	670	12	547	12	334
21.5	966	256	4	114	677	14	549	14	228
21.6	959	254	4	114	671	14	545	14	200
21.4	982	261	4	114	687	14	560	14	241
19.9	1020	276	3	114	718	12	597	12	547
20	683	138	11	82	383	38	383	53	124
20.1	598	106	13	70	310	45	310	69	60
19.4	628	119	12	76	341	42	341	60	251
19.6	615	114	12	73	329	43	329	63	199
19.5	619	115	12	74	331	43	331	63	220
20.2	561	90	16	58	264	57	264	88	27
20.4	581	87	19	52	257	67	257	103	20
20.4	566	79	21	46	236	71	216	114	24
20.1	649	106	18	61	310	62	310	91	81
20.3	586	84	21	48	250	72	230	111	64
19.3	767	142	19	71	395	63	395	82	242
18.3	777	124	28	53	351	95	351	122	466
20	838	137	26	58	383	92	383	124	78
20.1	800	123	28	51	342	97	339	139	80
13.1	1347	298	11	96	817	36	817	36	1616

13	1351	298	11	96	818	36	818	36	1636
17.7	879	148	30	58	415	99	415	120	594
19.7	951	155	31	56	428	109	426	144	119
19.2	891	140	32	51	386	111	386	149	259
18.9	864	127	35	42	349	119	340	165	307
19.6	1242	187	46	49	540	165	500	225	112
18	1071	192	35	64	526	110	526	127	456
19.1	1113	162	45	42	449	157	449	216	227
19.8	1611	257	60	47	700	223	615	308	25
17.7	1968	344	56	63	964	201	964	234	773
21.2	2715	383	68	49	1132	274	1132	418	77
19.2	2284	352	62	55	1035	246	1035	317	468
20.5	2878	401	71	46	1145	288	1145	473	172
21.1	3285	442	79	44	1279	319	1202	589	8
18	2250	318	67	47	944	259	944	360	673
19.2	2427	331	71	43	952	277	952	421	434
18.2	2232	309	68	45	910	262	910	373	640
21.1	2744	371	84	40	1040	311	1003	513	101
21.3	2745	371	85	40	1039	311	998	518	60
20.8	2513	328	76	39	940	294	907	472	180
20.2	2383	312	74	38	887	285	870	446	296
21.6	2507	325	79	38	931	298	879	486	55
17.5	1129	251	23	88	643	77	641	86	631
15.3	1520	316	37	81	833	123	833	133	986
15.1	1508	317	36	82	833	121	833	130	1034
15.4	1557	321	38	80	846	128	846	141	973
15.4	1626	332	40	78	872	134	872	153	953
14.7	1570	332	37	80	858	124	858	140	1081
14.8	1631	343	39	79	870	127	870	151	1052
16.1	1895	394	44	77	1012	149	988	188	801
15.3	1731	350	44	76	912	143	912	172	948
16.6	1948	386	49	75	1034	162	999	204	690
16.7	2025	408	47	76	1081	164	1040	211	672
13.3	1632	302	42	73	832	131	832	140	1374
13.3	1637	302	43	73	832	134	832	143	1372
20.2	2380	340	81	45	1001	314	1001	413	23
20.2	2382	339	82	44	998	316	998	414	21
14.8	1368	317	15	96	825	57	799	63	1266
18.1	2470	417	61	59	1172	257	1056	373	429
20	2530	408	83	49	1128	312	1128	454	22
15.7	1928	334	50	67	959	183	940	253	975
15.5	1807	324	51	68	903	179	900	244	1012
15.8	1778	322	49	70	909	169	909	195	993
15.2	1832	324	52	66	905	184	903	252	1067
14.7	1780	314	51	67	887	175	880	240	1156
16	1992	351	56	65	987	208	987	286	937
15.4	1418	280	24	83	790	104	790	107	1108
15.8	1430	281	24	82	797	105	797	108	1034
16.1	1476	282	27	78	806	117	806	126	977
16.1	1220	254	14	89	703	77	703	77	1025
16.1	1230	263	15	87	700	79	690	100	979
16.1	1819	319	48	68	928	177	928	209	972

15.5	1376	324	18	96	831	75	828	79	1126
19.6	2586	461	71	57	1232	306	1232	386	341
14.3	1561	284	35	71	811	142	811	164	1309
18.8	2481	423	71	55	1156	294	1156	374	493
16.5	1436	279	27	76	763	116	763	133	875
18.8	1825	317	45	65	912	173	912	201	535
19.6	2479	418	72	54	1145	292	1145	371	362
18.3	1742	308	40	67	880	160	880	186	622
19.9	1998	336	55	61	963	203	963	234	305
19.5	2279	366	68	55	1055	256	1055	320	427
16.8	1612	357	27	88	935	105	935	105	1045
16.7	1614	356	28	87	932	108	932	108	1061
16.3	1598	350	28	86	919	110	919	110	1106
16.3	1598	350	28	86	919	110	919	110	1107
19	2147	337	65	54	983	239	983	297	536
19.4	2332	355	72	50	1034	273	1034	345	460
17.7	1773	340	42	73	939	154	939	175	815
18.1	1935	352	48	68	986	177	986	206	720
18.9	2154	352	57	60	1023	216	1023	272	548
19.7	2316	364	61	57	1068	237	1068	306	397
18.8	2146	353	58	60	1020	217	1020	271	575
19.5	2346	361	62	55	1062	248	1062	323	414
19.8	2432	365	63	53	1077	254	1077	347	340
19.7	2409	351	75	47	1025	275	1025	385	369
18.3	2247	310	69	44	907	265	907	380	622
